# Sintilimab (anti-PD-1 antibody) plus chidamide (histone deacetylase inhibitor) in relapsed or refractory extranodal natural killer T-cell lymphoma (SCENT): a phase Ib/II study

**DOI:** 10.1038/s41392-024-01825-0

**Published:** 2024-05-17

**Authors:** Yan Gao, Haixia He, Xueping Li, Liling Zhang, Wei Xu, Ru Feng, Wenyu Li, Yin Xiao, Xinxiu Liu, Yu Chen, Xiaoxiao Wang, Bing Bai, Huijing Wu, Qingqing Cai, Zhiming Li, Jibin Li, Suxia Lin, Yanxia He, Liqin Ping, Cheng Huang, Jiaying Mao, Xiujin Chen, Baitian Zhao, Huiqiang Huang

**Affiliations:** 1 State Key Laboratory of Oncology in South China & Collaborative Innovation Center of Cancer Medicine, Guangdong Provincial Clinical Research Center for Cancer, Sun Yat-sen University Cancer Center, Guangzhou, China; 2 Department of Medical Oncology, Sun Yat-sen University Cancer Center, Guangzhou, China; 3 Department of Radiation Oncology, Sun Yat-sen Memorial Hospital, Sun Yat-sen University, Guangzhou, China; 4 Department of Hematology, Sun Yat-sen University Cancer Center, Guangzhou, China; 5 Cancer Center, Union Hospital affiliated to Tongji Medical College of Huazhong University of Science and Technology, Wuhan, China; 6 Department of Hematology, The First Affiliated Hospital of Nanjing Medical University, Jiangsu Province Hospital, Nanjing, China; 7 Department of Hematology, Nanfang Hospital, Southern Medical University, Guangzhou, China; 8 Department of Lymphoma, Guangdong Provincial People’s Hospital, Guangdong Academy of Medical Sciences, Guangzhou, China; 9 Department of pathology, Guangdong General Hospital, Guangdong Academy of Medical Sciences, Guangzhou, China; 10 Department of Medical Oncology, Hubei Cancer Center, Wuhan, China; 11 Department of Clinical Research, Sun Yat-sen University Cancer Center, Guanzhou, China; 12 Department of Pathology, Sun Yat-sen University Cancer Center, Guangzhou, China

**Keywords:** Haematological cancer, Clinical trials, Predictive markers

## Abstract

Anti-PD-1 antibodies are a favorable treatment for relapsed or refractory extranodal natural killer T cell lymphoma (RR-ENKTL), however, the complete response (CR) rate and the duration of response (DOR) need to be improved. This phase 1b/2 study investigated the safety and efficacy of sintilimab, a fully human anti-PD-1 antibody, plus chidamide, an oral subtype-selective histone deacetylase inhibitor in 38 patients with RR-ENKTL. Expected objective response rate (ORR) of combination treatment was 80%. Patients received escalating doses of chidamide, administered concomitantly with fixed-dose sintilimab in 21-days cycles up to 12 months. No dose-limiting events were observed, RP2D of chidamide was 30 mg twice a week. Twenty-nine patients were enrolled in phase 2. In the intention-to-treat population (n = 37), overall response rate was 59.5% with a complete remission rate of 48.6%. The median DOR, progression-free survival (PFS), and overall survival (OS) were 25.3, 23.2, and 32.9 months, respectively. The most common grade 3 or higher treatment-emergent adverse events (AEs) were neutropenia (28.9%) and thrombocytopenia (10.5%), immune-related AEs were reported in 18 (47.3%) patients. Exploratory biomarker assessment suggested that a combination of dynamic plasma ctDNA and EBV-DNA played a vital prognostic role. STAT3 mutation shows an unfavorable prognosis. Although outcome of anticipate ORR was not achieved, sintilimab plus chidamide was shown to have a manageable safety profile and yielded encouraging CR rate and DOR in RR-ENKTL for the first time. It is a promising therapeutic option for this population.

## Introduction

Extranodal natural killer (NK)/T-cell lymphoma, nasal type (ENKTL) is a rare aggressive type of non-Hodgkin’s lymphoma associated with Epstein-Barr virus infection,^1^ it is more prevalent in Asia than in other regions, accounting for ~6% of lymphoma subtypes in China.^2^ Newly diagnosed advanced and relapse or refractory (RR)-ENKTL cases are characterized by a high rate of relapse and dismal prognosis with limited therapeutic options,^3,4^ the 5-year survival rate of RR-ENKTL is less than 30%.^5,6^ It is difficult to break through the bottleneck of curative effects in the era of chemotherapy. Immune evasion and abnormal epigenetic regulation are important molecular genetic characteristics of ENKTL.^7,8^ The use of immune checkpoint inhibitors (ICIs), represented by programmed cell death protein 1 (PD-1) antibodies, have produced a unique and significant remission rate in RR-ENKTL in recent years.^9–12^ However, the complete remission (CR) rate reached only about 30%, and the remission period was short. Therefore, it is necessary to explore new combinations of therapeutics with immunotherapy. Recent preclinical studies on epigenetics in immune evasion have clarified the major role of epigenetic modulators in augmenting the tumor microenvironment and restoring immune recognition and immunogenicity. Moreover, accumulating evidence has revealed synergisms between epigenetic drugs and anti-PD-1 antibodies.^13^ Therefore, incorporating epigenetic modulators may be a potential therapeutic strategy to improve the efficacy of immunotherapy in RR-ENKTL.

Sintilimab is a fully human anti-PD-1 antibody, and the ORIENT-4 trial^10^ demonstrated that it is effective and well tolerated in RR-ENKTL patients. Chidamide, an oral subtype-selective histone deacetylase inhibitor (HDACi), has been approved by the National Medical Products Administration (NMPA) for treating refractory or relapsed peripheral T-cell lymphoma, including ENKTL.^14,15^ We demonstrated that chidamide as single agent is effective in treating RR-ENKTL with objective response rate (ORR) and CR rate of 39% and 18%, respectively. Aberrant JAK-STAT signaling-mediated chromatin remodeling impairs the sensitivity of NK/T-cell lymphoma to chidamide.^16^ The purpose of this trial was to evaluate the efficacy and safety of the combination of sintilimab plus chidamide (SC) in patients with RR-ENKTL.

## Results

### Patient characteristics

**Fig. 1:**
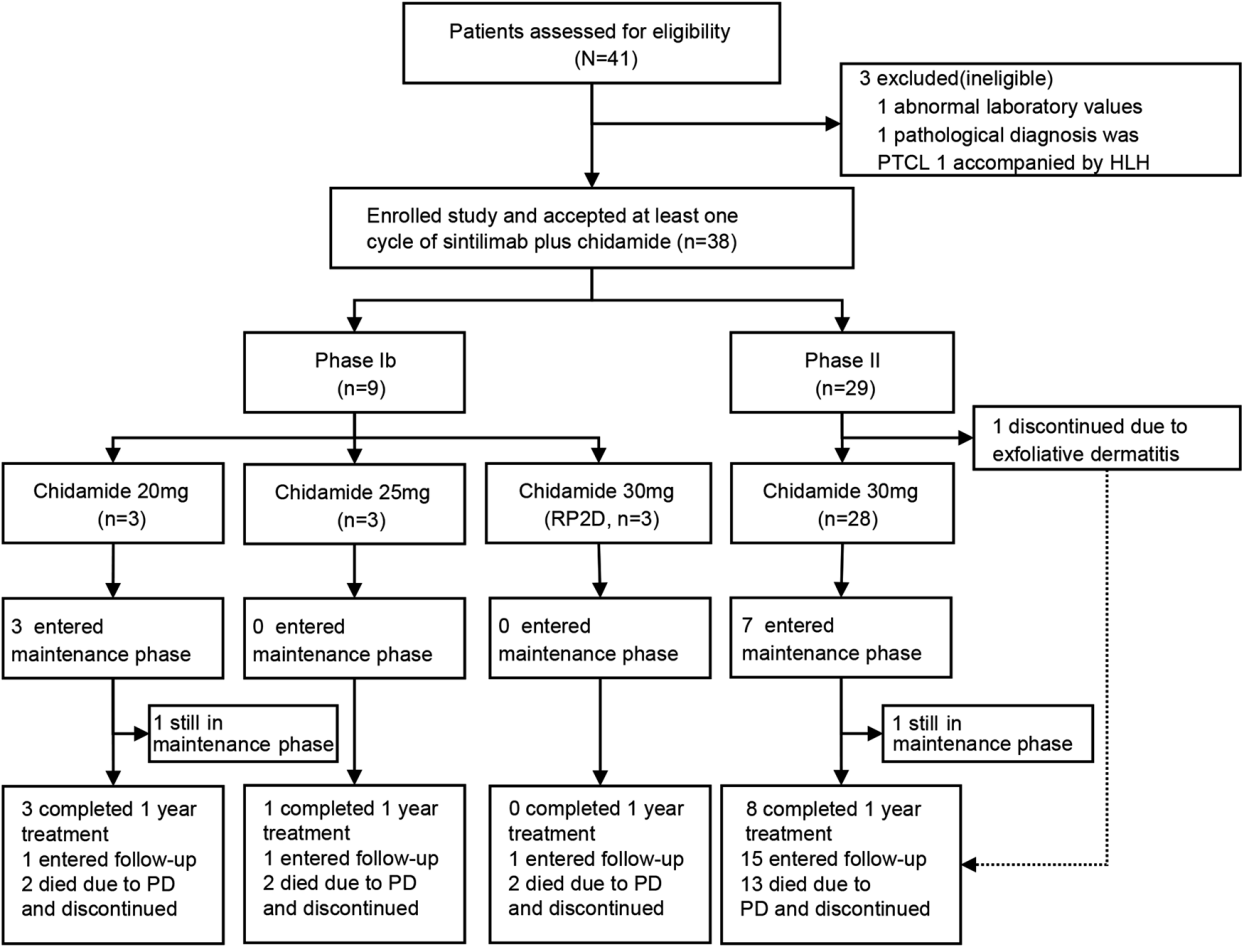
Patient Disposition. PTCL peripheral T-cell lymphoma, HLH hemophagocytic lymphohistiocytosis, RP2D recommended phase 2 dosage, PD progression disease

Between March 29, 2017, and August 26, 2020, 41 patients were screened; of these, 38 eligible patients (as-treated population) were enrolled and accepted at least one cycle of treatment from 6 institutions (Fig. 1). All 38 patients were included in the safety analysis, and the intention-to-treat population (n = 37) included 31 patients in the activity-evaluable population, which was treated with the recommended phase 2 dose (RP2D). The last follow-up time was October 30, 2023. Baseline characteristics are listed in Table 1. Overall, the median age was 43 years (range, 20–72), 25 (65.8%) with stage III-IV disease at screening, 22 (57.9%) had a prognostic index for NK cell lymphoma-EBV (PINK-E) score ≥3, and 17 (44.7%) received ≥2 lines of prior systemic therapy. The median time from last treatment to this trial was 6.5 months (0.9–82.9). Fourteen (36.8%) patients had a median interval of less than 3-months from final treatment to enrollment (prior treatment regimens in the Supplementary Table S1).

**Table 1:** Clinical characteristics of the study population

Characteristic	Chidamide dosage			Total (n = 38)
No. (%)	20 mg (n = 3)	25 mg (n = 3)	30 mg (n = 32)	
Age, years				
Median (range)	37 (20–53)	37 (27–51)	48 (21–72)	43 (20–72)
<60	3 (100)	3 (100)	27 (84.3)	33 (86.8)
≥60	0	0	5 (15.7)	5 (13.2)
Sex				
Male	2 (66.7)	2 (66.7)	24 (75.0)	28 (73.7)
Female	1 (33.3)	1 (33.3)	8 (25.0)	10 (26.3)
ECOG performance status at study entry				
0	3 (100)	1 (33.3)	14 (43.8)	18 (47.4)
1	0	2 (66.7)	14 (43.8)	16 (42.1)
2	0	0	4 (12.4)	4 (10.5)
Ann Arbor stage				
I–II	1 (33.3)	1 (33.3)	11 (34.4)	13 (34.2)
III–IV	2 (66.7)	2 (66.7)	21 (65.6)	25 (65.8)
Fever at study entry				
Yes	2 (66.7)	1 (33.3)	10 (31.3)	13 (34.2)
No	1 (33.3)	2 (66.7)	22 (68.7)	25 (65.8)
Plasma lactate dehydrogenase level at study entry				
Normal	0	2 (66.7)	17 (53.1)	19 (50.0)
Elevated	3 (100)	1 (33.3)	15 (46.9)	19 (50.0)
Lesion’s location				
Nasal	0	0	15 (46.9)	15 (40.0)
Non-nasal	3 (100)	3 (100)	17 (53.1)	23 (60.0)
Bone marrow involvement				
Yes	0	1 (33.3)	4 (12.5)	5 (13.1)
No	3 (100)	2 (66.7)	28 (87.5)	33 (86.9)
Distant lymph node involvement				
Yes	2 (66.7)	1 (33.3)	10 (31.3)	13 (34.2)
No	1 (33.3)	2 (66.7)	22 (68.7)	25 (65.8)
Prior HLH				
Yes	2 (66.7)	0	1 (3.1)	3 (7.9)
No	1 (33.3)	3 (100)	31 (96.9)	35 (92.1)
Plasma EBV-DNA level				
Non-detectable	0	0	9 (28.1)	9 (23.7)
Detectable	3 (100)	3 (100)	23 (71.9)	29 (76.3)
PINK-E score^4^				
Low (0–1)	0	1 (33.3)	10 (31.3)	11 (29.0)
Intermediate (2)	1 (33.3)	0	4 (12.5)	5 (13.1)
High (≥3)	2 (66.7)	2 (66.7)	18 (56.2)	22 (57.9)
Disease status at study entry				
Relapsed	1 (33.3)	1 (33.3)	12 (37.5)	14 (36.8)
Refractory	2 (66.7)	2 (66.7)	20 (62.5)	24 (63.2)
Relapse or disease progression within 3 months from last therapy				
Yes	1 (33.3)	0	13 (40.6)	14 (36.8)
No	2 (66.7)	3 (100)	19 (59.4)	24 (63.2)
Previous systemic therapies				
Median (IQR)	NA	NA	1 (1–2)	1 (1–2)
One line	2 (66.7)	2 (66.7)	17 (53.1)	21 (55.3)
Two lines	1 (33.3)	1 (33.3)	8 (25.0)	10 (26.3)
More than three lines	0	0	7 (21.9)	7 (18.4)
Prior anti-PD-1/L1 antibody therapy^a^				
Yes	1 (33.3)	0	5 (15.7)	6 (15.8)
No	2 (66.7)	3 (100)	27 (84.3)	32 (84.2)
Prior chidamide therapy				
Yes	0	0	4 (12.5)	4 (10.5)
No	3 (100)	3 (100)	28 (87.5)	34 (89.5)
Prior ASCT therapy				
Yes	0	0	2 (6.3)	2 (5.3)
No	3 (100)	3 (100)	30 (93.7)	36 (94.7)
Previous radiotherapy				
Yes	1 (33.3)	2 (66.7)	21 (65.6)	24 (63.2)
No	2 (66.7)	1 (33.3)	11 (34.4)	14 (36.8)

Data are n (%), unless otherwise indicated

*ASCT* autologous stem cell transplantation, *EBV* Epstein-Barr virus, *ECOG* eastern cooperative oncology group, *HLH* hemophagocytic lymphohistiocytosis, *LDH* lactate dehydrogenase, *NA* not applicable, *PINK-E* prognostic index for natural killer cell lymphoma–Epstein-Barr virus

^a^Anti-PD-1/L1 antibody including pembrolizumab, sintilimab, sugemalimab^31^

### Efficacy

Nine patients were enrolled in phase 1b, and three patients in each cohort were treated with 20, 25, and 30 mg (the maximum dosage) of chidamide, respectively, in combination with fixed doses of 200 mg of sintilimab. No dose-limiting toxicities (DLTs) were observed, and the maximum tolerated dose (MTD) was not reached even at the 30 mg dose. Therefore, the RP2D of chidamide was 30 mg twice a week. Twenty-nine additional patients enrolled in phase 2 were treated with chidamide as RP2D in combination fixed doses of 200 mg of sintilimab. Thus, a total of 32 patients were treated with chidamide at a dosage of 30 mg.

**Fig. 2:**
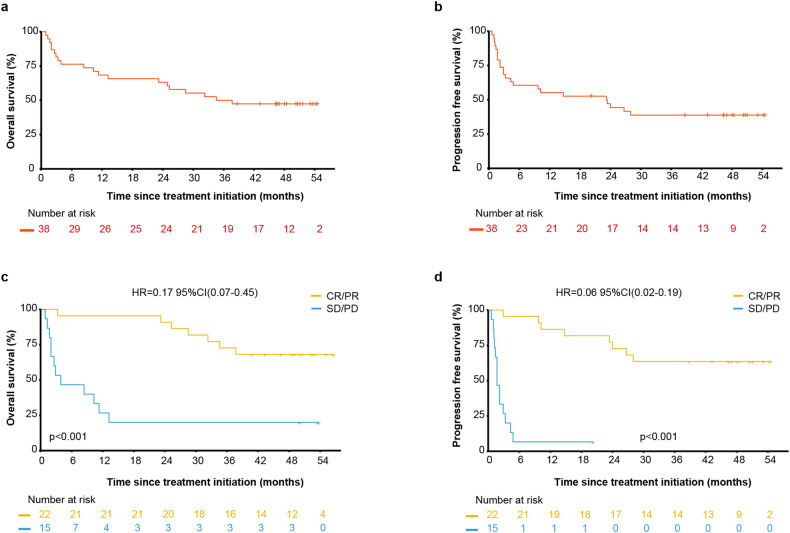
Investigators-assessed survival outcomes. **a** Overall survival of patients (as-treated population, N = 38). **b** Progression-free survival of patients (as-treated population, N = 38). **c** Overall survival of patients with CR or PR versus SD or PD (intention-to-treat population, N = 37). **d** Progression-free survival of patients with CR or PR versus SD or PD (intention-to-treat population, N = 37)

**Fig. 3:**
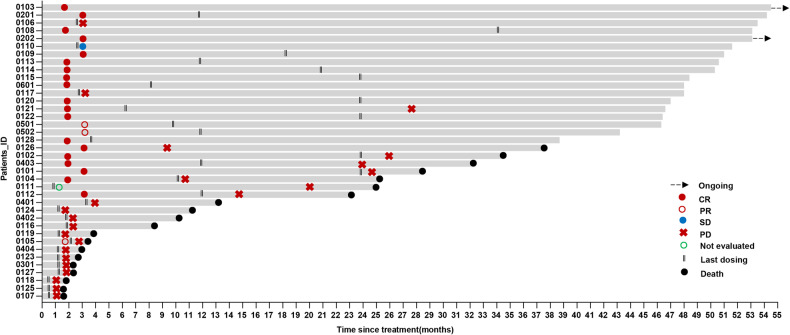
Swimmer plots showing timing of response, as assessed by investigators (as-treated population, N = 38). Bars represent individual patients

In phase 1b, 6 of the 9 patients had a response (ORR: 66.7%), and 5 (55.6%) had CR. In the first stage of phase 2, 8 patients were enrolled. Efficacy was evaluable in 7 patients, as one patient discontinued treatment after the first cycle due to grade 4 exfoliative dermatitis. The ORR was 85.7% (6/7), with 5 patients (71.4%) achieving CR, exceeding the prespecified efficacy boundary for enrollment in the second stage. Among all 28 patients in phase 2, 16 (10 in stage two) had a response, and the ORR was 57.1% (16/28), with a CR rate of 46.4% (13/28). In the intention-to-treat population (n = 37), 22 patients (59.5%) achieved an objective response, including 18 patients (48.6%) with CR (Table 2). Ten patients who had previously treated with ICIs (6 patients) or chidamide (4 patients) were enrolled. ORR and CR were observed in 6(60.0%, 6/10) and 4(40.0%, 4/10) patients after SC treatment (Supplementary Table S2). The median follow-up time was 38.7 months (0.9–54.5). Among the 22 patients who responded to SC, the median time to response (TTR) was 1.5 months (1.2–1.8). Median DOR was 25.3 months (22.2–53.0). The median PFS of the as-treated population was 23.2 months (95% CI: 18.9–33.9), and the median OS was 32.9 months (95% CI: 25.8–40.1) (Fig. 2a, b), 36-month OS and PFS rate were 47.4% (95%CI 32.0–63.4%), 38.8% (95%CI 24.2–55.2%), respectively. Survival of patients who achieved CR or partial remission (PR) significantly better than patients with stable disease (SD) or progression disease (PD), (Fig. 2c, d). Multivariate analysis showed eastern cooperative oncology group (ECOG) score of 2 (*p* = 0.008), bone marrow involvement (*p* = 0.032) were associated with poor OS. ECOG score of 2 (*p* = 0.018), distant lymph node involvement (*p* = 0.044) were associated with poor PFS (Supplementary Fig. S1, Supplementary Table S3). Two (5.2%) patients were still accepted SC as maintenance, no patients accepted autologous hematopoietic stem cell transplantation (ASCT) (Fig. 3). The median cycles of the as-treated population and responders lasting more than 1 year were 6 (1–46) and 25 (18–46), respectively. Three (7.8%) patients experienced hyperprogressive disease (HPD), and the median OS of HPD was 1.3 months (0.9–1.9 months).

**Table 2:** Best overall response

No. (%)	Fixed doses of 200 mg sintilimab combined with escalated chidamide:			
	Dose level 1 chidamide 20 mg (n = 3)	Dose level 2 chidamide 25 mg (n = 3)	Dose level 3 Chidamide (RP2D) 30 mg (n = 31)	Intention-to-treat population (n = 37)
ORR	3 (100)	3 (100)	16 (51.6)	22 (59.5)
CR	3 (100)	2 (66.7)	13 (41.9)	18 (48.6)
PR	0	1 (33.3)	3 (9.7)	4 (10.8)
SD	0	0	2 (6.5)	2 (5.4)
PD	0	0	13 (41.9)	13 (35.1)

Responses were assessed according to the the International Working Group consensus response evaluation criteria in lymphoma (RECIL 2017)

*CR* complete remission, *RP2D* recommended phase 2 dosage, *ORR* objective response rate, *PD* rogressive disease, *PR* partial remission, *SD* stable disease

### Exploratory biomarker assessment

**Fig. 4:**
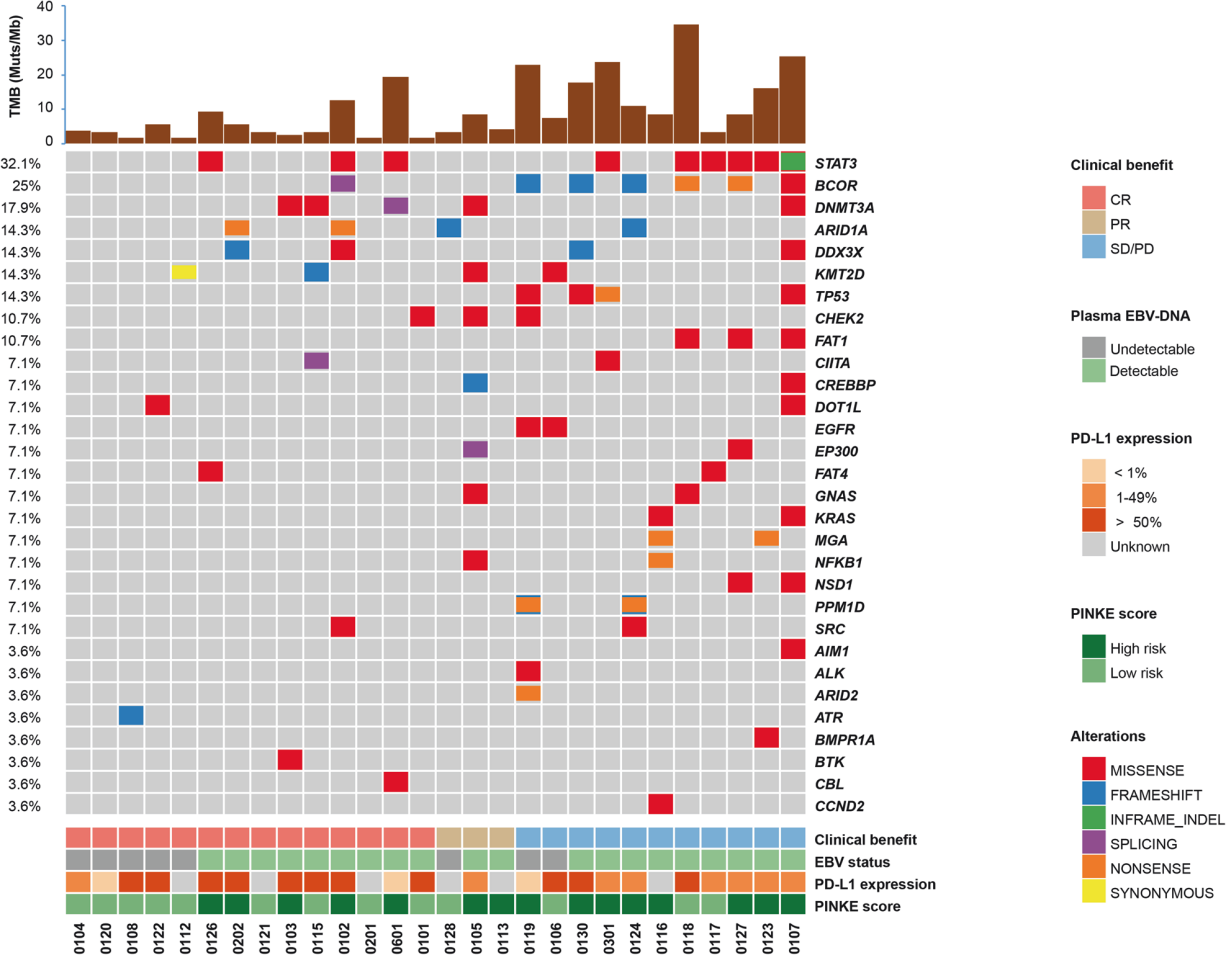
Genetic alteration of patients with RR-ENKTL in peripheral blood. Clinical information was indicated by bars on bottom. Each column represented one case

**Fig. 5:**
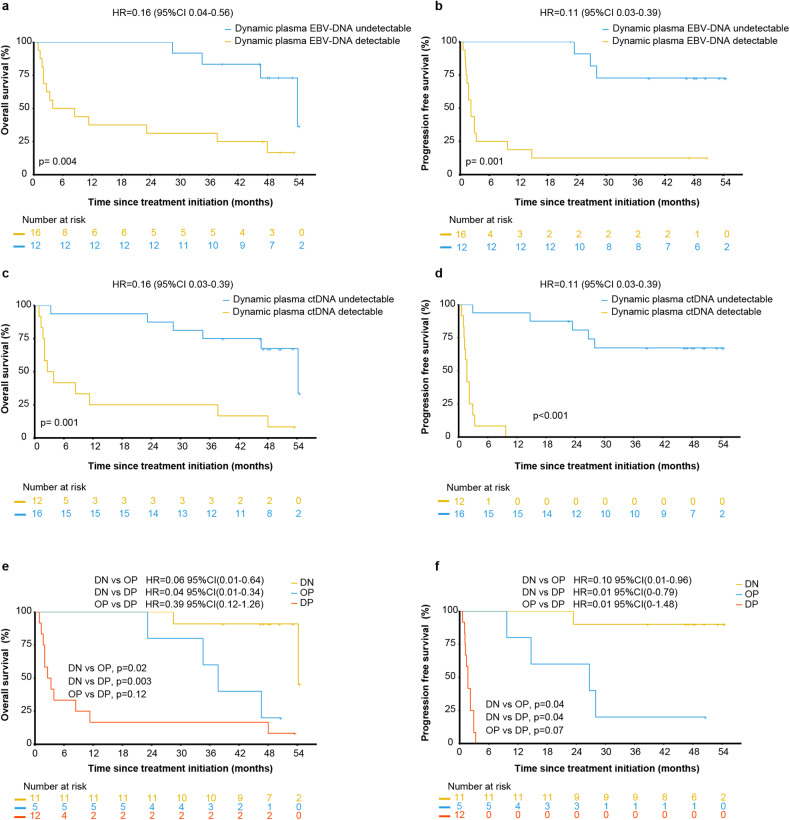
Dynamics changes of plasma EBV-DNA, ctDNA, and the prognosis of OS and PFS. **a** Dynamics changes of plasma EBV-DNA clearance with OS. **b** Dynamics changes of plasma EBV-DNA clearance with PFS. **c** Dynamics changes of plasma ctDNA clearance with OS. **d** Dynamics changes of plasma ctDNA clearance with PFS. **e** OS of dynamics changes of plasma ctDNA and EBV-DNA with double negative (DN), one positive (OP), and double positive (DP). **f** PFS of dynamics changes of plasma ctDNA and EBV-DNA with DN, OP, and DP

Among the 28 patients with available formalin-fixed paraffin-embedded tissue specimens and peripheral blood, 12 (42.8%) were observed to have PD-L1 expression in more than 50%, receiver operating characteristic (ROC) curves for predicting ORR were used to determine cutoff values. The median and cutoff value of the combined positive score (CPS) were 50 and 32.5, respectively. Median tumor mutation burden (TMB) was 5.6 mut/Mb (1.7-34.7), the cutoff value was 6.5 mut/Mb. Frequent (>10%) mutations in pretreatment peripheral blood occurred in *STAT3* (n = 9), *BCOR* (n = 7), *DNMT3A* (n = 5), *ARID1A* (n = 4), *DDX3X* (n = 4), *KMT2D* (n = 4), *TP53* (n = 4), *CHEK2* (n = 3), and *FAT1* (n = 3), Fig. 4. Univariate analyses identified that patients with PD-L1 expression in ≥25%, CPS ≥ 32.5 and TMB < 6.5 Mb achieved a higher ORR than their counterparts (Supplementary Table S4), TMB < 6.5 Mb was associated with favorable prognosis (Supplementary Fig. S2a, b, Supplementary Table S5). Dynamic circulating tumor DNA (ctDNA) was analyzed and quantified in 167 plasma samples from 28 patients, and the median number of detections was 5 (2–10). Persistently positive ctDNA in patients who failed to respond from SC (supplementary Fig. S3). We evaluated the role of dynamic plasma Epstein-Barr Virus (EBV)-DNA changes over 1 year and found that EBV-DNA decreased more significantly until undetectable in responsive patients, however, EBV-DNA persistently elevated or unchanged for those failed to respond to SC (Supplementary Fig. S4). Integration of dynamic plasma ctDNA and EBV-DNA showed better prediction of prognosis than ctDNA or EBV-DNA alone (Fig. 5a–f). Univariate analysis demonstrated that genes significantly associated with OS were *STAT3* (*p* = 0.014), *BOCR* (*p* = 0.001), *FAT1* (*p* = 0.001), *EP300* (*p* = 0.02), *KRAS* (*p* = 0.02), *CREBBP* (*p* = 0.01), *NSD1* (*p* = 0.002) and those associated with PFS were *STAT3* (*p* = 0.006), *BCOR* (*p* < 0.001), *PPM1D* (*p* = 0.02), *KRAS* (*p* = 0.02), *CREBBP* (*p* = 0.04), *NSD1* (*p* = 0.003), *GNAS* (*p* = 0.05). At the multivariate analysis, *STAT3* were considered independent prognostic factors of OS and PFS (Supplementary Fig. S2c, d, Supplementary Table S6).

Pretreatment baseline serum T cells lymphocyte subsets and Th1/Th2 cytokines were collected in 27 patients. We observed that patients with higher CD4+/CD8+ ratio (>0.71), CD19 + (>7.8), lower CD3 + CD8 + (≤3.4), and lower plasma interleukin-6 (IL-6) levels (≤2.5 pg/mL), lower plasma interleukin-10 (IL-10) level (≤8.5 pg/mL) had better benefit from SC (Supplementary Figs. S5, 6, Supplementary Tables S7, 8).

### Safety

Treatment related adverse events (TRAEs) are summarized in Table 3. Thirty-eight (100%) patients reported TRAEs. The most frequently observed (≥20%) TRAEs were neutropenia (65.8%), thrombocytopenia (44.7%), anemia (44.7%), hypothyroidism (47.4%), shortened activated partial thromboplastin time (APTT) (39.5%), hypoproteinemia (36.8%), increased transaminase levels (26.3%), nausea and vomiting (26.2%), elevated creatine kinase (26.3%), elevated plasma fibrinogen (23.7%), and elevated hydroxybutyrate dehydrogenase (HBDH) (23.7%). The most frequent grade ≥3 TRAEs (≥10%) were neutropenia (28.9%) and thrombocytopenia (10.5%). Immune-related adverse events (irAEs) were reported in 18 (47.4%) patients, including a grade 4 toxic epidermal necrolysis and an interstitial pneumonia. The most common irAEs were grade 1 hypothyroidism and rash. Prophylactic medication is not recommended in the study and no administration of prophylactic medication was performed. We did not observe an apparent increase in toxicity from prior treatment in SC therapy. Treatment interruptions and dose adjustments are shown in Supplementary Tables S9, 10. TRAEs that led to permanent treatment discontinuation occurred in two (5.3%) patients. No deaths were related to the study drug.

**Table 3:** Adverse events

No. (%)	Any emergent adverse event (n = 38)		Treatment-related AEs (n = 38)	
	Grade 3–4^a^	Any grade	Grade 3–4^a^	Any grade
Neutropenia	15 (39.5)	29 (76.3)	11 (28.9)	25 (65.8)
Leukopenia	5 (13.2)	27 (71.1)	3 (7.9)	25 (65.8)
Lymphopenia	5 (13.2)	23 (60.5)	3 (7.9)	21 (55.3)
Thrombocytopenia	10 (26.3)	21 (55.3)	4 (10.5)	17 (44.7)
Anemia	3 (7.9)	20 (52.6)	0	17 (44.7)
FT3/FT4 elevated	0	18 (47.4)	0	18 (47.4)
Pyrexia	3 (7.9)	15 (39.5)	0	5 (13.2)
TSH elevated	0	16 (42.1)	0	16 (42.1)
APTT shorten	0	15 (39.5)	0	15 (39.5)
Hypoproteinemia	0	14 (36.8)	0	14 (36.8)
Transaminase elevated	2 (5.3)	13 (34.2)	0	10 (26.3)
Plasma fibrinogen elevated	0	13 (34.2)	0	9 (23.7)
Nausea and vomiting	0	10 (26.3)	0	10 (26.3)
Creatine kinase elevated	0	10 (26.3)	0	10 (26.3)
HBDH elevated	0	9 (23.7)	0	9 (23.7)
Hypothyroidism	0	7 (18.4)	0	7 (18.4)
Hyperbilirubinemia	2 (5.3)	6 (15.8)	0	5 (13.2)
Proteinuria	0	6 (15.8)	0	6 (15.8)
Fatigue	0	6 (15.8)	0	6 (15.8)
Hyperglycemia	0	5 (13.2)	0	5 (13.2)
Creatinine elevated	0	5 (13.2)	0	5 (13.2)
Upper respiratory tract infection	0	5 (13.2)	0	5 (13.2)
Hypothyroidism	0	4 (10.5)	0	4 (10.5)
Neuralgia	0	4 (10.5)	0	2 (5.3)
Rash	0	3 (7.9)	0	3 (7.9)
Hematemesis	0	3 (7.9)	0	3 (7.9)
Hypertension	0	3 (7.9)	0	3 (7.9)
Interstitial pneumonia	2 (5.3)	3 (7.9)	2 (5.3)	3 (7.9)
Plasma lipase elevated	0	2 (5.3)	0	2 (5.3)
Herpes zoster	0	2 (5.3)	0	2 (5.3)
Hyperuricemia	0	2 (5.3)	0	2 (5.3)
Generalized edema	1 (2.6)	2 (5.3)	0	2 (5.3)
Mucositis oral	0	2 (5.3)	0	2 (5.3)
Infusion‐related reaction	0	2 (5.3)	0	2 (5.3)
Diarrhea	0	2 (5.3)	0	2 (5.3)
Gastric ulcer	1 (2.6)	2 (5.3)	1 (2.6)	1 (2.6)
Abdominal pain	1 (2.6)	2 (5.3)	1 (2.6)	1 (2.6)
Weight gain	1 (2.6)	2 (5.3)	0	1 (2.6)
Plasma amylase elevated	0	1 (2.6)	0	1 (2.6)
Exfoliative dermatitis	1 (2.6)	1 (2.6)	1 (2.6)	1 (2.6)
Gastritis	0	1 (2.6)	0	1 (2.6)
Blepharoptosis	0	1 (2.6)	0	1 (2.6)
Blurred vision	0	1 (2.6)	0	1 (2.6)
Headache	0	1 (2.6)	0	1 (2.6)

Data are n (%). Grade 1–2 adverse events reported in at least 10% of patients and all grade 3–4 events are shown

^a^Including serious adverse events

## Discussion

To our knowledge, SCENT trial is the first study to provide evidence that epigenetic strategies synergize with anti-PD-1 antibodies to enhance CR rates and a durable response to RR-ENKTL. Sintilimab and chidamide were safely administered with favorable toxicity profiles. Most common irAEs were mild hypothyroidism and rash. However, we observed two cases of SAEs. One patient experienced rare grade 4 exfoliative dermatitis, eventually, this fatal event was resolved with active multimodality therapy. It is unclear whether chidamide enhanced the dermatological toxicity of sintilimab. In the era of chemotherapy, intensified chemotherapy can lead to increased toxicities with subsequent therapies. But we did not observe a significant increase in the incidence of grade 3–4 hematological toxicities and irAEs with SC. Myelosuppression and other toxicities in this trial were manageable with supportive treatment, and no treatment-related death was reported.

SC treatment yielded an ORR of 59.5% with a CR rate of 48.6%. Although phase 2 did not achieve the prespecified target ORR of 67.8% (19/28), SC produced a significantly higher CR rate and markedly extended the DOR and PFS than historic data on single-agent anti-PD-1 or PD-L1 antibodies.^10–12^ These results revealed that epigenetic strategies synergize with anti-PD-1 antibodies to enhance the antitumor effect of immunotherapy in RR-ENKTL. Targeting epigenetic remodeling is a potential therapeutic strategy for developing immunotherapy.^17^ Recently, chidamide was found to exhibit a novel mechanism of action that synergizes with humanized PD-1 antibody to enhance T-cell chemokine expression and augment IFN-γ response in NK-T cell lymphoma compare with romidepsin.^18^ This may be one of the main mechanisms of epigenetic drugs have synergistic antitumor effects with PD-1 monoclonal antibodies in ENKTL.

There are numerous factors that affect the efficacy of immunotherapy. Immune microenvironment is one of the important factors. ENKTL is usually characterized by immunodeficiency and inflammation. Apart from clinical prognostic factors. Various pro-inflammatory cytokines and interleukins (IL)^19–21^ expressed within the tumor microenvironment or circulation and abnormal lymphocyte subsets may also affect patient outcomes. We found efficacy of SC was better in the CD3 + CD8+^low^, CD4+/CD8+^high^, CD19+^high^, IL-6 ^low^, and IL-10 ^low^ group. It may suggest that patients with better autoimmune function benefit more significantly from SC treatment. However, the specific mechanisms are not clear and worth further study in the future.

In the exploratory biomarker assessment, we confirmed that persistent positivity for EBV-DNA or ctDNA was associated with poor survival. The results demonstrated that dynamic plasma EBV-DNA or dynamic ctDNA are critical predictive biomarkers for SC. For the first time, we revealed that combination of dynamic plasma ctDNA and EBV-DNA presents a more accurate prediction of prognosis than ctDNA or EBV-DNA alone. The roles of PD-L1 expression, a traditional biomarker, and TMB in ENKTL remain to be determined. Consistent with the conclusion of solid-tumor cancers, high PD-L1 expression was seemed associated with better outcome. However, unlike the results from most solid-tumor cancers, we found that patients with a lower TMB had significantly longer survival. This contradictory phenomenon may reflect the uniqueness of the immune microenvironment in ENKTL. Consistent with the typical spectrum of mutated genes in ENKTL reported in previous studies, *STAT3* was the most frequent genetic alteration identified. Although our results are not definitive due to the small sample size, we found that *STAT3* mutations in gene expression profiling were strongly associated with a poor prognosis.

Three patients experienced HPD, they all experienced a disastrous prognosis. HPD has no uniform definition in lymphoma, drawing on criteria for solid tumors.^22^ Common clinical features of these HPD cases were high ECOG performance status with uncontrollable high fever, high tumor burden, and plasma EBV-DNA level >10^5^ IU/ml. These patients experienced EBV-DNA load rapid elevated, in addition, dynamic monitoring of EBV-DNA revealed that the elevation of EBV-DNA load appeared several months earlier than radiographic relapsed in 3 patients relapsed after 12 months. We speculate that the EBV replication may cause clinical relapse, which has not been confirmed yet and further studies need to be explored. The incidence of HPD caused by immunotherapy varies greatly across cancer types and reports. In T-cell lymphoma^23,24^ and ENKTL^12^ patients are more likely to develop HPD. The potential mechanism is unclear. Wartewig^25^ found that PD-1 is a haploid sufficient suppressor of T-cell lymphomagenesis in a mouse model. Rauch^26^ revealed a novel connection between the rapid progression of adult T-cell leukemia/lymphoma cells and tumor-resident regulatory T cells. However, identification of the natural process of disease progression or HPD in this special population is challenging. More data is needed, and further study is warranted.

Limitations of this study include its nonrandomized phase 2 design with a relatively small sample size. We didn’t obtain enough tissue and blood samples to perform more in-depth comprehensive bioinformation analysis. Because of this rare virus-associated disease led to enrollment difficulty. We enrolled proportion of patients were treated with anti-PD-1/L1 antibodies or chidamide, this caused did not achieve the prespecified target ORR.

In conclusion, our results showed that treatment with SC had an impressively high CR rate, manageable safety profile and durable response in patients with RR-ENKTL. Thus, this treatment is a promising therapeutic option for this population. A combination of dynamic plasma ctDNA and EBV-DNA played a significant prognostic role. The synergy of epigenetic modulators with anti-PD-1 antibodies can significantly enhance the antitumor efficacy of RR-ENKTL treatment.

## Materials and methods

### Study design and participants

We conducted a multicenter, single-arm, open-label, phase 1b/2 study at 6 cancer centers in China. The eligible patients were 18–75 years of age with histologically confirmed ENKTL per the World Health Organization classification, relapsed or refractory after an asparaginase-based regimen or chemoradiotherapy. These patients had an ECOG performance status of 0–2 and adequate organ function. Patients had at least one measurable lesion according to the International Working Group consensus response evaluation criteria in lymphoma (RECIL 2017).^27^ The key exclusion criteria were as follows: ENKTL-associated hemophagocytic syndrome; central nervous system involvement; invasive natural killer (NK) cell leukemia. The study protocol, describing the full inclusion and exclusion criteria, is available in the supplementary study protocol (Supplementary 2). In August 2019, the study protocol was amended, patients who were previously treated with PD-1/L1 antibodies or chidamide were permitted. All patients provided written informed consent. This study is registered with ClinicalTrials.gov: NCT 03820596.

### Procedures

This was an investigator initiated, open-label, multicenter, phase 1b/2 study. Three periods including screening, treatment, and follow-up. Treatment period consisted of two phases: phase 1b followed by a phase 2 expansion. The standard 3 + 3 design was applied in the dose-escalation phase to identify the MTD, DLTs (definition described in supplementary materials and methods) and RP2D of chidamide in cycle one. In phase 1b, patients received escalating oral doses of chidamide (20, 25, and 30 mg) twice a week along with continuous and fixed doses of 200 mg of sintilimab administered intravenously over a period of 30–60 min once every 21 days. Decisions regarding the risk-benefit ratio of dose escalations and establishment of the RP2D were made by the investigators. In phase 2, patients received fixed doses of 200 mg of sintilimab plus chidamide (RP2D) every 21 days. Patients with CR or PR continued with the combination treatment until disease progression, death, intolerable toxicities, or withdrawal of consent for a maximum of 12 months (18 total cycles). Dose modification, evaluation of efficacy, safety analysis, pre-medications were described in protocol. After 12 months, patients that still experienced CR or PR were allowed to choose whether to continue SC as maintenance or end of treatment or accept ASCT (stem cell mobilization, collection, reinfusion, conditioning regimen prior to ASCT followed the medical routine at each study site). If patients chose to continue SC, the doses of chidamide were reduced to 20 mg twice a week and sintilimab (200 mg) were administered intravenously every 30 days. Patients underwent computed tomography (CT), or whole-body^18^ F-fluorodeoxyglucose (FDG) positron emission tomography (PET) scans before the first treatment and then every 6 weeks up to the sixth cycle and every 12 weeks thereafter. Safety was monitored by laboratory tests, clinical examinations, and chief patient complaints, and all adverse events (AEs), including TRAEs, irAEs, and serious adverse events (SAEs), were recorded. TRAEs were defined as AEs related or possibly related to treatment or for which the relationship to treatment was uncertain, as assessed by the investigators. AEs were defined according to the National Cancer Institute Common Terminology Criteria for Adverse Events (version 5.0).

Pretreatment formalin-fixed, paraffin-embedded (FFPE) tumor samples were obtained from patients. These samples were tested using Dako PD-L1 22C3 (catalog number, SK006; Merck & Co, Inc, Kenilworth, New Jersey) according to the manufacturer’s protocol.^28^ PD-L1 protein expression was determined using the tumor proportion score (TPS) and CPS. TPS and CPS are defined as the percentage of total viable tumor cells showing partial or complete membrane staining of any intensity and the number of PD-L1-stained cells (tumor cells, lymphocytes, and macrophages) divided by the total number of viable tumor cells multiplied by 100, respectively. CPS ≥ 1 was considered positive for PD-L1 expression. Patient blood samples were collected for exploratory dynamics ctDNA and EBV-DNA assessment at baseline and every two cycles. TMB and ctDNA samples were analyzed by capture-based next-generation sequencing (NGS) targeting 475 lymphoma- and cancer-relevant genes (supplementary materials and methods). Baseline peripheral blood samples within 14 days before the SC treatment were collected and performed analysis of T cells lymphocyte subsets and Th1/Th2 cytokines. Lymphocyte subsets were assayed using flow cytometry and percentages of CD3+, CD3 + CD4+, CD3 + CD8 + T, CD3-CD16 + CD56+ Natural Killer cells, and CD19 B-lymphocytes were evaluated by the NAVIOS Instrument (Beckman Coulter, Inc). Plasma cytokines were detected with the human Th1/Th2 Cytokine Kit (IL-2, IL-4, IL-6, IL-10, TNF, and IFN-γ) and samples were run through a FACS Canto II flow cytometer (BD Biosciences).

The primary endpoints were the RP2D of chidamide and the ORR assessed by investigators per RECIL 2017 criteria.^27^ The ORR was defined as the proportion of patients with a CR or PR. Secondary endpoints included PFS, OS, and DOR which was defined as the time from the first CR or PR to recurrence, objectively documented disease progression, or death. Safety and tolerability were additionally evaluated as secondary endpoints.

### Statistical analysis

Simon’s optimal two-stage design was applied in phase 2 to evaluate the efficacy of sintilimab in combination with chidamide.^29^ According to previous trials, the minimum ORR was set as 55%,^15^ and the expected ORR of combination treatment was 80%. Under these assumptions, with 5% (one-sided) type I error and 80% power, 7 patients were enrolled in the first stage. If more than four responses were observed in stage one, an additional 21 patients would be enrolled in stage two, for a total of 28 patients. If more than 19 patients had an ORR among these 28 patients, the combination treatment would be considered promising. Primary analyses of antitumor activity were evaluated in patients at the RP2D (the activity-evaluable population). ORR were reported with two-sided 95% exact confidence intervals (CIs), and the number and percentage of patients in each response category were descriptively tabulated. Survival outcomes, including OS and PFS, were estimated using Kaplan–Meier curves, and group differences were assessed by the log-rank test. The potential factors associated with survival outcomes were analyzed using Cox proportional hazard models, with the proportional hazard assumption confirmed based on the Schoenfeld residuals.^30^ Two-sided statistical tests yielding *P* < 0.05 were considered significant. Analyses of TTR and DOR were conducted with responders only. ROC curves for predicting ORR were used to determine cutoff values for CPS, PD-L1 expression, lymphocyte subsets, and cytokines. Statistical analyses were performed using IBM PASW software version 22.0 (SPSS Inc., Chicago, IL, USA).

### Supplementary information

Supplementary

Protocol

## Data Availability

The raw sequencing data reported in this paper have been deposited in the Genome Sequence Archive in National Genomics Data Center, China National Center for Bioinformation/Beijing Institute of Genomics, Chinese Academy of Sciences (https://ngdc.cncb.ac.cn/gsa-human). These data are accessible under the accession number ‘GSA-Human: HRA006823. These data are under controlled access by human privacy regulations and are only available for research purposes. Access to the data can be granted following approval from the Data Access Committee of the GSA-human database, as detailed at https://ngdc.cncb.ac.cn/gsa-human/document/GSA-Human Request Guide for Users us.pdf. Data are accessible to researchers who meet the criteria for access as defined by the GSA-human database guidelines. Access requests are usually processed within ~4 weeks and data will be available for 3 months once access is granted. Statistical analysis plan, informed consent form, and amendments of the study protocol please contact huanghq@sysucc.org.cn. Individual participant data will not be shared due to restrictions by institution.
