# Supplementary material for: Sintilimab (anti-PD-1 antibody) plus chidamide (histone deacetylase inhibitor) in relapsed or refractory extranodal natural killer T-cell lymphoma (SCENT): a phase Ib/II study
Source: Signal Transduct Target Ther. 2024 May 17;9:121. doi: 10.1038/s41392-024-01825-0 (PMC11099117; doi:10.1038/s41392-024-01825-0)
Supplement: Supplementary file 1 — Supplementary [file 41392_2024_1825_MOESM1_ESM.docx]

**Supplementary Materials for**

**Sintilimab (anti-PD-1 antibody) plus chidamide (histone deacetylase inhibitor) in relapsed or refractory extranodal natural killer T-cell lymphoma (SCENT):**

**a phase Ib/II study**

Yan Gao, Haixia He, Xueping Li, Liling Zhang, Wei Xu, Ru Feng, Wenyu Li, Yin Xiao, Xinxiu Liu, Yu Chen, Xiaoxiao Wang, Bing Bai, Huijing Wu, Qingqing Cai, Zhiming Li, Jibin Li, Suxia Lin, Yanxia He, Liqin Ping, Cheng Huang, Jiaying Mao, Xiujin Chen, Baitian Zhao, Huiqiang Huang

Correspondence to: [Huang-Hui](mailto:huanghq@sysucc.org.cn) Qiang. Department of Medical Oncology, Sun Yat-sen University Cancer Center, 651 Dongfeng Road, east, Guangzhou, China. Phone: +86 020 87343350. Fax: 86-20-87343350. Email: huanghq@sysucc.org.cn

**This PDF file includes:**

Materials and Methods

Fig. S1 to S6

Tables S1 to S10

Table of contents

[1. Supplementary materials and methods 3](#_Toc159081458)

[1.1 Dose-limiting toxicity (DLT) definition 3](#_Toc159081459)

[1.2 Materials and methods for ctDNA detection 3](#_Toc159081460)

[1.3 Methods for TMB detection 7](#_Toc159081461)

[1.4 Methods for PD-L1 expression 7](#_Toc159081462)

[1.5 Methods for EBV load determination 8](#_Toc159081463)

[2. Supplementary Figures 9](#_Toc159081464)

[Figure. S1. Clinical characteristics and the prognosis of OS and PFS 9](#_Toc159081465)

[Figure. S2. TMB, STAT3 mutation and the prognosis of OS and PFS 10](#_Toc159081466)

[Figure. S3. Dynamics of ctDNA during treatment in 12 months (n=28) 11](#_Toc159081467)

[Figure. S4. Dynamics of plasma EBV-DNA during treatment in 12 months(n=28). 12](#_Toc159081468)

[Figure. S5. Efficacy of pretreatment lymphocyte and cytokines (n=27) 13](#_Toc159081469)

[Figure. S6. Survival and pretreatment lymphocyte and cytokines 14](#_Toc159081470)

[3 Supplementary Tables 15](#_Toc159081471)

[Table S1. Summary of prior systemic therapy regimens(n=38) 15](#_Toc159081472)

[Table S2. Efficacy of sintilimab(200mg) plus chidamide (30mg, RP2D) 16](#_Toc159081473)

[Table S3. Univariate and multivariate analysis of clinical characteristics and survival(n=38) 1](#_Toc159081474)

[Table S4. Univariate analyses of prognostic factors for CR/PR with biomarkers(n=28) 15](#_Toc159081475)

[Table S5. Univariate analyses of survival with biomarkers (n=28) 17](#_Toc159081476)

[Table S6. Multivariate analysis of survival with gene mutations*(n=28) 19](#_Toc159081477)

[Table S7. Univariate analyses of efficacy with lymphocyte subsets (n=27) 20](#_Toc159081478)

[Table S8. Univariate analyses of efficacy with cytokines (n=27) 21](#_Toc159081479)

[Table S9. Adverse events from phase 1b(n=9) 22](#_Toc159081480)

[Table S10. Treatment interruption and dose adjustment (n=38) 23](#_Toc159081481)

1. Supplementary materials and methods

## Dose-limiting toxicity (DLT) definition

DLTs, which were assessed during the 21-day observation period of cycle 1, were defined by National Cancer Institute-Common Terminology Criteria for Adverse Events (NCI-CTCAE) and included grade 4 neutropenia (absolute neutrophil count ,500/mm3) lasting more than 7 days, life-threatening grade 3 or higher neutropenia (absolute neutrophil count ,1000/mm3) with fever at least 38.3°C, grade 4 thrombocytopenia (platelets, 25 000/mm3) lasting at least 7 days despite holding treatment, and grade 3 thrombocytopenia with grade 2 or higher bleeding or requiring red blood cell or platelet transfusion. Nonhematologic DLTs were defined as any grade 3 or higher adverse event related to nausea, vomiting, or diarrhea persisting more than 7 days, grade 3 or higher immunotherapy related toxicities, such as pneumonia, thyroid dysfunction, enteritis. Grade 3 infection, fatigue, rash lasting more than 7 days of occurrence by appropriate supportive care.

## Materials and methods for ctDNA detection

1. **Sample preparation.** Peripheral blood samples collected before therapy were used for analyzing mutational profiling. Plasma was separated within 2h of blood collection for ctDNA detection. Written consents from all patients were collected according to the ethic regulations of each site. Collected samples were sent to the core facility of Nanjing Shihe Jiyin Biotechnology Inc. (Nanjing, China) for whole exon sequence analysis.
2. **DNA extraction.** Plasma was used for ctDNA extraction with QIAamp circulating nucleic acid Kit (QIAGEN) following the manufacturer’s instructions. White blood cell was sequenced together with plasma for the purpose of identifying germline mutations. The DNA quality was assessed by Nanodrop2000 (Thermo Fisher Scientific) and the quantity was measured on Qubit 2.0.
3. **Library preparation and sequencing.** Sequencing libraries were prepared with KAPA Hyper Prep kit (KAPA Biosystems) with optimized protocols. In brief, ctDNA were experienced with end-repairing, A-tailing, adapter ligation and size selection using Agencourt AMPure XP beads (Beckman Coulter). Libraries were then subjected to PCR amplification and purification before targeted enrichment. DNA libraries from different samples were marked with unique indices during library preparation and up to 2 μg of different libraries were pooled together for targeted enrichment. Human cot-1 DNA (Life Technologies) and xGen Universal blocking oligos (Integrated DNA Technologies) were added to block nonspecific binding of library DNA to targeted probes. Customized xGen lockdown probes panel (Integrated DNA Technologies) were used to targeted enrich for 475 predefined genes. The hybridization reaction was performed by using NimbleGen SeqCap EZ Hybridization and Wash Kit (Roche). Dynabeads M-270 (Life Technologies) was used to capture probe-bind fragments, followed by library amplification in KAPA HiFi HotStart ReadyMix (KAPA Biosystems), and purification by Agencourt AMPure XP beads. Library quantification was analyzed by KAPA Library Quantification kit (KAPA Biosystems). The size distribution of libraries was measured by Agilent Technologies 2100 Bioanalyzer (Agilent Technologies). The enriched libraries were sequenced on Hiseq 4000 NGS platforms (Illumina) to coverage depths of at 5000╳.
4. **Sequence Data Processing and Identification of Clinically-Actionable Mutations.** Trimmomatic was used for FASTQ file quality control (QC). Leading/trailing low quality (quality reading below 15) or N bases were removed. Reads from each sample were mapped to the reference sequence hg19 (Human Genome version 19) using Burrows-Wheeler Aligner (BWA-mem, v0.7.12) with parameters (-t 8 -M). Local realignment around indels and base quality score recalibration was applied with the Genome Analysis Toolkit (GATK 3.4.0). GATK3.4.0 was applied to detect germline mutations from blood control samples. VarScan2 was employed for detection of somatic mutations (somatic p-value = 0.1, minimum quality score = 15 and otherwise default parameters). Somatic variant calls presenting at less than 1% mutant allelic frequency in the paired blood control sample, but with at least 1% allelic frequency and at least 3 reads supporting variant alleles in tumor samples, were retained. We also filtered mutations reported in dbSNP (v137) and the 1000 Genomes database, but still kept mutations if they were also present in COSMIC database (v76). Annotation was performed using ANNOVAR using the hg19 reference genome and 2014 versions of standard databases and functional prediction programs.
5. **Genomic fusions** were identified by FACTERA with default parameters. In short, we set minimum number of breakpoint-spanning reads to 5, minimum number of discordant reads to 2 and minimum similarity required for alignment of read to fusion template to 95%.
6. **Copy number variations (CNVs)** were detected using ADTEx (http://adtex.sourceforge.net) with default parameters. The main advantage of ADTEx is that it can derive absolute copy numbers without any a priori knowledge of levels of normal DNA contamination or ploidy of the tumor samples. The algorithm takes not only depth of coverage (DOC) ratios but also allele frequency of germline heterozygous SNP (BAF) as inputs. The DOC ratios are smoothed by discrete wavelet transformation techniques prior to applying HMM to estimate polyploidy, normal contamination ratio and absolute CNVs. Germline CNVs from each patient were identified using the blood sample and normal human HapMap DNA sample NA18535 (Coriell Institute) for each captured region (exonic region). Somatic CNVs were identified using paired normal/tumor samples for each exon.

**Custom panel genes analyzed**

| 475 genes |
| --- |
| *ABCB1, ABCC2, ABL1, ABL2, ACTB, ADH1B, AIM1, AIP, AKT1, AKT2, AKT3, ALDH2, ALK, ANKRD26, AP3B1, APC, AR, ARHGAP26, ARID1A, ARID1B, ARID2, ARID5B, ASXL1, ASXL2, ASXL3, ATG5, ATM, ATR, ATRX, AURKA, AURKB, AXIN1, AXL, B2M, BAP1, BARD1, BCL10, BCL11B, BCL2, BCL2L1, BCL2L11, BCL2L2, BCL6, BCL7A, BCOR, BCORL1, BCR, BIRC3, BIRC5, BLM, BMPR1A, BRAF, BRCA1, BRCA2, BRD4, BRIP1, BTG1, BTG2, BTK, BTLA, BUB1B, CALR, CARD11, CBFB, CBL, CBLB, CCND1, CCND2, CCND3, CCNE1, CCR4, CCT6B, CD22, CD274, CD28, CD58, CD70, CD74, CD79A, CD79B, CD83, CDA, CDC73, CDH1, CDK10, CDK12, CDK4, CDK6, CDK8, CDKN1B, CDKN1C, CDKN2A, CDKN2B, CDKN2C, CEBPA, CEP57, CHD8, CHEK1, CHEK2, CIITA, CKS1B, CMTM6, CREBBP, CSF1R, CSF3R, CTCF, CTLA4, CTNNB1, CUX1, CXCR4, CYLD, CYP19A1, CYP2A6, CYP2B6*6, CYP2C19*2, CYP2C9*3, CYP2D6, CYP3A4*4, CYP3A5*3, DAXX, DDR2, DDX3X, DDX41, DHFR, DHX15, DICER1, DNM2, DNMT3A, DNMT3B, DOT1L, DPYD, DTX1, DUSP2, DUSP22, EBF1, ECSIT, ECT2L, EED, EGFR, EGR1, EML4, EP300, EPCAM, EPHA2, EPHA3, ERBB2, ERBB3, ERBB4, ERCC1, ERCC2, ERCC3, ERCC4, ERCC5, ERG, ESR1, ETNK1, ETS1, ETV1, ETV4, ETV6, EWSR1, EZH2, FANCA, FANCC, FANCD2, FANCE, FANCF, FANCG, FANCL, FAS, FAT1, FAT4, FBXO11, FBXW7, FGFR1, FGFR2, FGFR3, FGFR4, FH, FIP1L1, FLCN, FLT1, FLT3, FLT4, FOXO1, FOXO3, FYN, GADD45B, GATA1, GATA2, GATA3, GNA11, GNA13, GNAQ, GNAS, GRIN2A, GSTM1, GSTP1, GSTT1, HACE1, HBA1, HBA2, HBB, HDAC1, HDAC2, HDAC4, HDAC7, HGF, HIST1H1E, HNF1A, HNF1B, HRAS, HSD3B1, ID3, IDH1, IDH2, IGF1R, IKBKE, IKZF1, IKZF2, IKZF3, IL7R, INPP4B, INPP5D, IRF1, IRF4, IRF8, ITPKB, JAK1, JAK2, JAK3, JARID2, JUN, KDM2B, KDM5A, KDM5C, KDM6A, KDR, KIF5B, KIR2DL4, KIR3DL2, KIT, KLF2, KLHL6, KLLN, KLRC1, KLRC2, KLRK1, KMT2A, KMT2B, KMT2C, KMT2D, KRAS, LAMP1, LEF1, LMO1, LMO2, LYN, LYST, MAF, MAFB, MALT1, MAP2K1, MAP2K2, MAP2K4, MAP3K1, MAP3K14, MAP4K3, MAPK1, MCL1, MDM2, MDM4, MED12, MEF2B, MEN1, MET, MFHAS1, MGA, MGMT, MITF, MLH1, MLH3, MPL, MRE11A, MSH2, MSH3, MSH6, MTHFR, MTOR, MUTYH, MYC, MYCL, MYCN, MYD88, MYH11, NAT1, NBN, NCSTN, NF1, NF2, NFKB1, NFKB2, NFKBIA, NFKBIE, NKX2-1, NOTCH1, NOTCH2, NPM1, NQO1, NRAS, NSD1, NT5C2, NTRK1, NTRK3, NUP98, P2RY8, PAG1, PAK3, PALB2, PAX5, PBRM1, PC, PDCD1, PDCD1LG2, PDE11A, PDGFRA, PDGFRB, PDK1, PGR, PHF6, PHOX2B, PIK3CA, PIK3CD, PIK3R1, PIK3R2, PIM1, PLCG2, PML, PMS1, PMS2, POLE, POT1, POU2AF1, PPM1D, PPP2R1A, PRDM1, PRF1, PRKAR1A, PRKCB, PTCH1, PTEN, PTPN1, PTPN11, PTPN13, PTPN2, PTPN6, PTPRD, PTPRK, PTPRO, RAB27A, RAC3, RAD21, RAD50, RAD51, RAF1, RARA, RASGEF1A, RB1, RECQL4, REL, RELN, RET, RHOA, RICTOR, RNF43, ROS1, RPTOR, RRM1, RUNX1, RUNX1T1, RUNX3, SBDS, SDC4, SDHA, SDHB, SDHC, SDHD, SERP2, SETBP1, SETD2, SF3B1, SGK1, SH2B3, SH2D1A, SLC34A2, SLC7A8, SMAD2, SMAD4, SMAD7, SMARCA4, SMARCB1, SMC1A, SMC3, SMO, SOCS1, SOX2, SPEN, SPOP, SRC, SRP72, SRSF2, SRY, STAG2, STAT3, STAT5A, STAT5B, STAT6, STIL, STK11, STMN1, STT3A, STX11, STXBP2, SUFU, SUZ12, SYK, TAL1, TBL1XR1, TBX21, TCF3, TCL1A, TEK, TEKT4, TERT, TET2, TGFBR2, TLE1, TLE4, TMPRSS2, TNFAIP3, TNFRSF11A, TNFRSF14, TNFRSF17, TNFRSF19, TOP1, TOP2A, TP53, TP63, TP73, TPMT, TRAF2, TRAF3, TRAF5, TSC1, TSC2, TSHR, TTF1, TUBB3, TYMS, U2AF1, UGT1A1, UNC13D, VEGFA, VHL, WHSC1, WT1, XIAP, XPC, XPO1, XRCC1, YAP1, ZAP70, ZBTB7A, ZNF2, ZRSR2* |

## Methods for TMB detection

Tumor mutation burden (TMB) was defined as the total number of missense mutations. In addition, we profiled TMB of these samples by a targeted next-generation sequencing (NGS) panel (Geneseeq) to evaluate its correlation with WES results. Panel TMB was counted by summing all base substitutions and indels in the coding region of targeted genes, including synonymous alterations to reduce sampling noise and excluding known driver mutations as they are over-represented in the panel, as previously described.

## Methods for PD-L1 expression

Immunohistochemistry staining was performed using standard procedures. Briefly, The FFPE sections were dewaxed and rehydrated through graded alcohol to water prior to antigen unmasking by heat-induced epitope retrieval and followed by inactivation of endogenous peroxidase with 3% H2O2. Then slides were blocked using a blocking solution and incubated with optimally diluted antibody targeting human PD-L1 overnight at 4 °C. Detection was carried out using Dako REAL HRP Rabbit/Mouse detection kit for 30 min and the signal was subsequently detected by the chromogenic substrate (Dako). Histoscore was a multiplicative index of the intensity of staining and the proportion of positive tumor cells. The intensity was graded as follows: 0, negative staining; 1, mild staining; 2, moderate staining; 3, strong staining. The percentage of stained cells was defined as follows: 1, less than 10%; 2, 10%–50%; 3, 50%–75%; 4, more than 75%. Z-score of IHC is calculated by using the formula z ¼ (x-m) / s, where x is the raw IHC score, m is the population mean, and s is the population SD.

## Methods for EBV load determination

Epstein-Barr virus (EBV) load determination using real-time quantitative polymerase chain reaction. The real-time quantitative polymerase chain reaction (RQ-PCR) has become the method of choice for quantification of specific EBV nucleic acid sequences. This method is fast, extremely sensitive, and accurate, requires only very small amounts of input nucleic acid, and is relatively simple to perform. These characteristics have made it the method of choice for EBV viral load determination.

1. Supplementary Figures

## Fig. S1. Clinical characteristics and the prognosis of OS and PFS


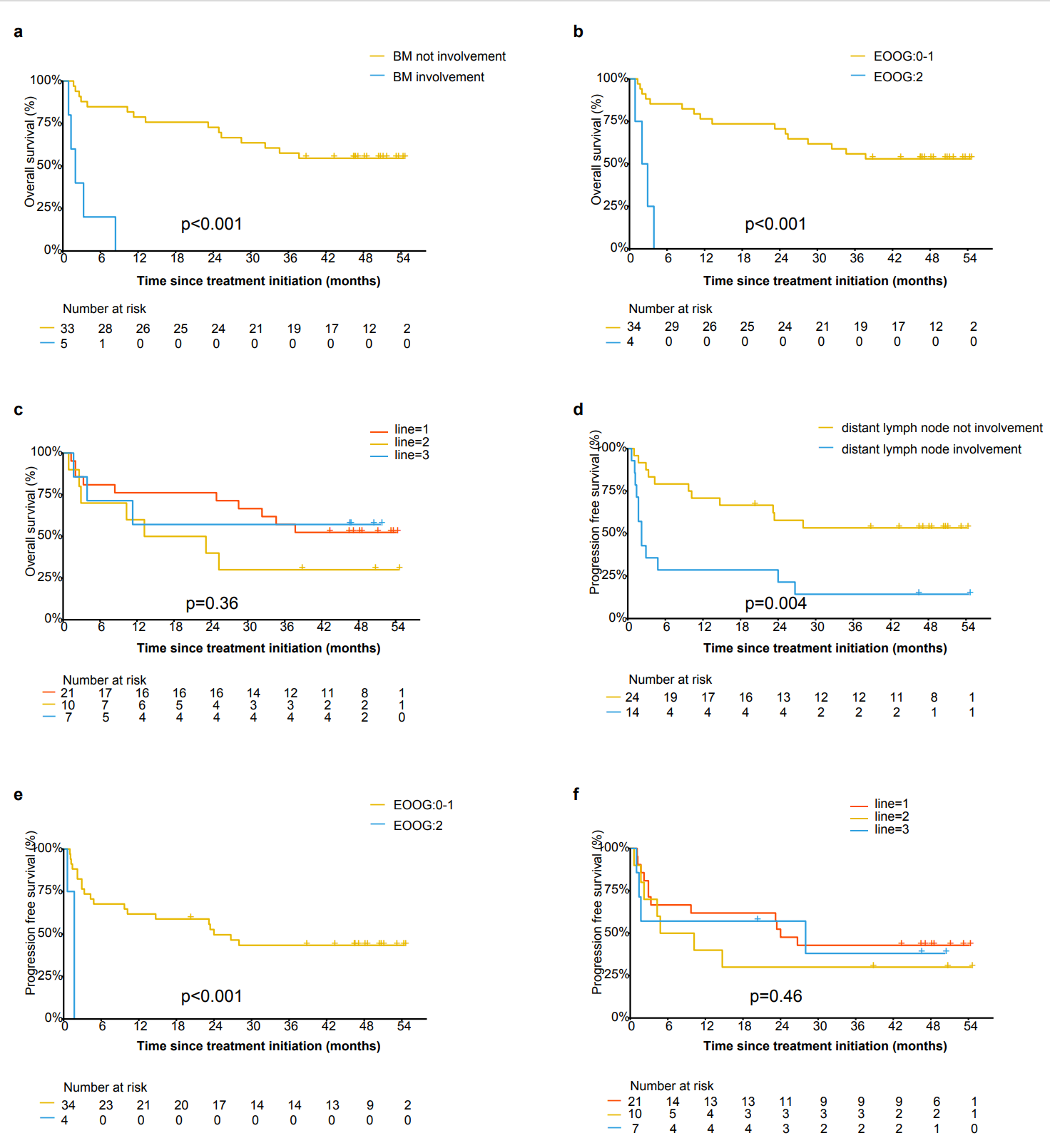


Overall survival according to bone marrow involvement (**a**), ECOG score (**b**), previous systemic therapies lines (**c**). Progression-free survival according to distant lymph node involvement (**d**), ECOG score (**e**) and previous systemic therapies lines (**f**).

## Fig. S2. TMB, STAT3 mutation and the prognosis of OS and PFS


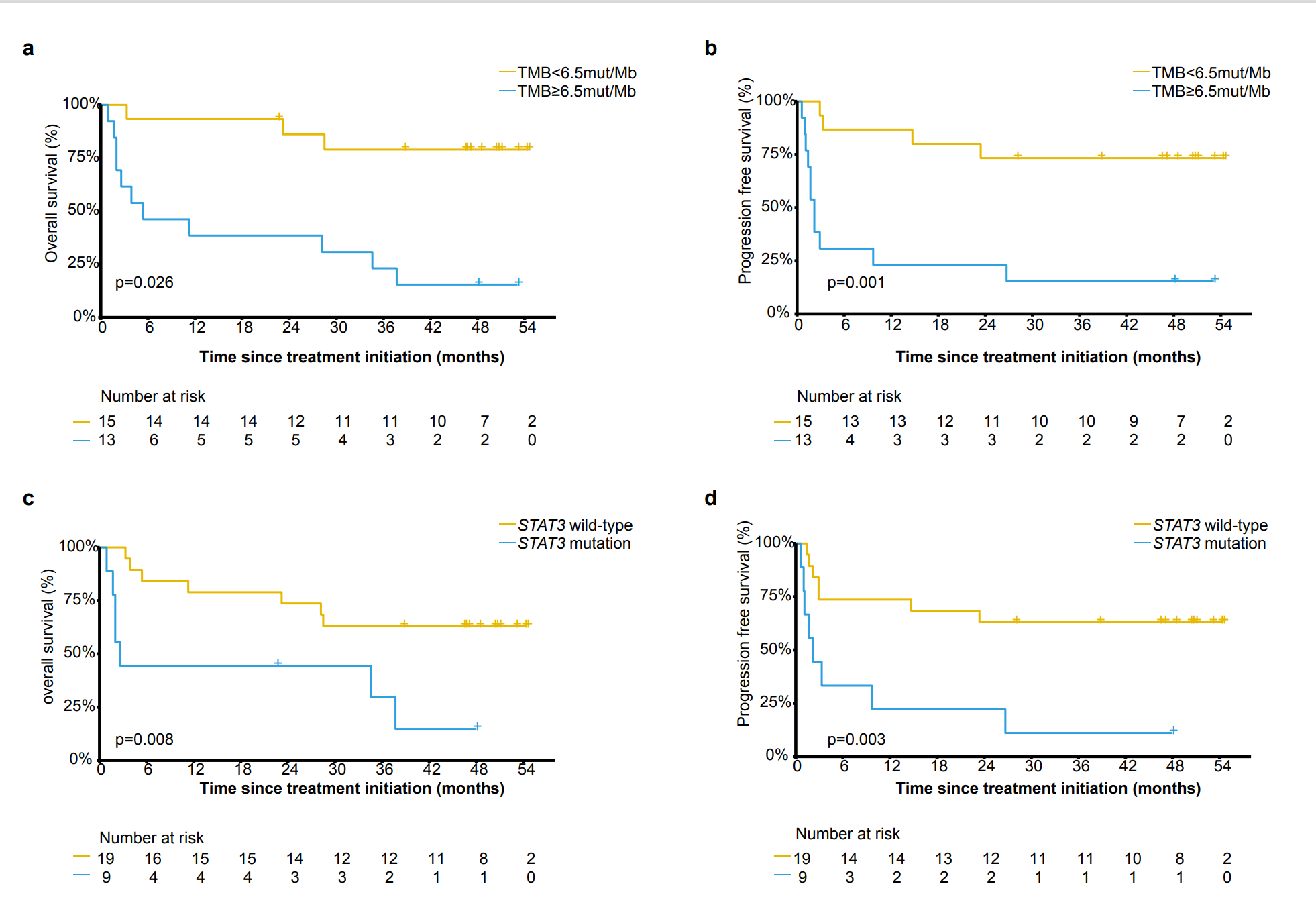


Overall survival (**a, c**) according to TMB and STAT3 mutation. Progression-free survival (**b, d**) according to TMB and STAT3 mutation.

## Fig. S3. Dynamics of ctDNA during treatment in 12 months (n=28)


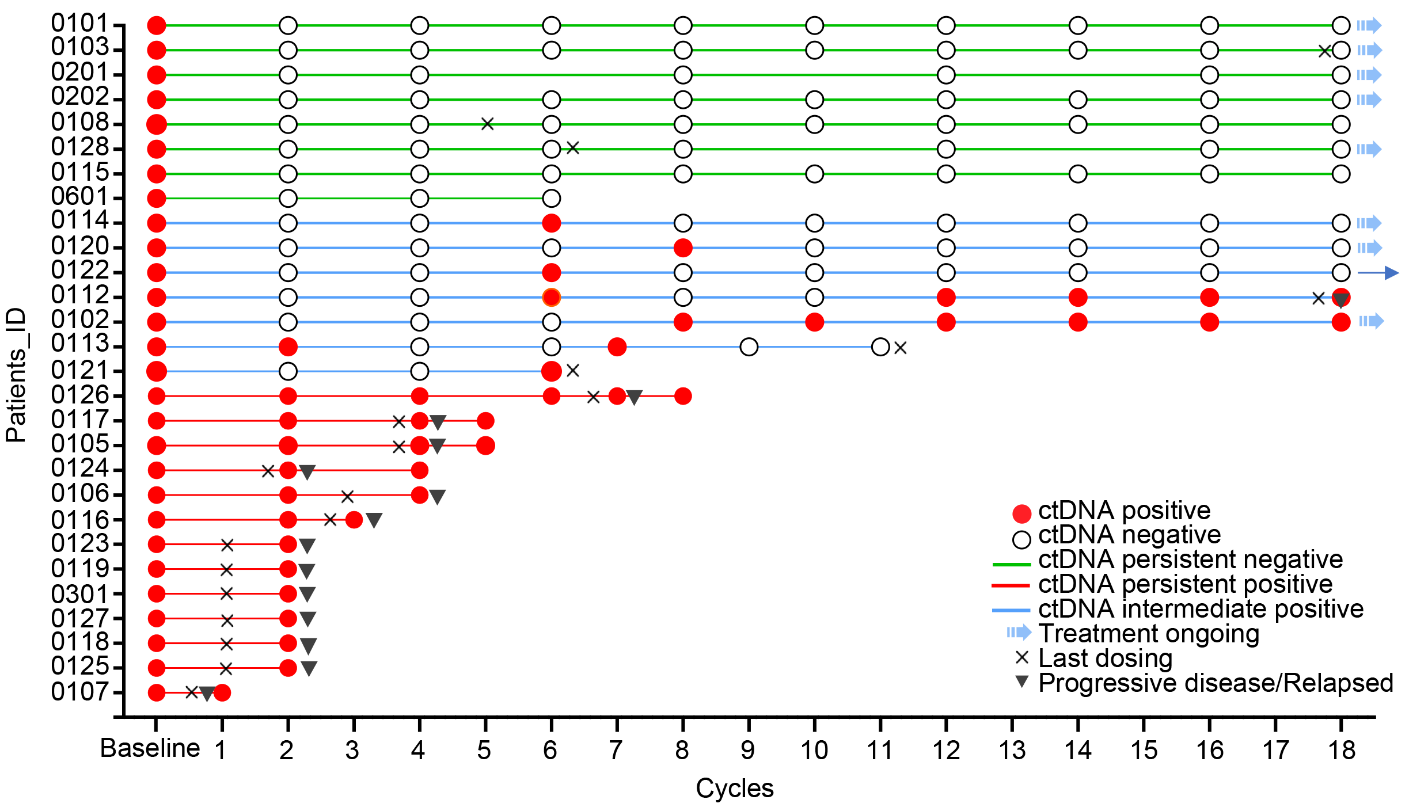


Peripheral blood was collected pretreatment and every two cycles for ctDNA detection. Circles or dots represent individual ctDNA measurements. Response to therapy measured by positron emission tomography/computed tomography (PET/CT) every two cycles during first six cycles.

## Fig. S4. Dynamics of plasma EBV-DNA during treatment in 12 months(n=28).


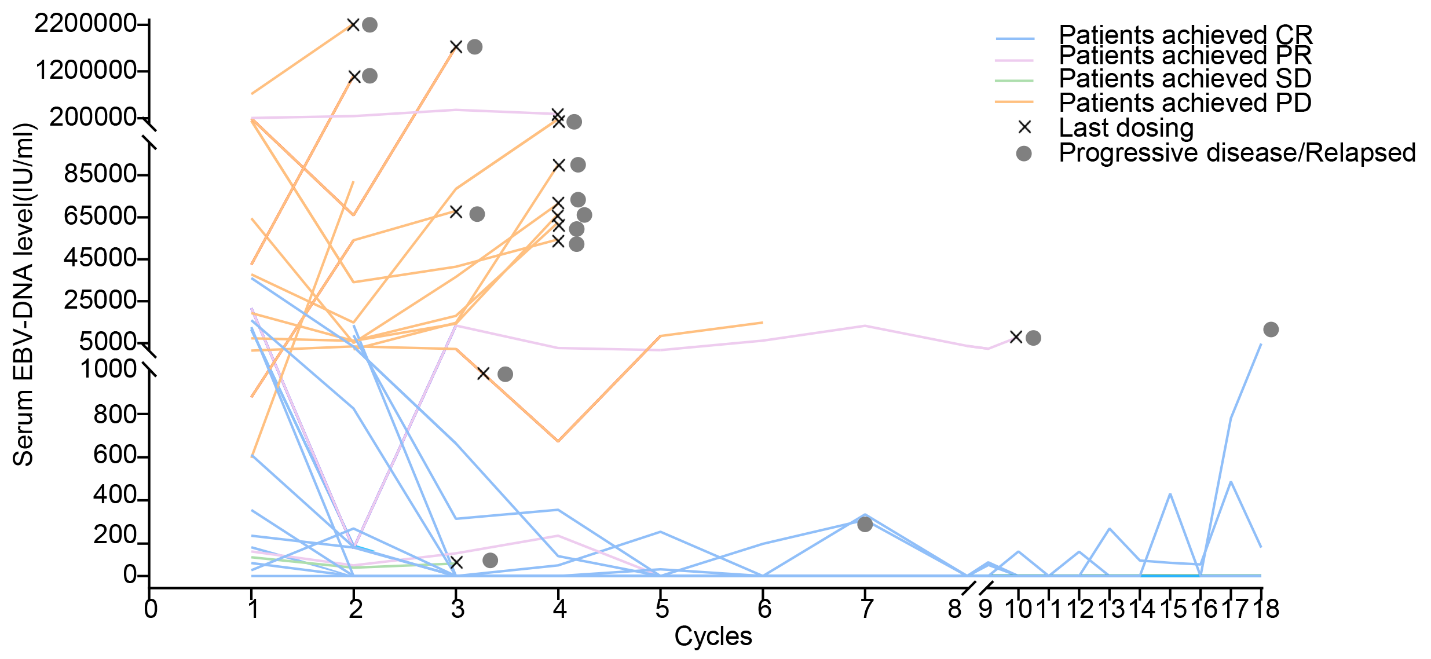


Dots indicate plasma EBV-DNA level before treatment and each cycle. Plasma EBV-DNA were non-detectable(n=3) before and during treatment.

## Fig. S5. Efficacy of pretreatment lymphocyte and cytokines (n=27)


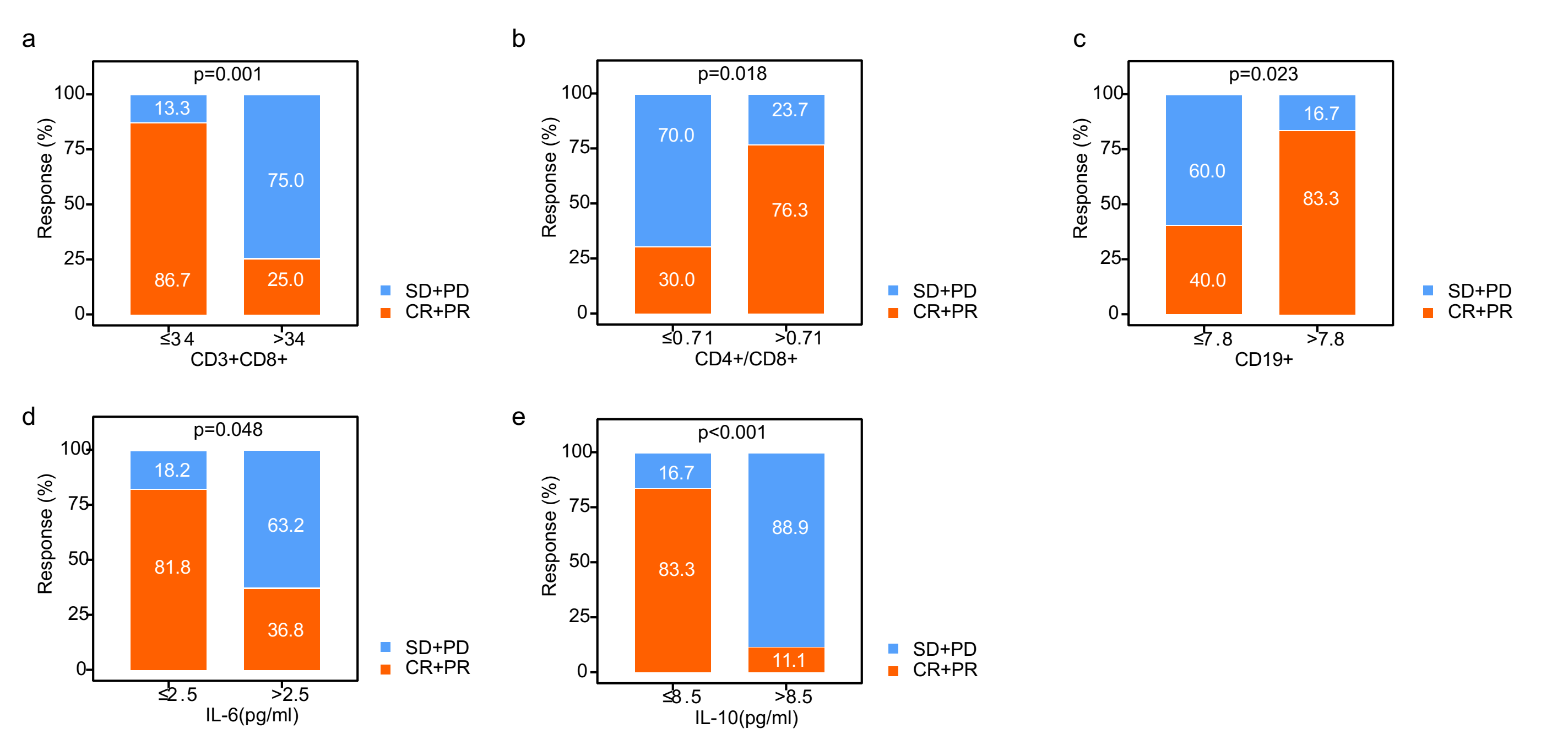


Efficacy of sintilimab plus chidamide was better in the CD3+CD8+^low^ group (**a**), CD4+/CD8+^high^ group (**b**), CD19+^high^ group (**c**), IL-6 ^low^ group (**d**) and IL-10 ^low^ group (**e**).

## Fig. S6. Survival and pretreatment lymphocyte and cytokines


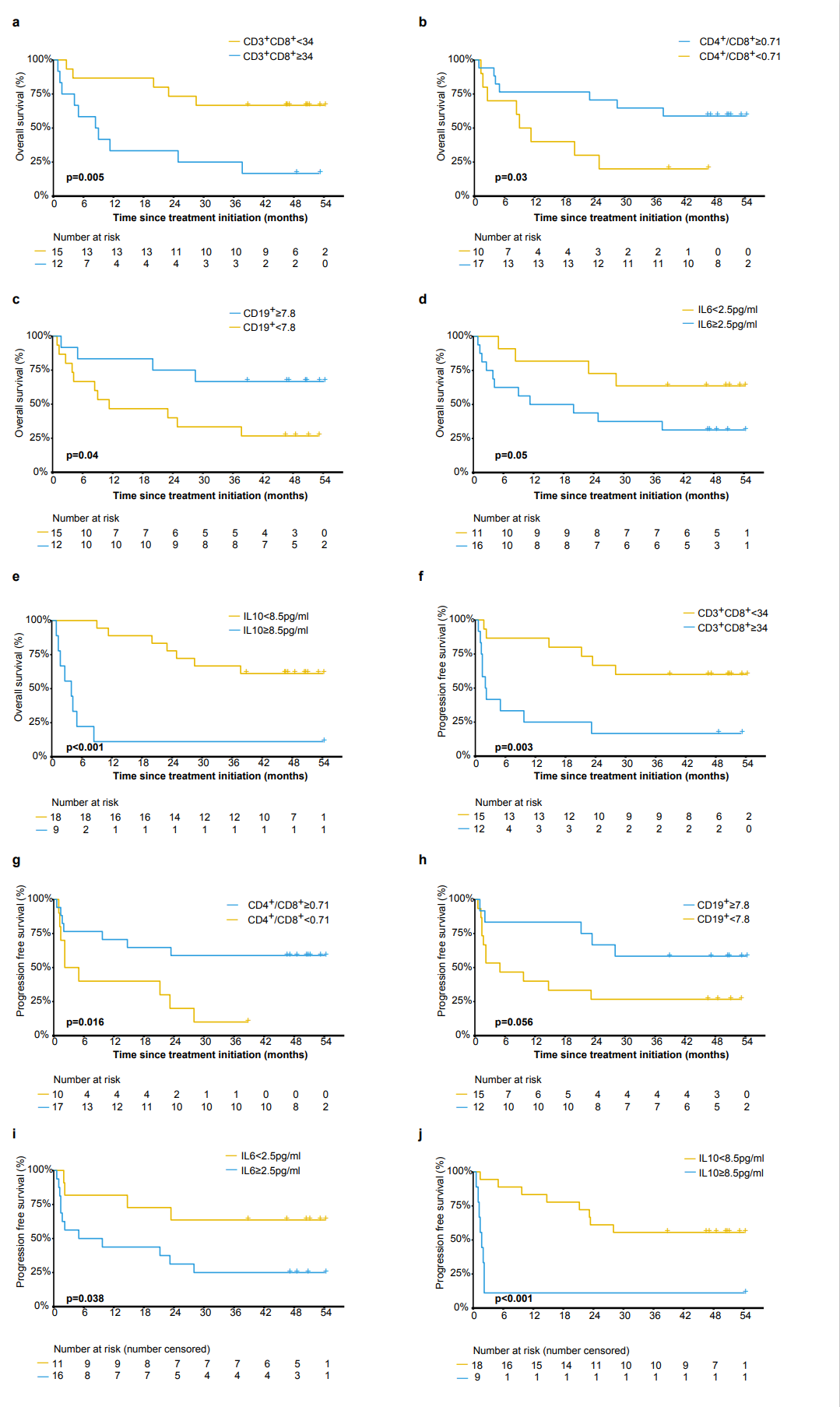


Longer survival was in the CD3+CD8+^low^ group (**a, f**), CD4+/CD8+^high^ (**b, g**), CD19+^high^ (**c, h**), IL-6 ^low^ group (**d, i**) and IL-10 ^low^ group (**e, j**).

# Supplementary Tables

## Table S1. Summary of prior systemic therapy regimens(n=38)

| Prior regimens | Chidamide dose | | | Total（n=38） |
| --- | --- | --- | --- | --- |
|  | 20mg  (n=3) | 25mg  (n=3) | 30mg  (n=32) |  |
| P-GemOx | 3(100%) | 3(100%) | 22(69%) | 28(74%) |
| P-CHOP/E | 0 | 0 | 8(25%) | 7(18%) |
| SMILE | 0 | 0 | 3(9%) | 3(8%) |
| LVP | 0 | 1(33%) | 2(6%) | 3(8%) |
| P-GDP | 0 | 0 | 2(6%) | 2(5%) |
| ICE | 0 | 0 | 2(6%) | 2(5%) |
| HyperCVAD/MA | 0 | 0 | 2(6%) | 2(5%) |
| DEP | 0 | 1(33%) | 1(3%) | 2(5%) |
| Mitoxantrone HL | 0 | 0 | 2(6%) | 2(5%) |
| AspaMetDex | 0 | 0 | 1(3%) | 1(3%) |
| Lenalidomide | 0 | 0 | 1(3%) | 1(3%) |
| Others | 0 | 0 | 2(6%) | 2(5%) |

DEP=liposomal doxorubicin, etoposide, methylprednisolone; P-CHOP/E=pegasparaginase, cyclophosphamide, doxorubicin, vincristine, prednisolone, and/or etoposide; P-Gemox= pegasparaginase, gemcitabine, and oxaloplatin; P-GDP=pegasparaginase, gemcitabine, and cisplatin; HyperCVAD=hyperfractionated combination of cyclophosphamide, doxorubicin, vincristine, and prednisone alternating with methotrexate and cytarabine; LVP=L-asparaginase, vincristine, and prednisolone; Mitoxantrone HL=Mitoxantrone hydrochloride liposome; SMILE=dexamethasone, methotrexate, ifosfamide, pegasparaginase, and etoposide.

## Table S2. Efficacy of sintilimab(200mg) plus chidamide (30mg, RP2D)

|  | Intention-to-treat population  (n=37) | No prior anti-PD-1/L1 antibody or chidamide (n=27) | Prior anti-PD-1/L1 antibody (n=6) | Prior chidamide (n=4) |
| --- | --- | --- | --- | --- |
| Objective response | 22(59%) | 16(59%) | 3(50%) | 3(75%) |
| Complete remission | 18(49%) | 13(48%) | 2(33%) | 3(75%) |
| Partial remission | 4(11%) | 3(11%) | 1(17%) | 0 |
| Stable disease | 2(5%) | 1(4%) | 1(17%) | 0 |
| Progressive disease | 13(35%) | 10(37%) | 2(33%) | 1(25%) |
| Responses were assessed according to the the International Working Group consensus response evaluation criteria in lymphoma (RECIL 2017). | | | | |

PD-L/L1= programmed death –Ligand/ Ligand 1.

## Table S3. Univariate and multivariate analysis of clinical characteristics and survival(n=38)

|  | OS | | | | | | PFS | | | | | |
| --- | --- | --- | --- | --- | --- | --- | --- | --- | --- | --- | --- | --- |
|  | Univariate analysis | | | Multivariate analysis | | | Univariate analysis | | | Multivariate analysis | | |
|  | p value | HR | 95% CI | p value | HR | 95% CI | p value | HR | 95% CI | p value | HR | 95% CI |
| Age(years) | 0.53 | 1.60 | 0.37-6.92 | 0.85 | 0.83 | 0.11-6.08 | 0.30 | 0.46 | 0.11-1.97 | 0.63 | 0.62 | 0.09-4.27 |
| <60 versus≥60 |  |  |  |  |  |  |  |  |  |  |  |  |
| Sex | 0.96 | 1.03 | 0.37-2.82 | 0.95 | 1.04 | 0.27-4.08 | 0.48 | 1.43 | 0.53-3.86 | 0.50 | 1.59 | 0.41-6.14 |
| Male versus Female |  |  |  |  |  |  |  |  |  |  |  |  |
| ECOG | <0.001 | 11.24 | 2.94-43.02 | 0.008 | 3.20 | 1.36-7.54 | 0.001 | 10.20 | 2.55-40.82 | 0.018 | 2.54 | 1.17-5.52 |
| 0-1 versus 2 |  |  |  |  |  |  |  |  |  |  |  |  |
| Ann Arbor stage | 0.16 | 2.07 | 0.75-5.71 | 0.97 | 0.99 | 0.56-1.75 | 0.65 | 1.22 | 0.52-2.89 | 0.80 | 0.94 | 0.56-1.57 |
| I-II versus III-IV |  |  |  |  |  |  |  |  |  |  |  |  |
| Fever at study entry | 0.012 | 3.11 | 1.29-7.53 | 0.31 | 0.44 | 0.09-2.16 | 0.047 | 2.32 | 1.01-5.33 | 0.34 | 0.43 | 0.08-2.38 |
| No versus Yes |  |  |  |  |  |  |  |  |  |  |  |  |
| Serum LDH level at study entry | 0.043 | 2.58 | 1.03-6.49 | 0.11 | 3.52 | 0.76-16.19 | 0.20 | 1.72 | 0.75-3.92 | 0.21 | 2.47 | 0.60-10.25 |
| Normal versus Elevated |  |  |  |  |  |  |  |  |  |  |  |  |
| Lesion’s location | 0.27 | 1.71 | 0.66-4.46 | 0.69 | 1.29 | 0.37-4.50 | 0.18 | 1.84 | 0.75-4.47 | 0.29 | 1.82 | 0.60-5.47 |
| Nasal versus Non-nasal |  |  |  |  |  |  |  |  |  |  |  |  |
| Bone marrow involvement | <0.001 | 13.36 | 3.75-47.59 | 0.032 | 7.35 | 1.19-45.64 | 0.001 | 7.99 | 2.48-25.79 | 0.09 | 4.07 | 0.78-21.33 |
| No versus Yes |  |  |  |  |  |  |  |  |  |  |  |  |
| Distant lymph node involvement | <0.001 | 5.11 | 2.04-12.77 | 0.28 | 4.22 | 1.17-15.18 | 0.004 | 3.39 | 1.48-7.79 | 0.044 | 3.72 | 1.39-13.32 |
| No versus Yes |  |  |  |  |  |  |  |  |  |  |  |  |
| Plasma EBV-DNA level | 0.21 | 2.21 | 0.65-7.54 | 0.70 | 0.68 | 0.92-4.97 | 0.17 | 2.11 | 0.72-6.25 | 0.88 | 0.87 | 0.15-5.00 |
| Non-detectable versus detectable |  |  |  |  |  |  |  |  |  |  |  |  |
| Disease status at study entry | 0.25 | 0.59 | 0.24-1.44 | 0.055 | 3.82 | 0.97-14.94 | 0.15 | 1.85 | 0.79-4.32 | 0.08 | 3.27 | 0.88-12.11 |
| Refractory versus Relapsed |  |  |  |  |  |  |  |  |  |  |  |  |
| Previous systemic therapies | 0.36 | 1.51 | 0.63-3.65 | 0.056 | 4.38 | 1.03-18.7 | 0.46 | 1.36 | 0.60-3.09 | 0.058 | 3.41 | 1.01-11.48 |
| 1 line versus ≥ 2 lines |  |  |  |  |  |  |  |  |  |  |  |  |
| ECOG= Eastern Cooperative Oncology Group, LDH= Lactate Dehydrogenase, EBV= Epstein-Barr virus | | | | | | | | | | | | |

## Table S4. Univariate analyses of prognostic factors for CR/PR with biomarkers(n=28)

|  | CR/PR  (%) | Non-CR/PR  (%) | p value | HR | 95% CI |
| --- | --- | --- | --- | --- | --- |
| PD-L1 expression(n=28) |  |  | <0.001 | 6.96 | 1.84-26.33 |
| 0-24%(n=11) | 2(18.2) | 9(81.8) |  |  |  |
| ≥25%(n=17) | 15(88.2) | 2(11.8) |  |  |  |
| CPS(n=28) |  |  | 0.004 | 6.18 | 1.60-23.87 |
| 0-32(n=10) | 2(20.0) | 8(80) |  |  |  |
| ≥32.5(n=18) | 15(83.3) | 3(16.7) |  |  |  |
| TMB(n=28) |  |  | 0.003 | 0.24 | 0.07-0.86 |
| 0-6 (n=15) | 13(86.7) | 2(13.3) |  |  |  |
| ≥6.5(n=13) | 4(30.8) | 9(59.2) |  |  |  |
| STAT3(n=27) |  |  | 0.07 | 0.49 | 0.22-1.07 |
| Wild type(n=18) | 14(77.8) | 4(12.1) |  |  |  |
| Mutation (9) | 3(33.3) | 6(67.7) |  |  |  |
| BCOR(n=27) |  |  | 0.03 | 0.53 | 0.28-0.99 |
| Wild type(n=21) | 16(76.2) | 5(23.8) |  |  |  |
| Mutation (6) | 1(16.7) | 5(83.3) |  |  |  |
| PPM1D(n=27) |  |  | 1.0 | 0.96 | 0.75-1.22 |
| Wild type(n=25) | 16(60.0) | 9(40.0) |  |  |  |
| Mutation (2) | 1(50.0) | 1(50.0) |  |  |  |
| TP53(n=27) |  |  | 0.08 | 0.7 | 0.47-1.05 |
| Wild type(n=24) | 17(70.8) | 7(29.2) |  |  |  |
| Mutation (3) | 0 | 3(100) |  |  |  |
| DNMT3A(n=27) |  |  | 1.0 | 1.07 | 0.73-1.57 |
| Wild type(n=22) | 13(59.1) | 9(40.9) |  |  |  |
| Mutation (5) | 4(80.0) | 2(20.0) |  |  |  |
| KMT2D(n=27) |  |  | 1.0 | 1.1 | 0.81-1.48 |
| Wild type(n=23) | 14(60.9) | 9(39.1) |  |  |  |
| Mutation (4) | 3(75.0) | 1(25.0) |  |  |  |
| DDX3X(n=27) |  |  | 1.0 | 1.02 | 0.78-1.34 |
| Wild type(n=24) | 15(62.5) | 9(37.5) |  |  |  |
| Mutation (3) | 2(67.7) | 1(33.3) |  |  |  |
| ARID1A(n=27) |  |  | 0.27 | 1.31 | 1.00-1.70 |
| Wild type(n=23) | 13(56.5) | 10(43.5) |  |  |  |
| Mutation (4) | 4(100) | 0 |  |  |  |
| CHEK2(n=27) |  |  | 1.0 | 1.02 | 0.78-1.34 |
| Wild type(n=24) | 15(62.5) | 9(37.5) |  |  |  |
| Mutation (3) | 2(67.7) | 1(33.3) |  |  |  |
| MGA (n=27) |  |  |  |  |  |
| Wild type(n=25) | 16(60.0) | 9(40.0) | 1.0 | 0.96 | 0.75-1.22 |
| Mutation (2) | 1(50.0) | 1(50.0) |  |  |  |
| FAT1(n=27) |  |  |  |  |  |
| Wild type(n=24) | 17(70.8) | 7(29.2) | 0.08 | 0.7 | 0.47-1.05 |
| Mutation (3) | 0 | 3(100) |  |  |  |
| EP300(n=27) |  |  | 1.0 | 0.96 | 0.75-1.22 |
| Wild type(n=25) | 16(60.0) | 9(40.0) |  |  |  |
| Mutation (2) | 1(50.0) | 1(50.0) |  |  |  |
| KRAS(n=27) |  |  | 0.25 | 0.80 | 0.59-1.09 |
| Wild type(n=25) | 17(68.0) | 8(32.0) |  |  |  |
| Mutation (2) | 0 | 2(100) |  |  |  |
| DOT1L (n=27) |  |  | 1.0 | 1.02 | 0.78-1.34 |
| Wild type(n=24) | 15(62.5) | 9(37.5) |  |  |  |
| Mutation (3) | 2(67.7) | 1(33.3) |  |  |  |
| NFKB1 (n=27) |  |  | 1.0 | 0.96 | 0.75-1.22 |
| Wild type(n=25) | 16(60.0) | 9(40.0) |  |  |  |
| Mutation (2) | 1(50.0) | 1(50.0) |  |  |  |
| CREBBP (n=27) |  |  | 1.0 | 0.96 | 0.75-1.22 |
| Wild type(n=25) | 16(60.0) | 9(40.0) |  |  |  |
| Mutation (2) | 1(50.0) | 1(50.0) |  |  |  |
| NSD1 (n=27) |  |  | 0.25 | 0.80 | 0.59-1.09 |
| Wild type(n=25) | 17(68.0) | 8(32.0) |  |  |  |
| Mutation (2) | 0 | 2(100) |  |  |  |
| CIITA (n=27) |  |  | 1.0 | 0.96 | 0.75-1.22 |
| Wild type(n=25) | 16(60.0) | 9(40.0) |  |  |  |
| Mutation (2) | 1(50.0) | 1(50.0) |  |  |  |
| FAT4 (n=27) |  |  | 0.25 | 0.80 | 0.59-1.09 |
| Wild type(n=25) | 17(68.0) | 8(32.0) |  |  |  |
| Mutation (2) | 0 | 2(100) |  |  |  |
| SRC (n=27) |  |  | 0.71 | 1.13 | 0.95-1.35 |
| Wild type(n=25) | 15(60.0) | 10(40.0) |  |  |  |
| Mutation (2) | 2(100) | 0 |  |  |  |
| EGFR (n=27) |  |  | 0.25 | 0.80 | 0.59-1.09 |
| Wild type(n=25) | 17(68.0) | 8(32.0) |  |  |  |
| Mutation (2) | 0 | 2(100) |  |  |  |
| GNAS (n=27) |  |  | 1.0 | 0.96 | 0.75-1.22 |
| Wild type(n=25) | 16(60.0) | 9(40.0) |  |  |  |
| Mutation (2) | 1(50.0) | 1(50.0) |  |  |  |

## Table S5. Univariate analyses of survival with biomarkers (n=28)

|  | OS | | | PFS | | |
| --- | --- | --- | --- | --- | --- | --- |
|  | p value | HR | 95% CI | p value | HR | 95% CI |
| PD-L1 expression(n=28) | 0.22 | 1.96 | 0.67-5.78 | 0.09 | 2.41 | 0.86-6.75 |
| 0-25%(n=8) |  |  |  |  |  |  |
| >25%(n=20) |  |  |  |  |  |  |
| CPS(n=28) | 0.43 | 1.53 | 0.53-4.48 | 0.23 | 1.87 | 0.67-5.21 |
| 0-32.5(n=10) |  |  |  |  |  |  |
| >32.5(n=18) |  |  |  |  |  |  |
| TMB(n=28) | 0.002 | 0.13 | 0.04-0.49 | 0.002 | 0.152 | 0.05-0.49 |
| 0-6.5(n=15) |  |  |  |  |  |  |
| >6.5(n=13) |  |  |  |  |  |  |
| STAT3(n=28) | 0.014 | 3.79 | 1.31-10.93 | 0.006 | 4.23 | 1.51-11.83 |
| Wild type(n=19) |  |  |  |  |  |  |
| Mutation(n=9) |  |  |  |  |  |  |
| BCOR(n=28) | 0.001 | 6.92 | 2.25-21.25 | <0.001 | 7.59 | 2.60-22.17 |
| Wild type(n=22) |  |  |  |  |  |  |
| Mutation(n=6) |  |  |  |  |  |  |
| PPM1D(n=28) | 0.11 | 3.65 | 0.75-17.66 | 0.02 | 7.43 | 1.34-41.22 |
| Wild type(n=26) |  |  |  |  |  |  |
| Mutation (2) |  |  |  |  |  |  |
| TP53 (n=28) | 0.09 | 3.08 | 0.85-11.20 | 0.11 | 2.83 | 0.78-10.23 |
| Wild type(n=24) |  |  |  |  |  |  |
| Mutation(n=4) |  |  |  |  |  |  |
| DNMT3A(n=28) | 0.80 | 1.21 | 0.27-5.42 | 0.65 | 0.71 | 0.16-3.13 |
| Wild type(n=23) |  |  |  |  |  |  |
| Mutation(n=5) |  |  |  |  |  |  |
| KMT2D(n=28) | 0.37 | 1.80 | 0.50-6.54 | 0.56 | 1.47 | 0.41-5.26 |
| Wild type(n=24) |  |  |  |  |  |  |
| Mutation(n=4) |  |  |  |  |  |  |
| DDX3X(n=28) | 0.21 | 2.28 | 0.63-8.22 | 0.32 | 1.90 | 0.53-6.73 |
| Wild type(n=24) |  |  |  |  |  |  |
| Mutation(n=4) |  |  |  |  |  |  |
| ARID1A(n=28) | 0.78 | 0.81 | 0.18-3.63 | 0.79 | 0.82 | 0.18-3.64 |
| Wild type(n=24) |  |  |  |  |  |  |
| Mutation(n=4) |  |  |  |  |  |  |
| CHEK2 (n=28) | 0.07 | 3.49 | 0.93-13.13 | 0.14 | 2.63 | 0.73-9.49 |
| Wild type(n=25) |  |  |  |  |  |  |
| Mutation(n=3) |  |  |  |  |  |  |
| MGA (n=27) | 0.06 | 4.86 | 0.97-24.34 | 0.10 | 3.77 | 0.76-18.70 |
| Wild type(n=25) |  |  |  |  |  |  |
| Mutation (2) |  |  |  |  |  |  |
| FAT1(n=28) | 0.001 | 50.37 | 5.04-503.8 | 0.93 | >10^7^ | 0->10^7^ |
| Wild type(n=25) |  |  |  |  |  |  |
| Mutation(3) |  |  |  |  |  |  |
| EP300(n=28) | 0.02 | 7.44 | 1.34-41.21 | 0.06 | 4.46 | 0.94-21.17 |
| Wild type(n=26) |  |  |  |  |  |  |
| Mutation (2) |  |  |  |  |  |  |
| KRAS(n=28) | 0.02 | 7.08 | 1.42-35.33 | 0.02 | 7.13 | 1.42-35.57 |
| Wild type(n=26) |  |  |  |  |  |  |
| Mutation (2) |  |  |  |  |  |  |
| DOT1L (n=28) | 0.23 | 2.51 | 0.56-11.29 | 0.32 | 2.14 | 0.48-9.54 |
| Wild type(n=25) |  |  |  |  |  |  |
| Mutation (3) |  |  |  |  |  |  |
| NFKB1 (n=28) | 0.07 | 4.45 | 0.89-22.21 | 0.15 | 3.10 | 0.65-14.65 |
| Wild type(n=26) |  |  |  |  |  |  |
| Mutation (2) |  |  |  |  |  |  |
| CREBBP (n=28) | 0.01 | 10.81 | 1.96-59.69 | 0.04 | 5.21 | 1.10-24.64 |
| Wild type(n=26) |  |  |  |  |  |  |
| Mutation (2) |  |  |  |  |  |  |
| NSD1 (n=28) | 0.002 | 21.49 | 2.97-155.7 | 0.003 | 41.48 | 3.67-468.9 |
| Wild type(n=26) |  |  |  |  |  |  |
| Mutation (2) |  |  |  |  |  |  |
| CIITA (n=28) | 0.86 | 1.20 | 0.16-9.22 | 0.98 | 1.03 | 0.14-7.84 |
| Wild type(n=26) |  |  |  |  |  |  |
| Mutation (2) |  |  |  |  |  |  |
| FAT4 (n=28) | 0.94 | 0.93 | 0.12-7.12 | 0.32 | 2.16 | 0.47-9.88 |
| Wild type(n=26) |  |  |  |  |  |  |
| Mutation (2) |  |  |  |  |  |  |
| SRC (n=28) | 0.31 | 2.19 | 0.48-9.91 | 0.24 | 2.44 | 0.55-10.88 |
| Wild type(n=26) |  |  |  |  |  |  |
| Mutation (2) |  |  |  |  |  |  |
| EGFR (n=28) | 0.17 | 2.96 | 0.64-13.77 | 0.11 | 3.57 | 0.75-16.94 |
| Wild type(n=26) |  |  |  |  |  |  |
| Mutation (2) |  |  |  |  |  |  |
| GNAS (n=28) | 0.10 | 9.51 | 1.71-52.83 | 0.05 | 4.81 | 1.01-22.84 |
| Wild type(n=26) |  |  |  |  |  |  |
| Mutation (2) |  |  |  |  |  |  |

## Table S6. Multivariate analysis of survival with gene mutations*(n=28)

|  | OS | | | PFS | | |
| --- | --- | --- | --- | --- | --- | --- |
|  | p value | HR | 95% CI | p value | HR | 95% CI |
| STAT3 | 0.024 | 28.10 | 1.55-510.73 | 0.009 | 33.25 | 2.37-466.17 |
| WT versus MT |  |  |  |  |  |  |
| BCOR | 0.71 | 1.557 | 0.15-15.84 | 0.33 | 3.11 | 0.32-30.49 |
| WT versus MT |  |  |  |  |  |  |
| TP53 | 0.16 | 7.01 | 0.46-107.57 | 0.06 | 17.67 | 0.92-339.72 |
| WT versus MT |  |  |  |  |  |  |
| DNMT3A | 0.11 | 6.48 | 0.67-63.21 | 0.07 | 6.91 | 0.86-55.51 |
| WT versus MT |  |  |  |  |  |  |
| KMT2D | 0.008 | 52.35 | 2.75-997.40 | 0.06 | 28.71 | 0.90-374.91 |
| WT versus MT |  |  |  |  |  |  |
| DDX3X | 0.61 | 3.21 | 0.04-296.15 | 0.18 | 16.57 | 0.28-985.72 |
| WT versus MT |  |  |  |  |  |  |
| ARID1A | 0.66 | 2.96 | 0.02-389.63 | 0.61 | 2.97 | 0.05-195.72 |
| WT versus MT |  |  |  |  |  |  |
| CHEK2 | 0.025 | 30.14 | 1.88-484.09 | 0.06 | 10.81 | 0.95-122.56 |
| WT versus MT |  |  |  |  |  |  |
| FAT1 | 0.016 | 76.65 | 1.74-3385.29 | 0.93 | 526425 | 0-2.247E+141 |
| WT versus MT |  |  |  |  |  |  |
| DOT1L | 0.08 | 15.89 | 0.72-351.27 | 0.12 | 9.29 | 0.56-154.58 |
| WT versus MT |  |  |  |  |  |  |

WT means wild type, MT means mutation.

* Multivariate analysis was performed for gene mutation rates > 10%.

## Table S7. Univariate analyses of efficacy with lymphocyte subsets (n=27)

| Lymphocyte subsets | CR/PR  n(%) | SD/PD  n(%) | P value |
| --- | --- | --- | --- |
| CD4+/CD8+ |  |  | 0.018 |
| ≤0.71(n=10) | 3(30.0) | 7(70.0) |  |
| >0.71(n=17) | 13(76.5) | 4(23.5) |  |
| CD3+ |  |  | 0.32 |
| ≤86.5(n=21) | 14(66.7) | 7(33.3) |  |
| >86.5(n=6) | 2(33.3) | 4(66.7) |  |
| CD3+CD4+ |  |  | 0.29 |
| ≤23.5(n=8) | 3(37.5) | 5(62.5) |  |
| >23.5(n=19) | 13(68.4) | 6(31.6) |  |
| CD3+CD8+ |  |  | 0.001 |
| ≤34(n=15) | 13(86.7) | 2(13.3) |  |
| >34(n=12) | 3(25.0) | 9(75.0) |  |
| CD3-CD16+CD56+ |  |  | 0.053 |
| ≤27.5(n=21) | 15(71.4) | 6(28.6) |  |
| >27.5(n=6) | 1(16.7) | 5(83.3) |  |
| CD4+CD25+ |  |  | 0.43 |
| ≤14.5(n=16) | 8(50.0) | 8(50.0) |  |
| >14.5(n=11) | 8(72.7) | 3(27.3) |  |
| CD8+CD25+ |  |  | 0.58 |
| ≤12(n=14) | 9(64.3) | 5(35.7) |  |
| >12(13) | 7(53.8) | 6(46.2) |  |
| CD19+ |  |  | 0.023 |
| ≤7.8(n=15) | 6(40.0) | 9(60.0) |  |
| >7.8(12) | 10(83.3) | 2(16.7) |  |

## Table S8. Univariate analyses of efficacy with cytokines (n=27)

| Cytokine(pg/ml) | CR/PR n(%) | SD/PD n(%) | P value |
| --- | --- | --- | --- |
| IL-2 |  |  | 1.0 |
| ≤2.5(n=26) | 16(61.5) | 10(38.5) |  |
| >2.5(n=1) | 0 | 1(100) |  |
| IL-4* |  |  | - |
| ≤2.5(n=27) | 16(59.3) | 11(40.7) |  |
| >2.5(n=0) | 0 | 0 |  |
| IL-6 |  |  | 0.048 |
| ≤2.5(n=11) | 9(81.8) | 2(18.2) |  |
| >2.5 (n=19) | 7(36.8) | 9(63.2) |  |
| IL-10 |  |  | <0.001 |
| ≤8.5(n=18) | 15(83.3) | 3(16.7) |  |
| >8.5 (n=9) | 1(11.1) | 8(88.9) |  |
| TNF* |  |  | - |
| ≤2.5(n=27) | 16(59.3) | 11(40.7) |  |
| >2.5(n=0) | 0 | 0 |  |
| IFN-γ |  |  | 0.053 |
| ≤12.5(n=21) | 15(71.4) | 6(28.6) |  |
| >12.5(n=6) | 1(16.7) | 5(83.3) |  |

*Decreased degrees of freedom due to constants or linear dependent covariates

## Table S9. Adverse events from phase 1b(n=9)

|  | Any emergent adverse event  (n=9) | | Treatment-related AEs  (n=9) | |
| --- | --- | --- | --- | --- |
|  | Grade 3-4 | Any grade | Grade 3-4^a^ | Any grade |
| Neutropenia | 2(22.2) ^a^ | 6(66.7) | 2(22.2) | 6(66.7) |
| Leukopenia | 2(22.2) ^a^ | 6(66.7) | 2(22.2) | 6(66.7) |
| Lymphopenia | 0 | 3(33.3) | 0 | 3(33.3) |
| Thrombocytopenia | 0 | 2(22.2) | 0 | 2(22.2) |
| Anemia | 0 | 2(22.2) | 0 | 2(22.2) |
| FT3/FT4 elevated | 0 | 3(33.3) | 0 | 3(33.3) |
| Pyrexia | 0 | 3(33.3) | 0 | 1(11.1) |
| TSH elevated | 0 | 2(22.2) | 0 | 2(22.2) |
| APTT shorten | 0 | 1(11.1) | 0 | 1(11.1) |
| Hypoproteinemia | 0 | 2(22.2) | 0 | 2(22.2) |
| Transaminase elevated | 0 | 2(22.2) | 0 | 2(22.2) |
| Nausea and vomiting | 0 | 2(22.2) | 0 | 2(22.2) |
| Creatine kinase elevated | 0 | 1(11.1) | 0 | 1(11.1) |
| Hyperbilirubinemia | 0 | 1(11.1) | 0 | 1(11.1) |
| Hypothyroidism | 0 | 1(11.1) | 0 | 1(11.1) |
| Generalized edema | 0 | 1(11.1) | 0 | 1(11.1) |
| Diarrhea | 0 | 1(11.1) | 0 | 1(11.1) |
| Blepharoptosis | 0 | 1(3) | 0 | 1(3) |
| Note.  Data are n (%). Grade 1–2 adverse events reported in at least 10% of patients and all grade 3–4 events are shown.  ^a^ Recovered within 7 days and no fever developed. | | | | |

## Table S10. Treatment interruption and dose adjustment (n=38)

|  | Treatment interruption(n=23,%) | Dose adjustment(n=4) |
| --- | --- | --- |
| Disease progression | 17(74) | 0 |
| Active withdraw from treatment | 4(17) | 0 |
| Exfoliative dermatitis(Grade 4) | 1(4) | 0 |
| Interstitial pneumonia(Grade 4) | 1(4) | 0 |
| Neutropenia (Grade 4) | 0 | 2(50) |
| Thrombocytopenia(Grade 3) | 0 | 2(50) |
