# Supplementary material for: Sintilimab (anti-PD-1 antibody) plus chidamide (histone deacetylase inhibitor) in relapsed or refractory extranodal natural killer T-cell lymphoma (SCENT): a phase Ib/II study
Source: Signal Transduct Target Ther. 2024 May 17;9:121. doi: 10.1038/s41392-024-01825-0 (PMC11099117; doi:10.1038/s41392-024-01825-0)
Supplement: Supplementary file 2 — Protocol [file 41392_2024_1825_MOESM2_ESM.pdf]

CLINICAL STUDY PROTOCOL

Sintilimab plus chidamide in patients with relapsed or refractory extranodal natural killer T-cell lymphoma (SCENT): an open-label, multicenter, single-arm, phase 1b/2 trial

PROTOCOL NO.: SCENT

**Sponsor**	**Sun Yat-sen University Cancer Center**
	651 Dongfeng Road East, Yuexiu District
	Guangzhou, P.R. China
**Sponsor Contact**	**Huiqiang Huang, MD, PhD**
	Deputy director of department of Medical Oncology, Sun Yat-sen University Cancer Center
	Phone: 86-020-87343350
	E-mail:huanghq@sysucc.org.cn
**Date of Original Protocol**	14 July 2018
**Protocol Amendment 1**	20 January 2019
**Protocol Amendment 2**	08 August 2019

Confidentiality Statement

The concepts and information contained in this document or generated during the study are considered proprietary and may not be disclosed in whole or in part without the express written consent of Sun Yat-sen University Cancer Center

Declaration of Investigator

I have read and understood all sections of the protocol entitled: “Efficacy and Safety of Sintilimab plus Chidamide in patients with relapsed or refractory Extranodal natural killer T cell lymphoma: an open-label, multicenter, single-arm, phase 1b/2 trial”.

I will not supply the study drug to any person not authorized to receive it. Confidentiality will be protected. Patient identity will not be disclosed to third parties or appear in any study reports or publications.

I will not disclose information regarding this clinical investigation or publish results of the investigation without authorization from Sun Yat-sen University Cancer Center.

Signature of Principal Investigator Date

DOCUMENT HISTORY

Document	Date of Issue	Overall Rationale
**Amendment 02**	08-August-2019	Modifying the inclusion criteria, patients who were previously treated with PD-1/L1 antibodies or chidamide were permitted.
**Amendment 01**	20-January-2019	Modifying several of the inclusion criteria to better define the appropriate participant population.
**Original Protocol**	14-July-2018	Not applicable

PROTOCOL AMENDMENT SUMMARY OF CHANGES

Amendment: 01

Overall Rationale for the Amendments:

Detailed description of inclusion and exclusion criteria, and expanded indications.

Summary of Changes Table:

Section # and Name (Before modification)	Description of Change	Brief Rationale
4.1 Inclusion criteria<br>2. Male or female, age over 18 years (including 18) old	2. Male or female, age over 18 years (including 18) old and no more than 75 years old.	Extremely low incidence rate in patients over 80 years, and lack of efficacy and safety data in this population.
4.1 Inclusion criteria<br>9. a) Absolute neutrophil count ≥ 1.5 × 10^9 /L for subjects without marrow involvement of lymphoma;<br>b) Hemoglobin ≥ 90 g/ L (without red blood cell infusion within 14 days);<br>c) Platelet count ≥ 75 × 10^9 / L for subjects without marrow involvement of lymphoma;	4.1 Inclusion criteria<br>9. a) Absolute neutrophil count ≥ 1.5 × 10^9 /L for subjects without marrow involvement of lymphoma; absolute neutrophil count ≥ 1.0 × 10^9 / L for subjects with marrow involvement;<br>b) Hemoglobin ≥ 90 g/ L (without red blood cell infusion within 14 days), hemoglobin ≥ 75 g/ L for subjects without marrow involvement of lymphoma;<br>c) Platelet count ≥ 75 × 10^9 / L for subjects without marrow involvement of lymphoma; platelet count ≥ 50 × 10^9 / L for subjects with marrow involvement;	Give a detailed description of hematological function for patients with marrow involvement.
4.2 Exclusion Criteria:<br>2. Hemophagocytic syndrome;	2. Active hemophagocytic syndrome (Suffered from hemophagocytic syndrome previously but recovered more than 6 months could enrolled);	Expanded inclusion criteria

Amendment: 02

Overall Rationale for the Amendments:

Modifying the inclusion criteria, patients who were previously treated with PD-1/L1 antibodies or chidamide were permitted.

Summary of Changes Table:

**Section # and Name (Before modification)**	**Description of Change**	**Brief Rationale**
4.1 Inclusion criteria<br>4. Available tumor tissue samples (10-15 unstained, fresh-frozen, paraffin-embedded [FFPE] slides) obtained from past or fresh coarse-needle puncture or excision.	~~4. Available tumor tissue samples (10-15 unstained, fresh-frozen, paraffin-embedded [FFPE] slides) obtained from past or fresh coarse-needle puncture or excision.~~	Tissue specimens not available for some patients
4.2 Exclusion Criteria:<br>4. Received prior therapy with anti-PD-1/L1 antibodies or chidamide;	4. ~~Received prior therapy with anti-PD-1/L1 antibodies or chidamide;~~	Due to slow enrollment progress, patients who were previously treated with PD-1/L1 antibodies or chidamide were permitted.

Table of Contents

TITLE PAGE
Protocol Synopsis....10
Schedule of assessments....17
1. Introduction and Background....20
1.1 Overview of Extranodal NK/T Cell Lymphoma....20
1.2 Description of Investigational Study Drug....22
2. Study Objectives and Endpoints....24
3. Study Design....26
3.1 Overall Study design....26
3.2 Screening Period....28
3.3 Treatment Period....28
3.4 End of Treatment....29
3.5 Follow-up Period....29
3.6 End of Study....29
4. Patient selection....29
4.1 Inclusion criteria....29
4.2 Exclusion Criteria:....32
4.3 Screening Failures:....34
5 Treatment....35
5.1 Dosage and Treatment cycle of Study drug....35
5.2. Packaging and storage of sintilimab....36
5.3 Toxicities of sintilimab....38
5.4 Premedication of sintilimab....38
5.5 Dose adjustment....38
5.6 Management of sintilimab –related infusion reactions....40
5.7 Toxicities of chidamide....42
5.8 Premedication of chidamide....43
5.9 Dose adjustment....43
5.10 Combined medication and non-drug treatment....44
5.12 Criteria for discontinuation of research drugs or exit of this trial....45
5.13 Criteria for discontinuation of study drugs....46
5.14 Treatment compliance....46

6 Study progress....47
6.1 Screening period (Day -28 to Day 0) 47
6.2 Visits during the treatment period 49
6.3 End-of-treatment visit.... 51
6.4 Follow-up of progression-free survival 52
6.5 Follow-up of overall survival 52
7 Response assessments....52
7.1 Outcome measures 52
7.2 Evaluation of efficacy.... 53
8 Safety assessment....54
8.1 Safety Endpoint 54
8.2 Evaluation of Adverse Events 55
8.3 Safety assessment of laboratory and other inspections.... 61
8.4 Suitability of measurement.... 62
9 Data management....62
10 Statistics and statistical analysis....62
10.1 Calculation of sample size.... 63
10.2 Analysis of the population.... 64
10.3 Safety analysis.... 66
10.4 Efficacy analysis.... 67
11 Study management....67
11.1 Ethical considerations.... 67
11.2 Informed Consent 68
11.3 Compensation for patient's health damage 69
11.4 Quality control and quality assurance 69
11.5 Modification of the study protocol 69
11.6Termination of the study.... 69
11.7 Summary report of the research 70
11.8 Confidentiality and publication of research results 70
12 Appendices....70
12.1 Appendix 1. Eastern Cooperative Oncology Group Performance Status.... 70
12.2 Appendix 2. RECIL 2017:Response categories based on assessment of target lesions.... 71

List of Abbreviations

Abbreviation	Definition
18FDG/PET	18F-Fluorodeoxyglucose/Positron Emission Tomography
AEs	Adverse events
APTT	Activated Partial Thromboplastin Time
ALT	Alanine Aminotransferase
ASCT	Autologous hematopoietic stem cell transplantation
AST	Aspartate Aminotransferase
CIs	Confidence intervals
CR	Complete remission
CT	Computed Tomography
CTCAE	Common Terminology Criteria for Adverse Events
CTL	Cytotoxic T cells
CRF	Case Report Form
ctDNA	Circulating tumor Deoxyribo Nucleic Acid
DLTs	Dose limited toxicities
DNMTi	DNA methyltransferases
DoR	Duration of Response
DNA	Deoxyribo Nucleic Acid
EBV	Epstein-Barr virus
ECG	Electrocardiogram
ECOG	Eastern Cooperative Oncology Group
EMT	Epithelial-mesenchymal phenotypic transformation
ENKTL	Extranodal natural killer/T cell lymphoma
EOT	End of treatment
FDG	Fluorodeoxyglucose
FEV1	Forced expiratory volume
FFPE	Fresh-frozen, paraffin-embedded
FT3	Free thyroxine3
FT4	Free thyroxine4
FVC	Forced vital capacity
GCP	Good clinical practice
HBsAg	Hepatitis B Surface Antigen
HCV	Hepatitis C Virus
HDACi	Histone deacetylase inhibitor
HIV	Human immunodeficiency virus
ICF	Informed Consent Form
IEC	Independent Ethics Committee
IRB	Institutional Review Board
INR	International Normalized Ratio
MedDRA	Medical Dictionary for Regulatory Activities
MTD	Maximum tolerated dose
MRI	Magnetic resonance imaging

MRR	Minor Remission Rate
NCCN	National Comprehensive Cancer Network
NHL	Non-Hodgkin's lymphoma
NMPA	National Medical Products Administration
ORR	Objective response rate
OS	Overall Survival
PD	Progression disease
PD1	programmed death 1
PDL1	programmed death ligand 1
PFS	Progression-Free Survival
PFT	pulmonary function test
PR	Partial response
PT	Prothrombin Time
PTCL	Peripheral T cell lymphoma
RECIL	Response Criteria for Malignant Lymphoma
RP2D	Recommended Phase II dosage
RR	relapsed/refractory
SAEs	Serious adverse events
TN	Treatment naïve
WOBCP	Women of Childbearing Potential
TSH	Thyroid-stimulating hormone
ULN	Upper Limit Normal

Protocol Synopsis

**Protocol ID**	SCENT
**Title**	Sintilimab plus chidamide in patients with relapsed or refractory extranodal natural killer T-cell lymphoma (SCENT): an open-label, multicenter, single-arm, phase 1b/2 trial
**Study Phase**	Phase 1b/2
**Indication**	Relapsed or refractory Extranodal natural killer T cell lymphoma
**Interventional Study Model**	Open-label, multicenter, single-arm
**Rationale**	Extranodal natural killer/T cell lymphoma (ENKTL) is a distinct lymphoid neoplasm with aggressive course and poor outcomes. Optimal treatment strategies for relapsed or refractory disease are controversial. In small retrospective studies have observed promising response and survival rates in patients treated with L-asparaginase. In several prospective study that examined relapsed/refractory (RR) patients treated with SMILE outside a trial setting, the efficacy sounds good. But treatment related mortality was 7%. The regimen has toxicity, with careful attention to adverse effects and skill acquired through experience. Therefore, the development of novel effective therapies or strategies for this population is urgently. ENKTL are invariably infected by Epstein-Barr virus (EBV). EBV-infected lymphoma cells upregulate programmed death ligand 1 (PDL1), ligand of the inhibitory receptor programmed death 1(PD1) on T cells. Ligation of PDL1 on lymphoma cells with PD1 on effector T cells suppresses T-cell cytotoxicity. The PDL1/PD1 axis is therefore a potential mechanism for ENKTL to avert effector T-cell targeting. PD1 blockade with pembrolizumab was a potent strategy for ENKTL failing L-asparaginase regimens in several reports. However, the complete remission (CR) rate reaches 30% merely and the remission time is short. Therefore, it is necessary to explore new combinatorial therapeutics with immunotherapy in RR-ENKTL. Chidamide, an oral subtype-selective histone deacetylase inhibitor (HDACi) monotherapy was effective on the patients with RR-ENKTL in our study. Objective response rate (ORR) was 50.0% (6/12) with CR rate 33.3% (4/12). All four CR patients were still in disease-free more than 6.9 months (6.9-10.5). Recent studies on the role of epigenetics in immune evasion have exposed a key role for epigenetic modulators in augmenting the tumor microenvironment and restoring immune recognition and immunogenicity. Theoretically, anti-PD-1 antibody combination with epigenetics agents can improve the curative effect. Thus, we carried out a single, open-label, multicenter clinical trial enrolled patients with RR-ENKTL to safety and efficacy of sintilimab in combination with chidamide.
**Objectives**	**Primary Objective**

	• Evaluate the efficacy of sintilimab plus chidamide in patients with RR-ENKTL.<br>• Identify the maximum tolerated dose (MTD), dose limited toxicities (DLTs) and recommended Phase II dosage (RP2D) of chidamide in combination with sintilimab.
	**Secondary Objectives**<br>• Long-term survival of sintilimab plus chidamide in patients with RR-ENKTL.<br>• The safety profile of sintilimab plus chidamide.
	**Exploratory Objectives**<br>Explore correlations between clinical activity and tumor and/or blood biomarkers.
**Endpoints**	**Primary Endpoint**<br>Overall response rate (ORR) is defined as the proportion of patients with a best overall response of complete response (CR) or partial response (PR) according to the RECIL 2017 Response Criteria for Malignant Lymphoma as determined by investigators.<br>**Secondary Endpoints**<br>• Progression-Free Survival (PFS) is defined as the time from the treatment date to the date of recurrence, disease progression or death.<br>• Duration of Response (DoR) is defined as the date of their first objective response (which is subsequently confirmed) to disease progression or among participants who experience an objective response.<br>• Overall Survival (OS) is defined as the time from treatment to the date of death.<br>• Frequency and severity of adverse events (AEs) and serious adverse events (SAEs)
**Study Design**	This is a Phase 1b/2, multi-center, open-label, single-arm study. The study will enroll approximately 37-40 patients.
	In phase 2, Simon's two-stage design was used, in the first stage, 7 patients were to be accrued, if the ORR rate was less than 4/7, the study would be stopped. If not, patient recruitment would continue until the number reached 28, however, further enrollment will be halted if futility is confirmed.
**Screening criteria**	**Inclusion Criteria:**<br>1. Subjects who have fully understood the study and have signed the informed consent form (ICF).<br>2. Male or female, age over 18 years (including 18) old and no more than 75 years old.<br>3. Extranodal NK/T-cell lymphoma confirmed by histopathology examination.<br>4. Refractory and relapsed ENKTCL that has failed the treatment of asparaginase-based chemotherapy or radiochemotherapy.<br>Definition of refractory:

	i. Failure to get PR after chemotherapy with asparaginase-containing regimen; or<br>ii. Disease progression within 6 months of the final asparaginase-containing regimen.<br>5. Subjects with Eastern Cooperative Oncology Group (ECOG) performance status score of 0 or 1.<br>6. The expected survival time is over 3 months.<br>7. There must be at least 1 evaluable or measurable lesion that meets the RECIL 2017 lymphoma criteria [evaluable lesion: 18F-Fluorodeoxyglucose/Positron Emission Tomography (18F-Fluorodeoxyglucose/Positron Emission Tomography, 18FDG/PET) examination showing increased lymph nodes or extranodal uptake (higher than liver) and PET and/or Computed Tomography (CT) features are consistent with lymphoma manifestations; measurable lesions: nodular lesions longer than 15mm or extranodal lesions longer than 10mm (if the only measurable lesion has received radiotherapy in the past, there must be evidence of radiological progression after radiotherapy) and accompanied by increased 18FDG uptake]. Except for this, there is no measurable increase in the diffuse 18FDG uptake in the liver.<br>8. Subjects should have adequate hematological, hepatic, and renal function as defined by the following laboratory examinations. Subjects have not received cell growth factors, platelet or granulocyte infusion within 7 days before hematological examinations (for the long-acting granulocyte-colony stimulating factor [G-CSF] and [PEG-CSF], the interval of 2 weeks is required):<br>i. Absolute neutrophil count ≥ 1.5 × 10^9 / L for subjects without marrow involvement of lymphoma; absolute neutrophil count ≥ 1.0 × 109 / L for subjects with marrow involvement;<br>ii. Hemoglobin ≥ 90 g/ L (without red blood cell infusion within 14 days), hemoglobin ≥ 75 g/ L for subjects without marrow involvement of lymphoma;<br>iii. Platelet count ≥ 75 × 10^9 / L for subjects without marrow involvement of lymphoma; platelet count ≥ 50 × 109 / L for subjects with marrow involvement;<br>iv. Upper Limit Normal (ULN), or creatinine clearance (≥ 40 mL/min) of serum creatinine (≤ 1.5 times normal value upper limit) (estimated by the Cockcroft-Gault formula);<br>v. Serum total bilirubin ≤ 1.5 times ULN;<br>vi. Aspartate Aminotransferase (AST), Alanine Aminotransferase (ALT) ≤2.5 times ULN;<br>vii. Coagulation function: International Normalized Ratio (INR) ≤ 1.5 times ULN; Prothrombin Time (PT), Activated Partial Thromboplastin Time (APTT) ≤ 1.5 times ULN (unless the

	subject is receiving anticoagulant therapy and PT and APTT are using anticoagulant therapy at the screening time).<br>viii. Thyroid-stimulating hormone (TSH) or free thyroxine (FT4) or free triiodothyronine (FT3) are all within the normal range (±10%).<br>9. There was no evidence that subjects had difficulty breathing at rest, and the measured value of pulse oximetry at rest was more than 92%;<br>10. Subjects must pass a pulmonary function test (PFT) to confirm that forced expiratory volume (FEV1)/forced vital capacity (FVC) in the first second is more than 60%, unless it is a large mediastinal mass caused by lymphoma that cannot meet this standard; carbon monoxide diffusion (DLCO), FEV1 and FVC are all above 50% of the predicted value; all PFT results must be obtained within four weeks before the first administration;<br>11. Subjects who have received anti-tumor therapy should be screened only after the toxicity of the previous treatment has recovered to the grade 1 level or baseline level according to Common Terminology Criteria for Adverse Events (CTCAE) V5.0;<br>12. Women of Childbearing Potential (WOBCP) must undergo a serum pregnancy test within seven days before the first medication and the results are negative. WOBCP or men and their WOBCP partners should agree to take effective contraceptive measures from the signing of ICF until six months after the last dose of the research drug is used.<br>**Exclusion Criteria:**<br>1. Invasive natural killer cell leukemia;<br>2. Active hemophagocytic syndrome (Suffered from hemophagocytic syndrome previously but recovered more than 6 months could enrolled.);<br>3. Primary central nervous system lymphoma or secondary central nervous system involvement;<br>4. Received organ transplantation in the past;<br>5. Patients who received allogeneic hematopoietic stem cell transplantation within three years;<br>6. Adverse reactions from the previous anti-tumor treatment have not yet recovered to ≤Grade 1 based on NCI-CTCAE [Version 5.0], except for hair loss and pigmentation;<br>7. Autologous hematopoietic stem cell transplantation was performed within 90 days before the start of the study;<br>8. Patients with active autoimmune diseases requiring systematic treatment in the past two years (hormone replacement therapy is not considered systematic treatment, such as type I diabetes mellitus, hypothyroidism requiring only thyroxine replacement therapy, adrenocortical dysfunction or pituitary dysfunction requiring only physiological doses of glucocorticoid replacement therapy); Patients

	with autoimmune diseases who do not require systematic treatment within two years can be enrolled;<br>9. Subjects requiring systemic glucocorticoid therapy or other immunosuppressive therapy for a given condition within 14 days before treatment [allowing subjects to use local, ocular, intra-articular, intranasal and inhaled glucocorticoid therapy (with very low systemic absorption); and allowing short-term (< 7 days) glucocorticoid prophylaxis (e.g., contrast agent overdose) Sensitivity] or for the treatment of non-autoimmune diseases (e.g. delayed hypersensitivity caused by contact allergens);<br>10. In the past five years, patients with other malignant tumors have undergone radical treatment, except for basal cell carcinoma of skin, squamous cell carcinoma of skin, carcinoma in situ of breast and carcinoma in situ of cervix;<br>11. Subjects have major surgery within four weeks prior to enrollment or those who have not completely recovered from any previous invasive operation;<br>12. Uncontrolled hypertension (the systolic pressure > 180 mmHg and/or diastolic pressure >100 mmHg after treatment);<br>13. Active hemorrhagic diseases;<br>14. Begin research on live vaccination (except influenza attenuated vaccine) within 28 days before treatment;<br>15. Human immunodeficiency virus (HIV) infection (HIV positive);<br>16. Patients with active hepatitis B or active hepatitis C. Patients who are positive for hepatitis B Surface Antigen (HBsAg) or hepatitis C Virus (HCV) antibodies at screening stage must pass further detection of hepatitis B Virus (HBV) DNA titer (no more than 1000 copies/mL or 500 IU/mL) and HCV RNA (no more than the lower limit of the detection method) in the row. In addition to active hepatitis B or hepatitis C infections requiring treatment, group trials can be conducted. Hepatitis B carriers, stable hepatitis B (DNA titer should not be higher than 2500 copies/mL or 500 IU/mL) after drug treatment, and cured hepatitis C patients can be enrolled in the group;<br>17. Patients with active pulmonary tuberculosis;<br>18. Known history of hypersensitivity to a similar drug and any excipient in the study drug;<br>19. Pregnant or lactating women;<br>20. Have uncontrollable complications, including but not limited to symptomatic congestive heart failure, uncontrollable hypertension, unstable angina, active peptic ulcer or hemorrhagic diseases;<br>21. Any severe or uncontrolled systematic disease, systematic complication, other severe complicated diseases, or any special condition that might make the subjects unsuitable for the study, or might affect the compliance to the protocol or might cause significant

	interference in safety/ efficacy evaluation of the study drug as judged by the investigator.
**Established treatment and study duration**	The duration of the study subjects is defined as the time from the date of first dosage of sintilimab plus chidamide to the completion of the follow-up period, withdrawal of consent, loss to follow-up, or death, whichever occurs first.<br>The study will include a screening period (of up to 28 days), a treatment period (cycles of 3 weeks, up to 1 year), and a follow-up period (approximately every 12 weeks visits for up to 3 years after treatment discontinuation).<br>Treatment after 1 year, patients can continue sintilimab plus chidamide, or autologous hematopoietic stem cell transplantation (ASCT) as consolidation, or stop treatment and observation.<br>The end of study occurs at the last visit or last scheduled procedure for the last patient, unless the study is terminated earlier by investigators.
**Efficacy assessments:**	Positron emission tomography – computed tomography (PET-CT) was highly recommended. If disease is not PET-avid at baseline, CT or magnetic resonance imaging (MRI) may be used for follow-up disease assessments.
**Safety assessments:**	1. Physical examination<br>2. Vital signs<br>3. Change of clinical symptom<br>4. Safety laboratories (hematology, biochemistry, EBV-DNA, reatine kinase isoenzyme, Free thyroxine)<br>5. 12 Lead-ECG (triplicate)<br>6. Pregnancy test, if applicable<br>7. AEs/SAEs, graded according to CTCAE version 5.0
**Biomarkers' assessments:**	Blood sampling for dynamic ctDNA;<br>Tumor tissue for biomarkers (e.g., immunohistochemistry of PD-L1 expression);
**Study Drug, Dosage, and Administration:**	In the phase Ib dose-escalation portion, patients received oral escalating doses of chidamide (20, 25, 30 mg) twice a week continuous and fixed doses of 200mg sintilimab intravenously over a period of 30–60 min, once every 21 days.<br>In the phase II portion, patients received fixed doses of 200mg sintilimab plus chidamide (RP2D) every 21 days, for a maximum of 12 months.
**Sample Size:**	Approximately 37-40 patients.
**Statistical analysis**	**Study Hypotheses:**<br>• ORR based on investigators for patients treated with sintilimab plus chidamide to be 80% with a one-sided type 1 error rate of 5% and at least 80% power.<br>• Simon's two-stage design was used.<br>• Sample size for phase II of the study was calculated assuming the target ORR of combination treatment to be 80% with a one-sided type 1 error

	rate of 5% and at least 80% power.<br>• ORRs were reported with two-sided 95% exact confidence intervals (CIs), and the number and percentage of patients in each response category were descriptively tabulated. DOR, PFS, RFS, and OS will be analyzed by Kaplan-Meier approach. Safety analyses will be presented descriptively.

Schedule of assessments

Study flow chart

**Study period**	**Screening**	**Treatment period**						**Follow-up period (up to 3 years from EOT)**		
	**Screening**	1	2	3	4	5-N	EOT[1]	Safety follow up [2]	Radiography follow up [3]	Survival follow up[4]
Visit time and window period	D−28 to −1	±3d	±3d	±3d	±3d	±3d	+28d	30 days after the last treatment ± 3days		12 weeks after the last treatment ± 7 days
Study management process										
Informed consent	×									
Eligibility criteria	×									
Demography	×									
Medical/Cancer history [5]	×	×	×	×	×	×	×	×		
Clinical operation / evaluation										
Adverse event [6]	×	×	×	×	×	×	×	×		
12-lead ECG [7]	×	×	×	×	×	×	×	×		
Height, Weight, Vital signs (BP, HR, RR, Temp) [8]	×	×	×	×	×	×	×	×		
Physical examination, ECOG performance status[9]	×	×	×	×	×	×	×	×		
New antineoplastic therapy									×	×
Survival status										×
Treatment[10]										
Sintilimab plus Chidamide		×	×	×	×	×				
Laboratory operation / evaluation										
Pregnancy test [11]	×						×			
Routine blood test [12]	×	×	×	×	×	×	×	×		
Biochemical routine [12]	×	×	×	×	×	×	×	×		
Urine routine [12]	×	×	×	×	×	×	×	×		

Stool routine [12]	×	×	×	×	×	×	×	×		
Coagulation function [12]	×	×	×	×	×	×	×	×		
Thyroid function [12]	×	×	×	×	×	×	×	×		
Plasma amylase/lipase [12]	×	×	×	×	×	×	×			
Pulmonary function [13]	×									
Hepatitis B detection [14]	×						×			
HBV-DNA[15]	×	×	×	×	×	×	×	×		
EBV-DNA[16]	×	×	×	×	×	×	×	×		
Efficacy evaluation										
Radiography examination [17]	×		×		×		×		×	
Bone marrow aspiration and biopsy [18]	×						×			
Biomarker collection										
Peripheral blood [19]	×	×	×	×	×	×	×			

Note.

1. End of treatment (EOT): the date of termination of study treatment is defined as the date on which the investigators confirms that the subject needs to terminate the study treatment for any reason. The time window of termination of study treatment visit is +28 days.
2. Safety follow-up: the time window of safety follow-up was 30 days after the last study ± 3 days; If the study treatment termination visit occurs within the time window of safety follow-up, the same examination items need not be repeated.
3. Radiography follow-up: except for the subjects who stopped taking drugs due to the judgment of disease progression according to imaging evaluation, the imaging evaluation was continued according to the established time until the occurrence of imaging disease progression, the start of new anti-tumor treatment, withdrawal of informed consent, death or the end of the study (whichever occurs first)
4. Survival follow-up: after the last study, the survival of the subjects was followed up by telephone every 12 weeks, and the survival of the patients with disease progression was followed up every 8 weeks.
5. Combined use of drugs: The combined medication within 30 days before the screening visit was recorded until the safety follow-up was completed.
6. Adverse events: all AE were recorded from the signing of informed consent until 30 days after the last study treatment; SAE should be recorded until 90 days after the last study treatment or the start of new anti-tumor treatment, whichever occurs first. After that, only SAE related to the study treatment should be recorded.
7. ECG: ECG will be in screening period, treatment period (3 weeks) ± 3 days) and safety follow-up, ECG examination can be added if necessary.
8. Height, weight and vital signs: height examination was only carried out in the screening period, weight examination was only carried out in the screening period, study treatment termination visit and safety follow-up; Vital signs include body temperature, pulse rate, respiratory rate and blood pressure.
9. Physical examination and ECOG score: general physical examination was performed during screening period, study treatment termination visit and safety follow-up, and lymphoma specialist physical examination was performed during treatment.
10. Treatment: should be administered on the first day of each cycle after all clinical and laboratory procedures / assessments are completed.
11. Pregnancy test: fertile female subjects were required to conduct hCG pregnancy test during screening period (within 7 days before the first administration), study treatment termination visit and safety follow-up. When there are clinical indications, follow-up urine pregnancy test should be carried out. If urine pregnancy test is positive, serum pregnancy test should be carried out.
12. Blood routine, biochemical routine, urine routine, blood coagulation, thyroid function was detected at - 2 to 0 days before each cycyle.

13. Pulmonary function: during the screening period (within 28 days before the first administration) and the treatment period (12 weeks) ± 7 days) and safety follow-up.
14. During screening period, patients with hepatitis B positive need to be rechecked in treatment termination and safety follow-up.
15. HBV-DNA positive patients should be monitored every cycle of treatment and treated with nucleoside analogues
16. EBV-DNA： In the screening period, the plasma EBV-DNA was monitored. Before every course of treatment and the first day of the follow-up course were monitored. After all courses of treatment, the patients were monitored once a month according to the fixed time.
17. Radiography evaluation: according to RECIL 2017 lymphoma criteria, the efficacy was evaluated by the researchers. During the screening period and every 6 weeks after the start of the study, enhanced PET / CT or MR or CT examinations were performed in the head and neck (including nasal cavity, hard palate, anterior cranial fossa and nasopharynx), chest, abdomen and pelvis; If you are allergic to CT contrast medium, enhanced MRI can be used. The whole body PET / CT or MR or CT examinations were performed in the screening period, 6, 12, 18 weeks after treatment, repeated every 8 weeks after 6 cycles up to 12 months. Additional PET / CT examination is required if residual lesions or suspicious progression are indicated during enhanced CT follow-up. Except for the subjects who stopped using drugs due to the progress of disease determined by imaging evaluation, imaging evaluation was continued according to the established time until the occurrence of imaging disease progression, the start of new anti-tumor treatment, withdrawal of informed consent, death or the end of the study (whichever occurs first). Within 24 months after the end of the treatment, imaging reexamination was carried out every 12 weeks, and over 24 months, reexamination was carried out every 12-24 weeks.
18. Bone marrow puncture / biopsy: bone marrow puncture / biopsy should be performed in the screening stage and when CR was judged by imaging; If it is not clear whether the bone marrow is invaded under the microscope, flow cytometry or immunohistochemistry can be performed according to the judgment of the researcher. Patients with bone marrow involvement should be reexamined every 6 weeks.
19. Biomarker detection: before each cycle of treatment.

1. Introduction and Background

1.1 Overview of Extranodal NK/T Cell Lymphoma

Extranodal natural killer/T-cell lymphoma (ENKTL) is a highly aggressive form of non-Hodgkin's lymphoma (NHL) closely related to Epstein-Barr virus (EBV)[1,2]. ENKTL is more common in Asia and South America, accounting for 12% among all subtype NHL in China[3,4]. Anthracycline- based chemotherapy is ineffective due to high expression of P-glycol- protein of tumor cells[5]. Asparaginase-based regimen plays a vital role in treatment naïve (TN) early-stage with risk factors or advance and R/R patients[6]. P-Gemox(pegaspargase, gemcitabine, oxaliplatin)[7], SMILE (dexamethasone, methotrexate, ifosfamide, L-asparaginase, and etoposide)[8], AspaMetDex(L-asparaginase, methotrexate, and dexamethasone)[9] and DDGP (cisplatin, dexamethasone, gemcitabine, pegaspargase) [10]regimens were recommended by NCCN guidelines. But optimal therapeutic strategies have not been fully defined, which is the most effective asparaginase-based regimen still controversial. Patients with stage III/IV or refractory/relapsed(r/r) diseases have a poor prognosis. SMILE regimen attained highly response rate for both TN localized and advanced or r/r ENKTL patients, but high prevalence of adverse reactions need attention. Although asparaginase based regimen improved the curative effect to a certain extent. The 5-year survival rate has been determined as less than 30%[11]. It is difficult to break through the bottleneck of curative effect in the era of chemotherapy. Therefore, the development of novel effective therapies or strategies for this population is urgently.

The application of immune checkpoint blockades in various cancers shows surprising efficacy and updates the treatment modality of oncotherapy. PD-1/PD-L1 axis is thought to play a role in immune escaping that results in inefficient immune response targets EBV-positive tumor cells[12]. ENKTL cells are found with upregulation

of PD-L1, and potential mechanism is via EBV triggering of latent membrane protein 1 (LMP1), which then upregulates PD-L1 expression through activation of the mitogen-activated protein kinases pathway/nuclear factor kappa B pathway[13]. PD-1/PD-L1 show promising monotherapy efficacy and well tolerance in ENKTL failing to asparaginase-based treatment in current early-phase trials and retrospective studies[14-18]. The ORR was about 38%-68% and CR rate was about 24%-36%. However, the median progression-free survival (PFS) and duration of response (DOR) are relatively short. Combining other therapies might be an approach to improve the efficacy.

Histone deacetylase inhibitors (HDACi) have shown promising effect in peripheral T cell lymphoma (PTCL). Chidamide an oral subtype-selective histone deacetylase inhibitor (HDACi) has been approved by National Medical Products Administration (NMPA) for the treatment of refractory or relapsed PTCL[19]. In phase I study[20] of chidamide monotherapy in patients with advanced solid tumors and lymphomas. Patients received oral doses of 5, 10, 17.5, 25, 32.5, or 50 mg chidamide either twice (BIW) or three times (TIW) per week for 4 consecutive weeks every 6 weeks. Two incidences of grade 3 diarrhea, nausea and vomiting of first-cycle dose-limiting toxicity (DLT) were observed in the first two patients enrolled in the 50 mg TIW cohort. Thus, gastrointestinal side effects were determined to be DLTs in the 50 mg TIW cohort. No DLTs were observed in other cohorts for both BIW and TIW regimens. In an exploratory phase II trial[21], 19 patients with relapsed or refractory PTCL were randomized to receive 30 or 50 mg twice per week for 2 weeks, followed by 1 week of rest. The ORR was 11.1% for the 30 mg and 40.0% for the 50 mg. One patient in the 50 mg arm experienced drug-related grade 4 thrombocytopenia. Based on the overall results from the phase I and exploratory phase II trials, patients in the pivotal phase II study[22] were orally administered with chidamide of 30mg. However, efficacy

and safety of HDACi in ENKTL are controversial. From November 2016 to February 2018, we enrolled 15 patients with r/r ENKTL treated with three therapeutic dose modes of single-agent chidamide (30mg twice a week; 20mg every other day; 10mg once a day, respectively). ORR was 50.0% (6/12) with CR rate 33.3% (4/12). Three patients got CR in 30mg group; one patient got CR in 10mg group. The median time to response was 5.9 (1.1 to 8.1) weeks. Median follow-up time was 3.7 months (0.8-11.5). Our study results suggested that chidamide was effective as single agent in the treatment of r/r ENKTL[23].

Recent studies on the role of epigenetics in immune evasion have exposed a key role for epigenetic modulators in augmenting the tumor microenvironment and restoring immune recognition and immunogenicity[24]. Moreover, accumulating evidences have shown the synergism between epigenetic drugs and anti-PD-1 antibody[25]. Recent studies have shown that in combination with epigenetic modulators, such as histone deacetylase inhibitors (HDACi) or DNA methyltransferases (DNMTi), can reverse the resistance or ineffectiveness of immune checkpoint inhibitors, supported by both in vitro and in vivo studies. Therefore, targeted epigenetics maybe a therapeutic strategy to improve the efficacy of immunotherapy in RR-ENKTL.

1.2 Description of Investigational Study Drug

1.2.1 Chidamide

Chidamide, an oral subtype-selective histone deacetylase inhibitor (HDACi) has been approved by National Medical Products Administration (NMPA) for the treatment of refractory or relapsed PTCL in December 2014[26]. As a selective inhibitor of HDAC1, 2, 3, and 10, Chidamide could increase the acetylation level of chromatin histones to trigger chromatin remodeling and thereby change the gene expression of multiple signaling pathways. Chidamide can also enhance the tumor killing effect mediated by

natural killer cells (NK) and antigen-specific cytotoxic T cells (CTL) to promote anti-tumor immune function. In addition, compared to other types of HDACi, Chidamide can induce differentiation of cancer stem cell and reverse the epithelial-mesenchymal phenotypic transformation (EMT) of tumor cells, thus reversing drug-resistance and inhibiting tumor metastasis and recurrence.

1.2.2 Sintilimab

Sintilimab (R&D code: IBI308) is a recombinant fully human IgG4 PD-1 monoclonal antibody that can bind to the PD-1 receptor to block its interaction with PD-L1 and PD-L2, thus hindering the immunosuppressive response mediated by PD-1 pathway, including anti-tumor immune response. Blocking PD-1 activity with sintilimab can inhibit tumor growth both in animal models and preclinical in vitro tests. Base on the results of completed preclinical pharmacodynamics, animal pharmacokinetics and toxicology studies have demonstrated sintilimab with a clear target and drug stability. For detailed research results, please refer to the investigator's brochure. A multi-center, single-arm phase II clinical study (ORIENT-1) of Sintilimab in relapsed or refractory classic Hodgkin's lymphoma has demonstrated Sintilimab to be efficient and safe. At present, a number of phase III clinical studies of single or combined medication of sintilimab have been carried out in various tumors.

References

1. Peng R-J, Han B-W, Cai Q-Q, et al. Genomic and transcriptomic landscapes of Epstein-Barr virus in extranodal natural killer T-cell lymphoma. *Leukemia* 2019; **33**(6): 1451-62.
2. Lin N, Ku W, Song Y, Zhu J, Lu Z. Genome-Wide Analysis of Epstein-Barr Virus Isolated from Extranodal NK/T-Cell Lymphoma, Nasal Type. *Oncologist* 2019; **24**(9): e905-e13.
3. Sun J, Yang Q, Lu Z, et al. Distribution of lymphoid neoplasms in China: analysis of 4,638 cases according to the World Health Organization classification. *Am J Clin Pathol* 2012; **138**(3): 429-34.
4. Liu W, Liu J, Song Y, et al. Mortality of lymphoma and myeloma in China, 2004-2017: an observational study. *J Hematol Oncol* 2019; **12**(1): 22.
5. Yamaguchi M, Kita K, Miwa H, et al. Frequent expression of P-glycoprotein/MDR1 by nasal T-cell lymphoma cells. *Cancer* 1995; **76**(11): 2351-6.
6. Pokrovsky VS, Vinnikov D. L-Asparaginase for newly diagnosed extra-nodal NK/T-cell lymphoma: systematic review and meta-analysis. *Expert Rev Anticancer Ther* 2017; **17**(8): 759-68.
7. Wang L, Wang Z-h, Chen X-q, et al. First-line combination of gemcitabine, oxaliplatin, and L-asparaginase (GELOX) followed by involved-field radiation therapy for patients with stage IE/IIE

extranodal natural killer/T-cell lymphoma. *Cancer* 2013; **119**(2): 348-55.
8. Kwong Y-L, Kim WS, Lim ST, et al. SMILE for natural killer/T-cell lymphoma: analysis of safety and efficacy from the Asia Lymphoma Study Group. *Blood* 2012; **120**(15): 2973-80.
9. Jaccard A, Gachard N, Marin B, et al. Efficacy of L-asparaginase with methotrexate and dexamethasone (AspaMetDex regimen) in patients with refractory or relapsing extranodal NK/T-cell lymphoma, a phase 2 study. *Blood* 2011; **117**(6): 1834-9.
10. Li X, Cui Y, Sun Z, et al. DDGP versus SMILE in Newly Diagnosed Advanced Natural Killer/T-Cell Lymphoma: A Randomized Controlled, Multicenter, Open-label Study in China. *Clin Cancer Res* 2016; **22**(21): 5223-8.
11. Fox CP, Civallero M, Ko Y-H, et al. Survival outcomes of patients with extranodal natural-killer T-cell lymphoma: a prospective cohort study from the international T-cell Project. *Lancet Haematol* 2020; **7**(4): e284-e94.
12. Bi X-W, Wang H, Zhang W-W, et al. PD-L1 is upregulated by EBV-driven LMP1 through NF-κB pathway and correlates with poor prognosis in natural killer/T-cell lymphoma. *J Hematol Oncol* 2016; **9**(1): 109.
13. Song TL, Nairismägi M-L, Laurensia Y, et al. Oncogenic activation of the STAT3 pathway drives PD-L1 expression in natural killer/T-cell lymphoma. *Blood* 2018; **132**(11): 1146-58.
14. Kwong Y-L, Chan TSY, Tan D, et al. PD1 blockade with pembrolizumab is highly effective in relapsed or refractory NK/T-cell lymphoma failing l-asparaginase. *Blood* 2017; **129**(17): 2437-42.
15. Tao R, Fan L, Song Y, et al. SINTILIMAB FOR RELAPSED/REFRACTORY (R/R) EXTRANODAL NK/T CELL LYMPHOMA (ENKTL): A MULTICENTER, SINGLE-ARM, PHASE 2 TRIAL (ORIENT-4). *Hematological Oncology* 2019; **37**(S2): 102-3.
16. Huang H-q, Tao R, Zou L, et al. Preliminary Results from a Multicenter, Single-Arm, Phase 2 Study of CS1001, an Anti-Programmed Death-Ligand 1 (PD-L1) Human Monoclonal Antibody (mAb), in Patients (pts) with Relapsed or Refractory Extranodal Natural Killer/T Cell Lymphoma (rr-ENKTL). *Blood* 2019; **134**(Supplement_1): 2833-.
17. Shi Y, Wu J, Wang Z, et al. Efficacy and safety of geptanolimab (GB226) for relapsed or refractory peripheral T cell lymphoma: an open-label phase 2 study (Gxplore-002). *J Hematol Oncol* 2021; **14**(1): 12.
18. Kim SJ, Lim JQ, Laurensia Y, et al. Avelumab for the treatment of relapsed or refractory extranodal NK/T-cell lymphoma: an open-label phase 2 study. *Blood* 2020; **136**(24): 2754-63.
19. Shi Y, Jia B, Xu W, et al. Chidamide in relapsed or refractory peripheral T cell lymphoma: a multicenter real-world study in China. *J Hematol Oncol* 2017; **10**(1): 69.
20. Dong M, Ning ZQ, Xing PY, et al. Phase I study of chidamide (CS055/HBI-8000), a new histone deacetylase inhibitor, in patients with advanced solid tumors and lymphomas. *Cancer Chemother Pharmacol* 2012; **69**(6): 1413-22.
21. Shi Y-K, Dong M, Hong X-N, et al. Phase II study of chidamide (CS055), a new subtype-selective oral histone deacetylase inhibitor, in patients with relapsed or refractory peripheral T-cell lymphoma. *Journal of Clinical Oncology* 2013; **31**(15_suppl): 8525-.
22. Shi Y, Dong M, Hong X, et al. Results from a multicenter, open-label, pivotal phase II study of chidamide in relapsed or refractory peripheral T-cell lymphoma. *Ann Oncol* 2015; **26**(8): 1766-71.
23. Yan G, Huang H-q, Li P, et al. Chidamide ,Oral Subtype-Selective Histone Deacetylase Inhibitor (HDACI) Monotherapy Was Effective on the Patients with Relapsed or Refractory Extranodal Natural Killer (NK)/T-Cell Lymphoma. *Blood* 2017; **130**(Supplement 1): 2797-.
24. Li Z, Xia Y, Feng L-N, et al. Genetic risk of extranodal natural killer T-cell lymphoma: a genome-wide association study. *Lancet Oncol* 2016; **17**(9): 1240-7.
25. Chen X, Pan X, Zhang W, et al. Epigenetic strategies synergize with PD-L1/PD-1 targeted cancer immunotherapies to enhance antitumor responses. *Acta Pharm Sin B* 2020; **10**(5): 723-33.
26. Shi Y, Dong M, Hong X, et al. Results from a multicenter, open-label, pivotal phase II study of chidamide in relapsed or refractory peripheral T-cell lymphoma. *Ann Oncol* 2015; **26**(8): 1766-71.

2. Study Objectives and Endpoints

**Objectives**	**Endpoints**
**Primary**	
• Evaluate the efficacy of sintilimab	Overall response rate (ORR) according to the RECIL

plus chidamide in patients with RR-ENKTL.<br>• Identify the maximum tolerated dose (MTD), dose limited toxicities (DLTs) and recommended Phase II dosage (RP2D) of chidamide in combination with sintilimab.	2017 Response Criteria for Malignant Lymphoma as determined by investigators in all treated patients; ORR is defined as the proportion of patients with a best overall response of complete response (CR) or partial response (PR).
**Secondary**	
• Long-term survival of sintilimab plus chidamide in patients with RR-ENKTL.<br>• The safety profile of sintilimab plus chidamide.	• Progression-Free Survival (PFS) is defined as the time from the treatment date to the date of recurrence, disease progression or death.<br>• Duration of Response (DoR) is defined as the date of their first objective response (which is subsequently confirmed) to disease progression or Among participants who experience an objective response.<br>• Overall Survival (OS) is defined as the time from treatment to the date of death.<br>• Frequency and severity of adverse events (AEs) and serious adverse events (SAEs)
**Exploratory**	
Explore correlations between clinical activity or tolerability and tumor and/or blood biomarkers, including pharmacogenetic markers.	• Blood sampling for dynamic ctDNA<br>• Tumor tissue for biomarkers (e.g., immunohistochemistry of PD-L1 expression)

3. Study Design

3.1 Overall Study design

This is a phase Ib/II study including dose exploration, followed by the multi-center, open-label extended study.

The dose exploration of the Phase Ib study, a standard “3+3” design was utilized to identify the maximum tolerated dose (MTD), dose limited toxicities (DLTs) and recommended Phase II dosage (RP2D) of chidamide in combination with sintilimab. Once the MTD, DLT and RP2D of Chidamide is determined, the Phase II study shall begin.

The phase II study is proceeded according to the RP2D of chidamide. Patients that achieved CR and PR could receive for up to one-year treatment, otherwise until disease progression, death, intolerable toxicity, completion of the scheduled treatment cycle, or the investigators' decision to withdraw (whichever occurs first). After 12 months, patients still with CR or PR were allowed to choose to continue or end of treatment or accepted autologous hematopoietic stem cell transplantation (ASCT) as consolidation (Stem cell mobilization, collection, reinfusion, conditioning regimen prior to autologous hematopoietic stem cell transplantation followed the medical routine at each study site). If patients choose to continue sintilimab plus chidamide, doses of chidamide reduce to 20mg twice a week and fixed doses of 200mg sintilimab intravenously every 30 days. Dose interruptions were allowed for treatment-related adverse events until these events resolved to grade 0–1 or to baseline level. Treatment was permanently discontinued for patients who had interruptions of more than 4 weeks. Patients will be visited for end of treatment within 4 weeks after the end of the study. For those whose condition is stable or improved after treatment, progression-free survival (PFS) follow-up will be carried out 8 weeks after the last administration of the study drug, and

continued every 12 weeks thereafter until disease progression, death, withdrawal of informed consent (ICF), receiving other novel treatments, or termination of the entire study (whichever occurs first). At the same time, patients who have developed PD will be followed up for overall survival (OS) every 12 weeks until death, withdrawal of ICF, or the termination of the entire study (whichever occurs first). The survival follow-up is conducted every 8 weeks for patients with disease progression.

Patients will receive efficacy measurements, including ORR, PFS, OS, duration of response (DoR). During the entire study, efficacy will be evaluated based on International Working Group consensus response evaluation criteria in lymphoma (RECIL 2017). Patients underwent CT, MRI or whole body ^{18}F-fluorodeoxyglucose (FDG) PET scans before the first treatment and then every 6 weeks up to cycle 6, and every 12 weeks thereafter. A whole-body PET scan was strong recommended. Efficacy evaluation will be performed every 2 treatment cycles (i.e. 6 weeks) until the completion of 6 cycles of treatment, repeated every 8 weeks after 6 cycles up to 12 months, or disease progression, intolerable toxicity, death, investigators' decision, or the patient's voluntary withdrawal from the study (whichever occurs first).

Safety observation indicators include monitoring and recording of adverse events (TEAE and SAE) during treatment, performing laboratory tests (including blood routine, biochemical and urine tests), 12-lead electrocardiogram, and vital signs as specified in the design. Adverse events will be evaluated according to the NCI CTCAE (version 5.0). The safety and tolerability of the research drugs will be evaluated through laboratory and auxiliary examinations, physical and vital signs examinations. The design and flow chart of this study are shown in **Figure 1**.

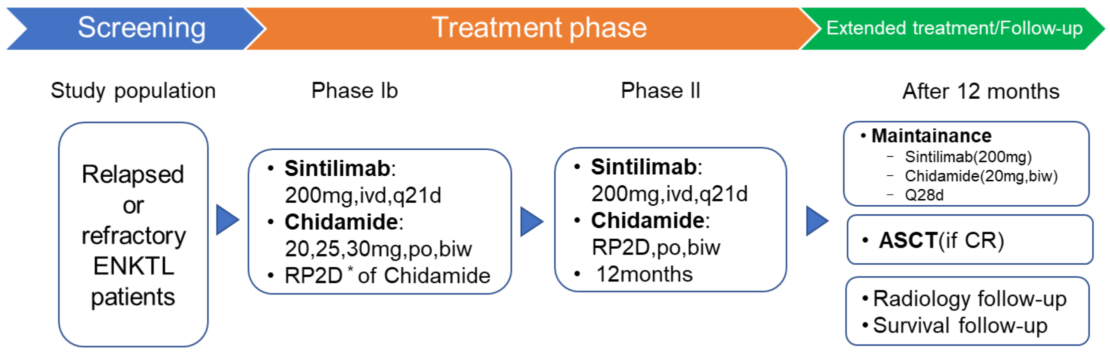

Figure1. Flow chart of this study

3.2 Screening Period

Informed consent must be obtained for each patient and documented with a signed informed consent form (ICF) prior to any study procedures. The screening period is from 28 days to 1 day prior to the start of the study drug. The screening assessments should be performed within this period in order to assess the eligibility of the patient against the inclusion and exclusion criteria.

3.3 Treatment Period

In phase Ib, patients received oral escalating doses of chidamide (20, 25, 30 mg) twice a week continuous and fixed doses of 200mg sintilimab intravenously over a period of 30–60 min, once every 21 days. If DLTs does not occur, the established dose escalation plan could be proceeded to the next level. If one case of DLT occurs, 3 more subjects should be added to this dose level until there is no new DLT report. If there are more than 2 cases of DLT, the forward dose level of this dose level is defined as MTD. The dose level of Sintilimab is 200 mg (fixed dose) each time and repeated every three weeks. Patients that achieved CR and PR could receive for up to one-year treatment. Patients defined with SD and PD should withdraw from the study.

The phase II study is proceeded according to the RP2D of Chidamide. Patients that achieved CR and PR could receive for up to 12 months treatment, otherwise until disease progression, death, intolerable toxicity, completion of the scheduled treatment cycle, or the investigators' decision to withdraw (whichever occurs first).

3.4 End of Treatment

End of Treatment visit (EOT) should be performed as soon as possible after the decision to discontinue the study drug, preferably within 30 days after last dose of study drug and before initiation of any new anticancer treatment.

3.5 Follow-up Period

All patients, regardless of disease status, will be visited for end of treatment within 4 weeks after the end of the study, followed every 12 weeks for up to 3 years, or until withdrawal of consent, loss to follow-up, or death, whichever occurs first. When disease assessments are not planned for a follow-up visit, the visit can be done by phone.

3.6 End of Study

The study will be ended 8 weeks after the last medication of the last enrolled patient or when the disease progresses (except for the withdrawal of ICF).

4. Patient selection

4.1 Inclusion criteria

1. Subjects who have fully understood the study and have signed the informed consent form (ICF).
2. Male or female, age over 18 years (including 18) old and no more than 75 years old.
3. Extranodal NK/T-cell lymphoma confirmed by histopathology examination.
4. Refractory and relapsed ENKTCL that has failed the treatment of asparaginase-based chemotherapy or radiochemotherapy.

Definition of refractory:

i. Failure to get PR after chemotherapy with asparaginase-containing regimen; or

ii. Disease progression within 6 months of the final asparaginase-containing regimen.

5. Subjects with Eastern Cooperative Oncology Group (ECOG) performance status score of 0 or 1;
6. The expected survival time is over 3 months.
7. There must be at least 1 evaluable or measurable lesion that meets the RECIL 2017 lymphoma criteria [evaluable lesion: 18F-Fluorodeoxyglucose/Positron Emission Tomography (18F-Fluorodeoxyglucose/Positron Emission Tomography, 18FDG/PET) examination showing increased lymph nodes or extranodal uptake (higher than liver) and PET and/or Computed Tomography (CT) features are consistent with lymphoma manifestations; measurable lesions: nodular lesions longer than 15mm or extranodal lesions longer than 10mm (if the only measurable lesion has received radiotherapy in the past, there must be evidence of radiological progression after radiotherapy) and accompanied by increased 18FDG uptake]. Except for this, there is no measurable increase in the diffuse 18FDG uptake in the liver.
8. Subjects should have adequate hematological, hepatic, and renal function as defined by the following laboratory examinations. Subjects have not received cell growth factors, platelet or granulocyte infusion within 7 days before hematological examinations (for the long-acting granulocyte-colony stimulating factor [G-CSF] and [PEG-CSF], the interval of 2 weeks is required):
   a) Absolute neutrophil count $\geq 1.5 \times 10^9$ / L for subjects without marrow involvement of lymphoma; absolute neutrophil count $\geq 1.0 \times 10^9$ / L for subjects with marrow involvement;
   b) Hemoglobin ≥ 90 g/ L (without red blood cell infusion within 14 days),

hemoglobin ≥ 75 g/ L for subjects without marrow involvement of lymphoma;

c) Platelet count ≥ 75 × 10^9 / L for subjects without marrow involvement of lymphoma; platelet count ≥ 50 × 10^9 / L for subjects with marrow involvement;
d) Upper Limit Normal (ULN), or creatinine clearance (≥ 40 mL/min) of serum creatinine (≤ 1.5 times normal value upper limit) (estimated by the Cockcroft-Gault formula);
e) Serum total bilirubin ≤ 1.5 times ULN;
f) Aspartate Aminotransferase (AST), Alanine Aminotransferase (ALT) ≤2.5 times ULN;
g) Coagulation function: International Normalized Ratio (INR) ≤ 1.5 times ULN; Prothrombin Time (PT), Activated Partial Thromboplastin Time (APTT) ≤ 1.5 times ULN (unless the subject is receiving anticoagulant therapy and PT and APTT are using anticoagulant therapy at the screening time).
h) Thyroid-stimulating hormone (TSH) or free thyroxine (FT4) or free triiodothyronine (FT3) are all within the normal range (±10%).

9. There was no evidence that subjects had difficulty breathing at rest, and the measured value of pulse oximetry at rest was more than 92%;
10. Subjects must pass a pulmonary function test (PFT) to confirm that forced expiratory volume (FEV1)/forced vital capacity (FVC) in the first second is more than 60%, unless it is a large mediastinal mass caused by lymphoma that cannot meet this standard; carbon monoxide diffusion (DLCO), FEV1 and FVC are all above 50% of the predicted value; all PFT results must be obtained within four weeks before the first administration;

11. Subjects who have received anti-tumor therapy should be screened only after the toxicity of the previous treatment has recovered to the grade 1 level or baseline level according to Common Terminology Criteria for Adverse Events (CTCAE) V5.0;
12. Women of Childbearing Potential (WOBCP) must undergo a serum pregnancy test within seven days before the first medication and the results are negative. WOBCP or men and their WOBCP partners should agree to take effective contraceptive measures from the signing of ICF until six months after the last dose of the research drug is used.

4.2 Exclusion Criteria:

1. Invasive natural killer cell leukemia;
2. Active hemophagocytic syndrome (Suffered from hemophagocytic syndrome previously but recovered more than 6 months could enrolled.);
3. Primary central nervous system lymphoma or secondary central nervous system involvement;
4. Received organ transplantation in the past;
5. Patients who received allogeneic hematopoietic stem cell transplantation within three years;
6. Adverse reactions from the previous anti-tumor treatment have not yet recovered to ≤Grade 1 based on NCI-CTCAE [Version 5.0], except for hair loss and pigmentation;
7. Autologous hematopoietic stem cell transplantation was performed within 90 days before the start of the study;
8. Patients with active autoimmune diseases requiring systematic treatment in the past two years (hormone replacement therapy is not considered systematic

treatment, such as type I diabetes mellitus, hypothyroidism requiring only thyroxine replacement therapy, adrenocortical dysfunction or pituitary dysfunction requiring only physiological doses of glucocorticoid replacement therapy); Patients with autoimmune diseases who do not require systematic treatment within two years can be enrolled;

9. Subjects requiring systemic glucocorticoid therapy or other immunosuppressive therapy for a given condition within 14 days before treatment [allowing subjects to use local, ocular, intra-articular, intranasal and inhaled glucocorticoid therapy (with very low systemic absorption); and allowing short-term (< 7 days) glucocorticoid prophylaxis (e.g., contrast agent overdose) Sensitivity] or for the treatment of non-autoimmune diseases (e.g. delayed hypersensitivity caused by contact allergens);
10. In the past five years, patients with other malignant tumors have undergone radical treatment, except for basal cell carcinoma of skin, squamous cell carcinoma of skin, carcinoma in situ of breast and carcinoma in situ of cervix.
11. Subjects have major surgery within four weeks prior to enrollment or those who have not completely recovered from any previous invasive operation;
12. Uncontrolled hypertension (the systolic pressure > 180 mmHg and/or diastolic pressure >100 mmHg after treatment);
13. Active hemorrhagic diseases;
14. Begin research on live vaccination (except influenza attenuated vaccine) within 28 days before treatment;
15. Human immunodeficiency virus (HIV) infection (HIV positive);
16. Patients with active hepatitis B or active hepatitis C. Patients who are positive for hepatitis B Surface Antigen (HBsAg) or hepatitis C Virus (HCV) antibodies

at screening stage must pass further detection of hepatitis B Virus (HBV) DNA titer (no more than 1000 copies/mL or 500 IU/mL) and HCV RNA (no more than the lower limit of the detection method) in the row. In addition to active hepatitis B or hepatitis C infections requiring treatment, group trials can be conducted. Hepatitis B carriers, stable hepatitis B (DNA titer should not be higher than 2500 copies/mL or 500 IU/mL) after drug treatment, and cured hepatitis C patients can be enrolled in the group;

17. Patients with active pulmonary tuberculosis;
18. Known history of hypersensitivity to a similar drug and any excipient in the study drug;
19. Pregnant or lactating women;
20. Have uncontrollable complications, including but not limited to symptomatic congestive heart failure, uncontrollable hypertension, unstable angina, active peptic ulcer or hemorrhagic diseases;
21. Any severe or uncontrolled systematic disease, systematic complication, other severe complicated diseases, or any special condition that might make the subjects unsuitable for the study, or might affect the compliance to the protocol or might cause significant interference in safety/ efficacy evaluation of the study drug as judged by the investigator.

4.3 Screening Failures:

Patients who signed the ICF but were found not eligible for the study prior to receiving study drug are defined as screening failures. For these patients, only limited information will be collected in the case report form (CRF):

- Informed consent
- Demographics

- Inclusion/exclusion criteria

- SAE and/or death occurring during the screening period.

5 Treatment

5.1 Dosage and Treatment cycle of Study drug

5.1.1 Phase Ib Study

Objective: To determine the MTD and DLT/RP2D of Chidamide.

Methods: Traditional “3+3”design.

Sample Size: 9-18 patients

Design	Method Description
“3+3”	1. Starting from the initial dose, there are 3 subjects at each dose level;<br>2. Proceed to the next level of dose level test according to the established dose escalation plan, if DLT does not occur.<br>3. If 1 case of DLT occurs, 3 more subjects are added to the dose level. Proceed to the next dose level test once no new DLT appears.<br>4. If more than 2 cases of DLT occur, the forward dose level of this dose level is defined as MTD.<br>5. MTD is defined as when at a dose level less than 2 cases of DLT out of 6 subjects.

RP2D is determined according to MTD and DLT.

- Initial dose of chidamide: 3 dose groups (20mg, 25mg, 30mg) with 3 to 6 subjects in each group. The starting dose is 20 mg per dose twice a week for continuous oral administration. If DLT does not occur, follow the established dose escalation plan to proceed to the next dose level test. If there is 1 case of DLT, add 3 more subjects to this dose level. When no new DLT appears, proceed to the next dose level. If there are more than 2 cases of DLT, the forward dose level of this dose level is defined as MTD.
- Sintilimab dose: 200mg/time (fixed dose)

Note:

- The above drugs are repeated every 3 weeks.
- Patients that achieved CR and PR could receive for up to one-year treatment using sintilimab combined with chidamide or until disease progression, intolerable toxicity, death, or termination of the study for any reason. Patients with SD and PD withdrew from the study.

5.1.2 Phase II study

Objective: To evaluate the short-term objective response rate, long-term efficacy, and safety of sintilimab combined with chidamide in the treatment of refractory and relapsed ENKTCL patients.

Number of subjects: 28 patients.

Administration of study drug: The Phase II study was conducted according to the RP2D of chidamide from Phase Ib clinical study.

- Chidamine: During the combined treatment, the dose is administered according to the recommended dose of RP2D.
- Sintilimab dose: 200mg (fixed dose)

Note:

- The above drugs are repeated every 3 weeks.
- Patients that achieved CR and PR could receive for up to one-year treatment using sintilimab combined with chidamide or until disease progression, intolerable toxicity, death, or termination of the study for any reason. Patients with SD and PD withdrew from the study.
- After 12 months, patients still with CR or PR were allowed to choose to continue or end of treatment or accepted autologous hematopoietic stem cell transplantation (ASCT) as consolidation (Stem cell mobilization, collection, reinfusion, conditioning regimen prior to autologous hematopoietic stem cell transplantation followed the medical routine at each study site).
- If patients choose to continue sintilimab plus chidamide, doses of chidamide reduce to 20mg twice a week and fixed doses of 200mg sintilimab intravenously every 30 days.

5.2. Packaging and storage of sintilimab

The main active ingredient of sintilimab is a recombinant fully human anti-programmed death receptor 1 monoclonal antibody with a concentration of 10 mg/mL. This product is a clear, colorless liquid, free of foreign matter, flocculation and precipitation. Excipients include 140 mmol/L mannitol, 25 mmol/L histidine, 20 mmol/L sodium citrate dihydrate, 50 mmol/L sodium chloride, 0.02 mmol/L edetate disodium (ethylenediamine tetra Disodium acetate), 0.2 mg/mL polysorbate 80, pH 6.0.

The smallest packaging unit of sintilimab is a box, and each box contains one

Sintilimab injection packaged in a vial. The packaging box is printed with the name of the drug, dosage form, specification, drug code, production batch number, expiration date, storage conditions and information of the sponsor. The same information is printed on the label of the vial and the box, but there is no information of the dosage form, precautions, usage and dosage on the label of the vial. The labels of all packaging boxes and vials are marked with "For clinical research use only". Sintilimab products are stored in the dark at 2~8℃, and the validity period is 24 months. If there are quality problems such as turbidity and precipitation in the injection, it should be sealed up immediately and Innovent Company should be notified immediately.

The preparation and infusion process of sintilimab is as follows:

1. Draw out 2 bottles of sintilimab injection completely, add 100mL 0.9% (weight/volume) sodium chloride sterile saline intravenous infusion bag, and record the time of preparation start.
2. Gently invert the infusion bag to mix to ensure the uniformity of the medicine in the infusion bag and avoid foaming due to violent shaking. If a large amount of foam is generated, the drug should be allowed to stand until the foam disappears.
3. The drug is administered through an online filter equipped with 0.2~1.2μm (the infusion time is recommended to be controlled within 30-60 minutes), and the start and end time of the drug delivery are recorded.

Note:

- Before configuration, confirm that the sintilimab injection is transparent and there are no quality problems such as turbidity or precipitation;
- Ensure that the time from the first bottle of sintilimab injection to the end of administration does not exceed 24 hours (the storage of the prepared drug should be kept refrigerated at 2~8℃);

- Avoid mixing other drugs;
- Avoid intravenous bolus injection.

5.3 Toxicities of sintilimab

The safety summary data of Sintilimab came from 8 clinical studies conducted in China. A total of 1444 patients received at least one dose of Sintilimab, of whom 520 received Sintilimab monotherapy. Most patients received Sintilimab at a dose of 200 mg every 3 weeks. Among 520 patients who received Sintilimab monotherapy, the incidence of adverse reactions was 90.0%. Adverse reactions with an incidence ≥10% included: fever, thyroid function test abnormal, aspartate aminotransferase increased, alanine aminotransferase increased, proteinuria, rash, hypoalbuminemia, decreased appetite, hyperglycemia, γ-glutamyltransferase increased, blood bilirubin increased, lung infection, hypokalemia and neutropenia.

Grade 3 and above adverse reactions occurred in 28.7%, and 21% included pulmonary infection, anemia, pancreatitis, hypokalemia, hypertension, pneumonitis, increased γ-glutamyltransferase, decreased appetite, thrombocytopenia, neutropenia, increased aspartate aminotransferase, increased alanine aminotransferase, increased amylase, rash, abnormal liver function, and fever.

For more information, please refer to the drug manual.

5.4 Premedication of sintilimab

Among 2461 patients who had received sintilimab treatment, a total of 85 patients (3.5%) developed infusion reactions, including 40 patients (1.6%) with grade 1, 32 patients (13%) with grade 2, 11 patients (0.4%) with grade 3, and 2 patients (0.1%) with grade 4, and no grade 5 cases occurred. All were given observation or symptomatic care. Four patients (0.2%) permanently discontinued treatment, and 5 patients (0.2%) suspended the administration. The patient recovered.

The incidence of infusion reactions is low, pretreatment is not recommended. In exceptional cases, antihistamines may be administered to prevent infusion reactions.

5.5 Dose adjustment

If any of the following conditions occurs, the dose of the research drug needs to be reduced in the next treatment cycle:

- Grade 4 hematotoxicity once
- Grade 3 hematotoxicity twice
- Grade 3 non-hematological toxicity once (except for alopecia).

During the combined treatment, chidamide is mainly adjusted according to hematotoxicity and non- hematotoxicity. Chidamide is reduced by 5 mg each time, and the dose could be reduced twice at most. (The standard of dose reduction is shown in the table below).

Study Drug	Initial Dosage	First Reduction	Second Reduction
Chidamide	RP2D (eg 30mg)	25mg	20mg

The dose of sintilimab is not allowed to be adjusted during the entire study. The principles of suspension and permanent discontinuation of Sintilimab are shown in the Table1 below.

Table 1. Sintilimab Dose Adjustment Principle

Adverse events related to sintilimab	Severity	Dose adjustment
Pneumonia	Grade 2 pneumonia	suspension [a]
	Recurrent Grade 2 pneumonia, Grade 3 or 4 pneumonia	permanent discontinuation
Diarrhea or enterocolitis	Grade 2 or 3 diarrhea or enterocolitis	suspension [a]
	Grade 4 diarrhea or enterocolitis	permanent discontinuation
Dermatitis	Grade 3 dermatitis	suspension [a]
	Grade 4 dermatitis	permanent discontinuation
Hepatitis	For subjects with normal baseline ALT, AST, or TBIL, Grade 2 elevation of AST, ALT, or TBIL. For subjects with baseline AST, ALT, or TBIL>ULN, ≥50% increase of AST, ALT or TBIL (to meet the requirements of Grade 2) and duration less than 7 days.	suspension [a]
	For subjects with normal baseline ALT, AST, or TBIL, Grade 3 or 4 elevation of AST, ALT, or TBIL. For subjects with baseline AST, ALT, or TBIL>ULN, ≥50% increase of AST, ALT or TBIL (to meet the requirements of Grade 3 or 4) and duration over 7 days.	permanent discontinuation
Hypophysitis	Grade 2 hypophysitis	suspension [b]

	Grade 3 or 4 hypophysitis	permanent discontinuation
Adrenal insufficiency	Grade 2 adrenal insufficiency	suspension [b]
	Grade 3 or 4 adrenal insufficiency	permanent discontinuation
Hyperthyroidism	Grade 3 or 4 hyperthyroidism	permanent discontinuation
Type 1 diabetes	Grade 3 hyperglycemia	suspension [b]
	Grade 4 hyperglycemia	permanent discontinuation
Renal insufficiency	Grade 2 or 3 Elevated creatinine	suspension [a]
	Grade 4 Elevated creatinine	permanent discontinuation
Neurotoxicity	Grade 2 neurotoxicity	suspension [a]
	Grade 3 or 4 neurotoxicity	permanent discontinuation
Other AEs	First appearance of other Grade 3 AEs	suspension [a]
	Recurrence of the same Grade 3 AE	permanent discontinuation
	Grade 3 AE: Failure to decrease to Grade 0-2/baseline level within 7 days or return to Grade 0-1/baseline level within 14 days	permanent discontinuation
	Grade 4 AE	permanent discontinuation [c]

Note

a: Resume the administration after the symptoms improvement to grade 0-1 or baseline level.

b: For hypophysitis, adrenal insufficiency, thyroid insufficiency/hypothyroidism, and type 1 diabetes, resume the administration if they are fully controlled and only need physiological hormone replacement therapy.

c: For abnormal grade 4 laboratory test results, the decision whether to terminate the medication should be determined based on the accompanied clinical symptoms or signs and the clinical judgment of the investigator.

The maximum interval allowed for drug suspension is 12-weeks. If it is not possible to recover to the state where sintilimab can be reused within 12 weeks, the subject will be permanently suspended for sintilimab administration and then enter the follow-up phase. Except for the following two cases:

- Due to the application of glucocorticoids for the treatment of irAE, sintilimab was suspended for more than 12 weeks during the process of glucocorticoid reduction. Under this condition, it is necessary to discuss with the sponsor to decide whether to continue sintilimab treatment. The radiological examination for efficacy evaluation is still carried out as planned and is not affected by the suspension of medication.
- Due to treatment of AEs unrelated to sintilimab, it was suspended for more than 12 weeks. Under this condition, it is necessary to discuss with the sponsor to decide whether to continue sintilimab treatment. The radiological examination for efficacy evaluation is still carried out as planned and is not affected by the suspension of medication.

At the beginning of each treatment course, all hematological and non-hematological toxicities must be restored to no more than Grade 1 or the baseline level and the investigator considers it appropriate to proceed to the next course. If this condition is ineligible, the administration can be extended for 14 days. Once it’s over 14 days, the investigator will decide whether the patient needs to withdraw from the study based on the risk-benefit ratio.

5.6 Management of sintilimab –related infusion reactions

Sintilimab may cause severe or life-threatening infusion reactions, including severe hypersensitivity or allergic reactions. Signs and symptoms usually appear during

or shortly after the infusion of the drug and can usually be completely resolved within 24 hours after the completion of infusion. Guidelines for the management of sintilimab-related infusion reactions are shown in the table below.

Table 2. Guidelines for the management of sintilimab-related infusion reactions

**NCI CTCAE** Grade	Treatment	Pretreatment medication during subsequent administration
**Grade 1** Mild reaction; no need to interrupt the infusion; no intervention.	Strengthen the monitoring of the patient's vital signs according to their medical indications, until the researcher believes that the subject's condition is stable.	None
**Grade 2** Treatment or interruption of the infusion while symptomatic treatment (such as antihistamines, non-steroidal anti-inflammatory drugs [NSAIDS], anesthetics, intravenous fluids) should be given as soon as possible to achieve a rapid response. (2) ≤24 hours of preventive medication should be taken.	Stop the infusion and monitor the symptoms. Other appropriate medications may include but are not limited to: intravenous infusion of antihistamines, NSAIDS, Paracetamol anesthetic.<br>Strengthen the monitoring of the patient's vital signs according to their medical indications, until the researcher believes that the subject's condition is stable.<br>If the symptoms are relieved within one hour after discontinuing the drug infusion, the infusion can be restarted at 50% of the original infusion rate (for example, reduced from 100 mL/h to 50 mL/h). Otherwise, the medication should be suspended until the symptoms are relieved, and the subject should receive pretreatment medication before the next scheduled dosing. For subjects who still have Grade 2 toxicity even after receiving adequate pretreatment medication, further research drug treatment should be permanently stopped.	Subjects can receive the following pretreatment medications 1.5 hours (±30 minutes) before sintilimab infusion:<br><br>Oral 50 mg diphenhydramine (or an equivalent dose of antihistamine).<br>Oral administration of 500-1000 mg paracetamol (or equivalent dose of antipyretic).
**Grade 3 or 4**<br>Grade 3: Long duration (that is, failure to respond quickly after symptomatic medication and/or short-term interruption of infusion); symptoms relapse after initial improvement; hospitalization is required due to other clinical sequelae (such as kidney damage, lung infiltration).<br>Grade 3:<br>Life-threatening; requires hypertensive drugs or	Stop the infusion.<br>Other appropriate medications may include but are not limited to: epinephrine**, intravenous infusion of antihistamines, NSAIDS, paracetamol anesthetic, oxygen booster and corticosteroids. Strengthen the monitoring of the patient's vital signs according to their medical indications, until the researcher believes that the subject's condition is stable.<br>May require hospitalization.<br>**If an allergic reaction occurs, epinephrine should be used	No follow-up administration

ventilatory support.	immediately.<br>Subjects should be permanently stopped from further study drug treatment.	
Appropriate first aid equipment should be provided in the ward, and the physician should be contacted at any time during the administration period.<br>For further information, please refer to the Common Terminology Standard for Adverse Events (CTCAE) version 5.0 (http://ctep.cancer.gov).		

Other permissible dose adjustments of sintilimab

Under conditions other than treatment-related AEs, sintilimab treatment may be discontinued due to medical/surgical events or administrative reasons unrelated to the study. Subjects should restart the study treatment within 3 weeks after the interruption of medication, unless there are other discussions with the investigators. The reason for discontinuing medication should be recorded in the patient's study record.

5.7 Toxicities of chidamide

The safety data of chidamide in PTCL patients were mainly derived from a pivotal, single-arm, open-label, phase II clinical trial (n = 83) and an exploratory, single-arm, open-label, phase II clinical trial (n = 19). In pivotal Phase 2 clinical trials in PTCL, patients were treated with 30 mg twice weekly for an average of 4.4 months. In the exploratory phase II clinical trial of PTCL, patients in the two groups received 30 mg and 50mg, respectively, and patients in both groups took the drug twice a week for two weeks followed by a one-week rest period after discontinuation. The mean duration of treatment for all patients in this trial was 7.6 months. Common adverse reactions observed in clinical trials are hematological adverse reactions, including platelet count decreased, white blood cell or neutrophil count decreased, hemoglobin decreased; systemic adverse reactions, including fatigue, fever; gastrointestinal adverse reactions, including diarrhea, nausea and vomiting; metabolic and nutritional system adverse reactions, including decreased appetite, hypokalemia and hypocalcemia; and other adverse reactions, including dizziness and rash. Serious adverse events of the 83 patients in the pivotal Phase II clinical trial of chidamide, 7 patients (8.4%) experienced

8 serious adverse events (SAEs), including 1 patient each with increased white blood cell count, sudden cardiac death, decreased platelet count, lactic acidosis, intestinal perforation, and gangrene of the right toe, of which 3 (sudden cardiac death, lactic acidosis, and intestinal perforation) led to death.

For more information, please refer to the drug manual. In exceptional cases, oral antiemetics may be administered to prevent gastrointestinal reactions.

5.8 Premedication of chidamide

Prophylactic administration not recommended in drug manual. Oral antiemetics such as palonosetron may be used prophylactically.

5.9 Dose adjustment

Management of Hematologic Adverse Reactions and Dose Modifications

Withhold chidamide for Grade 3 or 4 neutropenia (neutrophil count < 1.0 × 10/L). Cytokines such as G-CSF should be administered if grade 3 neutropenia is present with body temperature above 38.5 ° C or grade 4 neutropenia. After the absolute neutrophil count recovers to 9 ≥ 1.5 × 10/L, the treatment can be continued: if the previous adverse reaction is grade 3, the original dose or dose can be reduced to 20 mg/time when the medication is resumed; if the previous adverse reaction is grade 4, the dose should be reduced to 20 mg/time when the medication is resumed.

For grade 3 or 4 thrombocytopenia, interleukin 11 or thrombopoietin (TPO) should be discontinued and blood component transfusion should be considered if platelet count is < 25.0 × 10/L or bleeding tendency occurs. After platelets recover to ≥ 75.0 × 10/L, treatment with HUMIRA can be continued. If the previous adverse reaction is grade 3, the original dose or dose can be reduced to 20 mg/time when the medication is resumed; if the previous adverse reaction is grade 4, the dose should be reduced to 20 mg/time when the medication is resumed.

For grade 3 or 4 anemia: Withhold chidamide, use erythropoietin (EPO) treatment; when hemoglobin < 5.0 g/dL, blood component transfusion should be performed. After hemoglobin recovers to ≥ 9.0 g/dL, treatment with HUMIRA can be continued: if the previous adverse reaction is grade 3, the original dose or dose can be reduced to 20 mg/time when medication is resumed; if the previous adverse reaction is grade 4, the dose should be reduced to 20 mg/time when medication is resumed. After treatment and dose reduction for the above hematological adverse reactions, if grade

4 hematological adverse reactions or grade 3 neutropenia with body temperature higher than 38.5°C recur, treatment with HUMIRA should be stopped. Management and dose adjustment of non-hematological adverse reactions If grade 3 non-hematological adverse reactions occur, the medication should be suspended and symptomatic treatment should be given.

5.10 Combined medication and non-drug treatment

5.10.1 Permissible combined drugs and non-drug therapies

Subjects are allowed to receive adequate supportive treatment during the clinical study, including blood transfusions and blood products, antibiotics, antiemetics, antidiarrheals, analgesics, and other applicable treatments in accordance with the guidelines of Ethics Committee. All combined medications or treatments should be recorded on the CRF. If there are changes during the study, including the dosage, frequency, route of administration, and date of administration of the drug are also recorded on the CRF.

If the patient takes nutritional supplements during the study, the investigator must be notified. Except for treatment after bone marrow transplantation, prophylactic colony stimulating factor should not be used in the first cycle. During the clinical trial, any drug and non-drug treatment (including physical therapy or blood transfusion) other than the research drug must be recorded in the CRF. All concomitant drugs used by the patient within 4 weeks before the first dose (screening period) and within 4 weeks after the final dose should be recorded.

The entries in the electronic medical record must include the dosage, schedule, route of administration, indications, and the use and discontinue dates of previous and combined drugs.

5.11.2 Prohibited drugs and non-drug therapies

Subjects should not receive any other investigative or anti-cancer treatment during the treatment period of this study, including chemotherapy, immunotherapy, biological

therapy, Chinese herbal medicine or endocrine therapy (with anti-tumor activity). Subjects are not allowed to receive palliative radiotherapy (except for relief of pain caused by bone metastases) during the period of receiving the research drug. Short-term or topical use of hormones for clinical conditions such as allergies is allowed. For non-neoplastic diseases, low-dose hormones (≤10mg of prednisone per day or equivalent hormones) can be used, but the use of glucocorticoids in other studies is not allowed (except for use as an antiemetic drug, ≤ 2 times per cycle).

5.12 Criteria for discontinuation of research drugs or exit of this trial

If any of the following occurs, the subject must stop the research drug:

1. Occurrence of treatment-related AEs that meet the discontinuation criteria specified in the study protocol.
2. Disease progression (subjects who meet the criteria for medication after disease progression can continue to be treated until the radiological evaluation confirms the progression and rules out the possibility of false progression).
3. During the study period, subjects receiving any prohibited combined treatments that affect the judgment of safety and efficacy can be required to stop the research drug after determined by the investigator and the sponsor's medical monitor.
4. Any intermittent disease that prevents the subject from continuing to receive research drug treatment.
5. The female subject is pregnant.
6. If the subject or his/her guardian is unwilling to continue the clinical research, he/she can propose to the doctor in charge at any time and discontinue the research drug immediately. According to the Declaration of Helsinki and other applicable laws and regulations, subjects have the right to withdraw from this

study at any time for any reason, and doctors or research centers must not be prejudiced in their future medical care.

7. Subjects who have poor compliance and fail to take medications on time and in amounts, when communication and coordination by the investigator is ineffective, or may cause significant deviations in the test results which cannot be remedied, can be determined to discontinue the research drug by the investigators.
8. Other situations where the investigator considers inappropriate to continue medication. Under any condition, the primary reason for discontinuing the research drug should be recorded in the original medical record. If the subject discontinues the research drug for any reason, for subjects who have received at least one research drug, the investigator shall make every effort to persuade the subject to accept the termination visit, and should continue to follow up those subjects with unresolved AEs.

5.13 Criteria for discontinuation of study drugs

If any of the following adverse events related to the study drug occur, the administration of research drug should be permanently discontinued:

1. Grade 3-4 pneumonia;
2. Grade 3-4 hepatitis (elevation of ALT, AST or total bilirubin);
3. Grade 3-4 decrease of cardiac function;
4. Grade 3-4 pancreatitis;
5. Grade 3-4 allergic reactions;

5.14 Treatment compliance

Patients will receive study drug treatment at the research center, under the close medical supervision of the investigator or authorized representative on the first day of

each cycle. Therefore, compliance assessment will not be performed.

6 Study progress

6.1 Screening period (Day -28 to Day 0)

Before starting any research-specific evaluation and operation, a written informed consent signed by the subject must be obtained.

A complete medical history should be recorded during screening:

1. Demographic data: including date of birth, gender, race/ethnicity; drinking history, smoking history.
2. Past medical history (except for this indication, including all past medical histories relevant to this study before signing the informed consent).
3. Past tumor history: including the date of diagnosis of the tumor, the start/end date of the previous treatment plan, the best treatment evaluation, and the date of disease progression; the radiotherapy history should include the start/end date and the location of the radiotherapy. Previous valuable operations (such as gastroscopy, needle biopsy and other diagnostic or therapeutic invasive operations) need to be recorded in the eCRF, including the start/end dates, the name and location of the operation.
4. Vital signs: including blood pressure, heart rate, respiration rate, body temperature. Blood pressure should be measured after the patient rests for 5 minutes.
5. Physical examination: including height (only for screening), weight, head, eyes, ears, nose, throat, neck, heart, chest (including lungs), abdomen, limbs, skin, lymph nodes, nervous system and general conditions of the patient.
6. ECOG score: It is recommended that the same researcher conduct ECOG evaluation during the entire study period. See Appendix II for details.

7. Laboratory examination: Specific laboratory examination indicators include hematology examination, blood biochemistry and urine examination.
   a) Hematology examination: red blood cell count, hemoglobin, hematocrit, reticulocyte count, white blood cell count and classification (neutrophils, lymphocytes, eosinophils, monocytes, basophils and other cells) and platelet count as well.
   b) Blood biochemical tests include total protein, albumin, blood sugar, total cholesterol, low-density lipoprotein, high-density lipoprotein, triglycerides, urea, creatinine, alkaline phosphatase, lactate dehydrogenase, total bilirubin, direct bilirubin, indirect bilirubin, AST, ALT, calcium, phosphorus, magnesium, potassium, sodium, chloride, serum amylase, uric acid.
   c) Urine routine test includes specific gravity, pH, urine sugar, protein, cast, ketone and haemocytes. During the screening period, if urine protein examination is more than ++ or judged by the doctor as abnormal and clinically meaningful, 24-hour urine protein quantification and 24-hour urine creatinine determination are required.
8. Histopathological testing.
9. 12-lead electrocardiogram.
10. Cardiac ultrasound examination (including evaluation of left ventricular ejection function).
11. Thyroid function.
12. Pulmonary function.
13. Serum pregnancy test (if applicable);
14. Serum virology test: including hepatitis B markers, HBV-DNA, HCV, HIV.

15. Bone marrow examination (smear ± biopsy).
16. Tumor evaluation: Assessed according to the RECIL 2017 lymphoma evaluation standard. See Appendix II. During the screening period, CT scan of the chest, abdomen and pelvis or PET/CT of whole body should be performed. If there are clinical indications, appropriate methods can be used to examine any other known or suspected locations of diseases, such as cranial MRI, bone scan.
17. Combined medication: Collect all medications during the screening period, including the generic name, the first dose, the reason for the medication, and the start and the end date of the medication.
18. Adverse events (AEs): AEs are collected during the screening period from the signing of the ICF until the first dose.
    i. Laboratory tests, 12-lead ECG, echocardiography, serum pregnancy test (if applicable), and bone marrow examination should be completed during day -7 to day 0.

6.2 Visits during the treatment period

1. Vital signs.
2. Physical examination.
3. Weight measurement.
4. Laboratory examination: Before each medication administration during the treatment period, the laboratory examination must be completed and the blood sampling time should not be earlier than 48 hours before the medication. Only when the investigator determined the laboratory examination results qualified can the subjects continue medication. During the treatment period, the investigator can increase the frequency of blood routine examinations

according to the conditions of the subjects.

5. Plasma EBV-DNA monitoring: Monitored on the first cycle of treatment and the first day of subsequent cycle. After all treatment courses are completed, monitored once a month at a fixed time.
6. 12-lead electrocardiogram: Evaluated every 3 weeks (±3 days) from the first administration.
7. Cardiac ultrasound examination (including evaluation of left ventricular ejection function): evaluation will be carried out every 12 weeks (±7 days) from the first administration.
8. Thyroid function: Evaluated every 3 weeks (±3 days) from the first administration.
9. Pulmonary function: Evaluated every 12 weeks (±7 days) from the first administration or there may be clinical needs.
10. Bone marrow examination (smear ± biopsy): Evaluated every 6 weeks (± 7 days) from the first administration for those with bone marrow infiltration or there may be clinical needs.
11. Tumor evaluation: Baseline and follow-up evaluations should adopt the same radiological methods and be performed by the same investigator as far as possible. Tumor assessment will be calculated according to the actual date of medication before each medication and will be evaluated every 6 weeks (-7 days), regardless of the effect of drug withdrawal. For patients who are suspected of developing PD before the next scheduled evaluation, an extra tumor evaluation should be performed.
12. Administration of the research drug: Administered every 3 weeks. If the subject cannot take the medicine at the planned time due to special reasons

(such as adverse reactions), a maximum delay of 14 days is allowed.

13. Record the combined medication;
14. Record AE.

6.3 End-of-treatment visit

The end-of-treatment visit is considered to be within 8 weeks after the end of the study. If the subject starts the follow-up anti-tumor treatment within this period, the end-of-treatment visit should be completed before then.

1. Vital signs.
2. Physical examination.
3. ECOG score.
4. Laboratory examination.
5. Histopathological test (if clinically required).
6. 12-lead electrocardiogram.
7. Cardiac ultrasound examination (including evaluation of left ventricular ejection function).
8. Thyroid function: Evaluated every 3 weeks (±3 days) from the first administration.
9. Pulmonary function: Evaluated every 12 weeks (±7 days) from the first administration or there may be clinical needs.
10. Serum pregnancy test (if applicable).
11. Bone marrow examination (smear ± biopsy): for bone marrow infiltration or there may be clinical needs.
12. Tumor evaluation.
13. Record the combined medication.
14. Record AE.

6.4 Follow-up of progression-free survival

Starting from 8 weeks after the last administration of the study drug, patients with stable or improving condition or those with early termination due to intolerance will be followed up for progression-free survival every 12 weeks (±7 days). Radiological examinations will be carried out until disease progression, death, withdrawal of ICF, start of other new treatments or end of the entire study (whichever occurs first).

6.5 Follow-up of overall survival

For patients who have already progressed and started other new treatments, survival follow-up will be carried out every 8 weeks (±7 days) and the following anti-tumor treatment information and survival data of the patients will be collected until 2 years after the first administration.

7 Response assessments

7.1 Outcome measures

7.1.1 Primary outcome measures

1. Responses were defined according to RECIL 2017 Response Criteria for Malignant Lymphoma including Objective Response Rate (ORR), Complete Remission Rate (CRR), Partial Remission Rate (PRR).
2. MTD of Chidamide: Maximum tolerable dose of chidamide among participants.
3. DLT of Chidamide: Dose-limiting toxicities of chidamide among participants.
4. RP2D of Chidamide: Recommended phase II dose (RP2D) of chidamide among participants.

7.1.2 Secondary outcome measures

1. Progression-Free Survival (PFS) time.

2. Duration of Response (DoR).
3. DoR: Among participants who experience an objective response, DoR is defined as the date of their first objective response (which is subsequently confirmed) to disease progression or among participants who experience an objective response.
4. Overall survival refers to the time from the beginning of randomization to death due to any reason.
5. The frequency and severity of adverse events (Adverse Event, AE) and the frequency of severe adverse events (Severe Adverse Event, SAE) (CTCAE v5.0).
6. To Explore biomarkers that may have predictive effects.

7.2 Evaluation of efficacy

The efficacy of the study drug will be determined by the objective tumor evaluation made by the research center based on the RECIL 2017 Response Criteria for Malignant Lymphoma.

Radiological evaluation of tumor is determined by PET/CT, MR or CT with uniformity in evaluation method as well as machine and technical parameters throughout the study period. Unless contraindications, contrast agents should be used. The radiological results are interpreted by researchers or radiologists in each research center. If the tumor evaluation has been performed within 21 days before enrollment with the same method and machine in the same hospital, it can be used as a baseline tumor evaluation. The baseline tumor evaluation should include the nasal cavity, chest, abdomen, pelvis and any other suspected areas. If there are clinical indications, appropriate methods, such as cranial MRI, bone scan can be used to examine any other known or suspected disease sites.

From the day of the first administration, radiological examination will be performed to evaluate the tumor condition every 6 weeks (-7 days) until the completion of 6 cycles of treatment, PD, intolerable toxicity, death, investigator's decision or patient's voluntary withdrawal from the study (whichever occurs first). Repeated every 8 weeks after 6 cycles up to 12 months, or disease progression, intolerable toxicity, death, investigators' decision, or the patient's voluntary withdrawal from the study Researchers can arrange additional radiological examinations based on the patient's clinical conditions. If the medication is suspended due to AE or other reasons, the tumor evaluation will still be carried out as originally planned. For patients who are suspected of developing PD before the next scheduled tumor evaluation, an unscheduled tumor evaluation should be performed.

8 Safety assessment

8.1 Safety Endpoint

Safety monitoring begins when the ICF is signed and continues until 4 weeks after the end of treatment or until the beginning of another anti-tumor treatment (whichever occurs first). The safety endpoints of this study are as follows:

• The incidence, type and severity of adverse events during treatment are classified according to NCI-CTCAE (version 5.0). Research-related TEAE, SAE, Grade 3 or higher non-hematological AE, Grade 4 hematological AE and permanent discontinuation due to TEAE.

• The frequency and causes of deaths that occurred within 4 weeks (±3 days) of the last medication.

• Safety laboratory inspections classified according to NCI-CTCAE (version 5.0).

• Vital signs, ECG, full physical examination and ECOG physical status score.

8.2 Evaluation of Adverse Events

8.2.1 Definition of adverse events

Adverse events refer to any adverse medical events that occur after exposure to a drug (including research drugs) while not necessarily have a causal relationship with the treatment. Therefore, AE can be any unfavorable and unexpected signs (including abnormal laboratory test results), symptoms or diseases temporally correlated to medication regardless of whether it is causally related to the treatment.

Adverse events during treatment refer to AEs that occurred after the first administration of research drugs.

Note: AE can therefore refer to any unfavorable and unexpected signs (including abnormal laboratory test results), symptoms or diseases (new or worsening) temporally correlated to medication. For marketed drugs, it also includes failure to obtain the expected benefits, abuse or misuse.

Events that meet the AE definition include:

1. Exacerbation of pre-existing chronic or intermittent diseases, including increased frequency and/or severity.
2. A newly discovered or diagnosed disease after the trial treatment, although the disease may have existed before the start of the study.
3. Suspected signs, symptoms or clinical sequelae of drug interactions.
4. Suspected signs, symptoms or clinical sequelae of trial treatment or concomitant overdose (overdose itself will not be reported as AE/SAE).
5. "Lack of efficacy" or "failure to achieve the expected pharmacological effect" itself is not reported as an AE or SAE. However, any signs and symptoms and/or clinical sequelae caused by "lack of efficacy" will be reported as an AE or SAE if they meet the corresponding definition.

Events that do not meet the definition of AE include:

1. Internal medicine or surgery (for example, endoscopy and appendectomy) while the conditions leading to the above surgery are determined as AEs.
2. The absence of adverse medical events (social and/or environmental facilitating admission).
3. Pre-existing disease or condition at the beginning of the study, with predictable fluctuations that did not aggravate during the study period.
4. The studied disease/abnormality or the expected progression, signs or symptoms of the disease/abnormality under study, unless it is more severe than expected.
5. The collection of AE should start from signing the informed consent form until 30 days after the medication.

8.2.2 Definition of serious adverse events

Serious adverse events were the following AEs at any dose:

a) Results in death.

b) Life threatening.

c) Note: The term "life-threatening" in the definition of "serious" means that the patient is at risk of death when the event occurs, and does not mean death if the event is more serious.

d) Need to be hospitalized or extend the length of hospitalization.

Note: Generally speaking, hospitalization refers to patients staying in a hospital or emergency ward (generally at least overnight) and/or receiving treatment that is not suitable for conduction in doctor's office or outpatient department. Complications that occurred during hospitalization were AEs. If the complication prolongs the hospital time or meets any other serious criteria, the event is an SAE. When it is uncertain whether it should be "hospitalized" or required for "hospitalization", the AE

should also be regarded as an SAE.

Hospitalization due to selective treatment or pre-existing disease that has not worsened than baseline is not an AE.

e) Results in disability/dysfunction.

Note: The term "disability" refers to severe influence of an individual's ability to conduct normal life functions. This definition does not include events of relatively little clinical significance, such as simple headaches, nausea, vomiting, diarrhea, flu and accidental trauma (such as ankle sprains), which may affect daily function but dose not cause significant loss of function.

f) Congenital malformations/birth defects.

g) In other cases, medical or scientific judgment is required to determine whether to report. For example, it may not immediately threaten life or cause death or hospitalization, but it may threaten the patient or may require medical or surgical intervention to prevent the other important medical events listed in the above definition. These should also be considered SAEs, such as invasive or malignant tumors, allergic bronchospasm with intensive treatment in the emergency room or at home, blood disorders, convulsions that did not lead to hospitalization or drug dependence/abuse.

8.2.3 Laboratory inspections and other safety assessment abnormalities reported as adverse events and serious adverse events

Any abnormal laboratory test results (hematology, clinical biochemistry, urinalysis) or other safety assessments (for example, ECG, imaging scans, vital signs measurement), including worsening than baseline and events of clinical significance with the researcher's medical and scientific judgments will be reported as AE or SAE.

In addition, if any laboratory test results or other safety assessments lead to interventions (including permanent discontinuation of trial treatment, dose reduction,

and/or administration suspension/delay), the relevant AE or SAE should be recorded.

Any new primary cancer must be reported as SAE.

However, for any clinically significant safety assessment related to the underlying disease, unless the result is judged by the investigator more serious than expected, should not be reported as an AE or SAE.

8.2.4 Assessment of the severity of adverse events

Researchers need to follow the definition of severity in accordance with NCI-CTCAE (version 5.0) to describe the highest severity of AE. If the AE is judged as SAE, the CTCAE classification recorded on the AE page of the eCRF must be consistent with the description of the NCI-CTCAE (version 5.0) classification.

Standard of Adverse Event Severity

Grades	Equivalent	Description
1	Mild	Observation only and normal daily activities not affected.
2	Moderate	Daily activities affected; no treatment or medical intervention indicated although they can relieve symptoms or improve life quality.
3	Severe	Inability to work or complete daily activities; treatment or medical intervention indicated to improve the quality of life or relieve symptoms; not immediately life-threatening
4	Life threatening or disability	Direct life threatening or permanent mental or physical damage; inability to work or carry out daily activities; urgent medical intervention indicated.
5	Death	Adverse events result in death.

Pay attention to the difference between intensity and severity of AE. Serious AE is not necessarily SAE. For example, a headache can be severe (significantly affecting the patient's daily function) while not necessarily SAE, unless in agreement with one of the above SAE criteria.

8.2.5 The causal relationship between adverse events and study drugs

The causal relationship between adverse events and the study drug is as follows:

• Definitely irrelevant: No study drug is used, or there is no correlation between the use of study drug and the time of the AE, or there is a clear cause of the AE.

• Possibly irrelevant: There is evidence of the use of the study drug and the AE that occurred may be better explained by other reasons. The withdrawal reaction is negative or unclear.

• Possibly related: There is evidence of the use of the study drug and the temporal correlation between the occurrence of AE and the use of study drug is reasonable. AE can be explained by other reasons. The drug withdrawal reaction was positive.

• Definitely related: There is evidence of the use of study drugs. The occurrence of AEs and the use of study drugs are in a reasonable time sequence. AEs explained by study drugs are more reasonable than other reasons. The drug withdrawal reaction was positive and the reaction is positive for repeated medication (if feasible).

• Unable to judge

8.2.6 Handling and reporting of adverse events

According to the Chinese GCP regulations, all AEs that occur during clinical research should take the following measures, regardless of which treatment group they belong to or whether related to the study drug:

1. The investigator should immediately take appropriate protective measures to ensure the safety of the patient and relevant experts should make a diagnosis and state the reason.
2. If the study is terminated, the investigator should also check the patient regularly and fill in the date of termination of the study (the date when the administration of the study drug was stopped), the reason for termination and the detailed process in the CRF.

3. The investigator should follow up all AEs that occur until any of the following condition occurs:
   a) AE is alleviated or improved to the baseline level.
   b) The researcher confirms that no further improvement is expected.
   c) Death of the patient.
   d) The patient has lost contact.
   e) The investigator confirms that the AE is not related to the trial treatment.
   f) The patient starts a new anti-cancer treatment.
   g) The study is over.

Any AE should be recorded in detail on the eCRF and reported in the clinical research report.

If a large number of unexpected AEs related to the study drug occur during the research period, it is required to immediately report to the Institutional Review Board (IRB)/Independent Ethics Committee (IEC), the Chinese Food and Drug Administration (CFDA) and relevant supervision and management department of provinces, autonomous regions, and municipalities.

8.2.7 Handling and report of serious adverse events

According to the Chinese GCP regulations, all SAEs that occur during clinical research, in addition to the treatment of AEs in Section 8.2.6, the following measures should also be taken:

1. The investigator must report any SAE (whether it is considered to be related to the study drug or not) within 24 hours and record it on the AE page of the CRF.
2. All SAEs should be followed up as far as possible until alleviation,

improvement to baseline level, death or loss of contact of the patient. SAEs that are considered as irrelevant to the study treatment by the research still require follow-up. After the follow-up period is over, with enough reasons to rule out relevance of SAE to the study drug, it should still be reported in the same way.

8.2.8 Pregnancy

In order to ensure the safety of patients, pregnancy that occurs during the trial treatment must be reported to the investigator within 24 hours of notification. At the same time, the pregnancy must be visited to clarify the outcome, including spontaneous or induced abortion, details of childbirth, congenital malformations or comorbidities of the mother or newborn.

After the pregnancy occurs, the researcher must fill out the pregnancy report to record the details of the pregnancy and its visits. SAE during pregnancy should also be reported on the SAE report. The investigator should report to Innovent within 24 hours of notification of pregancy and fill out the "Innovent Clinical Trial Pregnancy Report/Follow-up Form".

If a male patient participates and receives the study drug in the trial, his female partner becomes pregnant, the investigator should report to Innovent within 24 hours of notification of pregnancy, fill out the "Innovent Clinical Trial Pregnancy Report/Follow-up Form" and collect information of pregnancy outcome. Prior to this, the male subject and his female partner must sign an informed consent form agreeing to provide the pregnancy outcome information.

8.3 Safety assessment of laboratory and other inspections

Laboratory examination indicators include hematology, blood biochemistry, urine routine examination, histopathology, serum virology examination and bone marrow

biopsy, all of which must be carried out in a laboratory accredited by the hospital. The normal range of all laboratory test items, as well as the updates during the research period, should be recorded on the corresponding page of CRF.

8.4 Suitability of measurement

All effectiveness and safety assessments used in this study are standard assessments, which are widely used and recognized as reliable, accurate and relevant assessments.

9 Data management

In this study, patient data will be recorded into the designated CRF and sent to the data system confirmed by the researcher for integration with data from other sources.

The staff of the research center shall be responsible for filling in the CRF. For all patients who have signed the ICF and screened qualified to participate in the study, the investigator or authorized staff must carefully record the items in the CRF and ensure no blank or missing items (unrecorded blanks should be filled in according to the actual situation). All data in the CRF must be checked with the original data of the patient to ensure that it is correct.

The investigator must paste the original or copy laboratory sheets on the patient's research medical record. For abnormal laboratory or examination data, the investigator must verify and clarify whether it has clinical significance. The investigator should strictly follow the CRF instructions to fill in the form.

10 Statistics and statistical analysis

All statistical analysis will be performed with SPSS (18.0 or higher version) software. In this study, the variables obtained at each observation time were statistically described, unless the plan clarifies no need for statistical description at a particular time point. The P value less than or equal to 0.05 is judged as a criterion for statistically

significance. The description of quantitative indicators will calculate the mean, standard deviation, median, minimum and maximum. The description of classification indicators includes the number of cases and percentages of various types. Categorical variables will be statistically described using the frequency and percentage of each category. The final analysis of the study will be based on the data collected throughout the entire study.

10.1 Calculation of sample size

Critical clinical data:

1. **Efficiency of anti-PD-1 antibody**

Currently, three literatures were published reporting anti-PD-1 antibody to treat relapsed and refractory NKT cell lymphoma, with a total of 15 patients. The curative effects of the three articles are as follows:

Short-term efficiency:

	N	ORR	CR	PR
Hong Kong, China	7	100%(7)	28.6%(2)	71.4%(5)
Henan, China	7	57.2%(4)	28.6%(2)	28.6%(2)
Nanchang, China	1	100%(1)	100%(1)	
Summary	15	80%(12)	33.3%(5)	46.7%(7)

Long-term survival: no report.

2. **Efficiency of Chidamide**

Short-term efficiency

	N	ORR	CR	PR
Refractory and Relapsed	20	60%(12)	30%(6)	30%(6)

Long-term survival: Median PFS = 7.0 (months)

Therefore, if ORR (Objective Effective Rate) is used as the primary outcome measures, the sample size is as follows:

- The phase Ib study used a “3+3” dose escalation scheme to determine the

maximum tolerated dose (MDT) of chidamide. There are three dose groups (20mg, 25mg, 30mg) for chidamide, with 3 to 6 patients in each dose group. **Approximately 9 to 18 patients are needed for stage Ib**.

- The phase II study adopts the simon two-stage optimal design method. The main purpose of the research is to use the maximum tolerated dose determined in phase Ib as the recommended dose to preliminarily evaluate the ORR of treatment of chidamide combined with anti-PD-1 antibody in the treatment of refractory and relapsed Extranodal NK/T-cell lymphoma. According to previous studies, P1 is supposed as 0.8 (expected effective rate) and P0 as 0.55 (poor effective rate), with α=0.05 and β=0.8. The best two-stage design is (4/7, 19/28). That is, 7 patients were enrolled in the first stage, and if only 4 or fewer patients were effective, the trial was terminated. Otherwise, the trial enters the second phase and 21 more patients will be enrolled. That is, a total of 28 patients will be enrolled in the phase II study. A total of 37 to 46 patients will be enrolled in stage Ib and II. The study loss rate was set at 10% and a total of 40-50 patients will be enrolled. If the number of effective cases in all patients in the two stages exceeds 19, there is an 80% probability that the test drug is effective.

10.2 Analysis of the population

Per protocol set: According to the principle of intentional analysis, the subjects will be eliminated with the least and reasonable method. The full analysis set refers to any patient who has received at least one complete treatment and has a corresponding efficacy evaluation.

As-treated set: The safety analysis population refers to all patients who have entered the study, have received at least one complete treatment and have appropriate follow-up information to provide safety analysis. All safety information records from

patients will be evaluated, including adverse events and laboratory test results.

Primary outcome measures

The objective remission of the primary efficacy endpoint evaluation refers to the CR, PR or MR judged by the investigator according to the RECIL 2017 Response Criteria for Malignant Lymphoma. Subjects who do not meet the above criteria, including those who have not undergone any post-baseline tumor assessment, will be evaluated as non-remission.

ORR (CR+PR) refers to the proportion of subjects who have an objective remission. The population participating in the analysis is the subjects who have been confirmed by the investigator to have evaluable or measurable lesions in the efficacy analysis.

The proportion of subjects and the corresponding 95% CI will be summarized according to the remission categories (CR, PR, SD and PD) judged by the investigator. This analysis will be determined by the investigator in the efficacy analysis set of subjects who have evaluable or measurable lesions at baseline. At the same time, the remission rate and remission category evaluated by the investigator will be summarized in the proportion of subjects and the corresponding 95% CI. This analysis will be determined by the investigator in the efficacy analysis set of subjects who have evaluable or measurable lesions at baseline.

Secondary outcome measures

PFS refers to the time (in months) from the first medication to the first confirmation and recording of disease progression or death (whichever comes first). The researcher will evaluate the disease progression according to the RECIL 2017 Response Criteria for Malignant Lymphoma. During the analysis, subjects without disease progression or death will be censored on the date of the last tumor evaluation.

Subjects who have no post-baseline tumor assessment results will be censored on the first day of medication plus 1 day.

DoR refers to the time from the date of first reaching CR or PR (whichever is recorded first) to the date of first recording of disease progression or death (whichever comes first). DoR assessments were performed on subjects judged by the investigator as objective remission. During the analysis, subjects who have no disease progression or death will be censored on the last day of tumor assessment. If the tumor assessment has not been performed after the date of the first achievement of CR or PR, the date of the first occurrence of CR or PR plus 1 day is used as the censored value when calculating DoR. Kaplan-Meier method is adopted to analyze DoR and obtain survival curve.

TTR refers to the time from the first medication to the first CR or PR (whichever is recorded first). TTR will only include subjects who achieve ORR. The TTR will be summarized separately for the researcher's judgment.

OS refers to the time from the first medication to death of whatever reason. Subjects who have not been reported dead before the cut-off date will be censored on the last known survival day. If the subject has no post-baseline data, it will be censored on the day of the first medication plus 1 day. The analysis method is the same as PFS.

10.3 Safety analysis

The safety analysis is evaluated by a summary of TEAE, changes in laboratory test results, ECG or cardiotoxicity evaluation indicators and changes in vital signs among the specific population.

Adverse events will be summarized according to the Medical Dictionary for Regulatory Activities (MedDRA, version 19.1) and displayed in accordance with the system organ classification and selected terminology. At the same time, the relevance

between SAE and AE, the severity of AE and a summary of some special events are listed separately.

Changes in laboratory test results will be summarized according to the NCI-CTCAE (version 5.0). For laboratory indicators, the maximum toxicity that occurred during the study period is summarized in the form of counts and percentages. The changes in vital signs and ECOG scores will be compared with baseline levels and carried out descriptive statistics. A summary of vital signs, ECG, laboratory and physical examination will be given in a chronological order.

10.4 Efficacy analysis

The researcher can decide the patient's treatment plan based on the efficacy assessment made by the research center. Please refer to Chapter 7.1 for the definition of each efficacy endpoint. The confirmed ORR and the 95% Clopper Pearson confidence interval should be calculated. In addition, other efficacy endpoints, including PFS, DCR, DoR and OS should also be evaluated.

11 Study management

This study will strictly abide by the laws and regulations related to clinical trials in China, including GCP, the Declaration of Helsinki (2008 Edition) and other regulations, as well as the implementation of this research plan. The details of the research implementation will be restricted by the standard operating procedures (SOP) of all parties involved in the research.

11.1 Ethical considerations

The research protocol, ICF, other written information and/or other materials provided to the patient must be submitted to the IEC for approval before the start of the research. IEC will strictly abide by the requirements of relevant laws and regulations to examine these materials and issue ethical documents after approval. Only after

receiving the approval from the IEC can the research be proceeded.

During the research process, any important modifications to the plan must also be reviewed and approved by the IEC before they can be implemented.

11.2 Informed Consent

The investigator or its designated representative will be responsible for explaining the research background, the pharmacological characteristics of the research drug, the research plan and the benefits and risks for participating in the research to each patient, the patient's legal representative or impartial witness. Before entering into the research (before the screening examination), the informed consent signed by the patient or his legal representative and the research physician should be obtained.

The final ICF document should include the following content:

a) Research purpose, research procedures, patient's obligations, foreseeable benefits and risks and inconveniences for participating in the research;

b) Available treatment in the event of research-related damage and appropriate insurance compensation;

c) Access to research data and confidentiality of patient information, etc.

ICF should be written in a language that patients can read and obtain written approval from relevant management departments in accordance with laws and regulations and write them.

Both of the patient or his legal representative and the researcher or his representative who performs the informed consent process are required to sign on the ICF and sign the date. The original ICF should be kept by the investigator and the patient each. If important new data related to research drugs are observed, the ICF must be revised in written form and sent to the relevant management department for approval. Informed consent will be obtained again.

11.3 Compensation for patient's health damage

Chidamide used in this clinical study is an approved marketing drug by the Chinese national health administration and has been clinically widely used for a long time. Sintilimab has now passed a variety of clinical studies on tumors and proved to be safe. Therefore, the safety of the chemotherapy regimen in this study is relatively high. However, in the process of clinical research, once research-related damage occurs, the investigator will conduct active diagnosis and treatment. In addition, in order to ensure patients' rights, the sponsor has also purchased relevant medical insurance and will give corresponding financial compensation to patients with serious injuries (except for those caused by medical liability errors and accidents) according to laws and regulations and the professional judgment of clinicians.

11.4 Quality control and quality assurance

In order to ensure the quality of the research, the researcher should discuss and formulate a clinical research plan before the actual research starts. All relevant researchers participating in the study should receive GCP training.

Each research center must manage research medications in accordance with SOPs, including receiving, custody, distribution and recycling.

11.5 Modification of the study protocol

Any important amendments to this scheme must be approved by the researcher in writing before implementation, submitted to the IEC for approval and sent to the GCP for filing.

11.6 Termination of the study

The researcher reserves the right to decide whether the research should be stopped. If the researcher voluntarily interrupts the research, the IEC should be notified immediately and be provided with a detailed written explanation on the termination or

interruption of the research. Study records must be kept.

11.7Summary report of the research

After the research is over, the researcher should objectively summarize the research results and statistically analyze the research data with appropriate statistical methods. An objective evaluation of the safety of the drug based on the results could therefore be made.

11.8 Confidentiality and publication of research results

Researchers should keep the information and data related to this research confidential and may not quote or publish relevant research results or materials in advance without authorization.

12 Appendices

12.1 Appendix 1. Eastern Cooperative Oncology Group Performance Status

Performance status will be assessed using the ECOG performance status grades as indicated below:

**Grade**	**Definition**
**0**	Fully active, able to carry on all pre-disease performance without restriction
**1**	Restricted in physically strenuous activity but ambulatory and able to carry out work of a light or sedentary nature, e.g., light housework, office work
**2**	Ambulatory and capable of all self-care but unable to carry out any work activities; up and about more than 50% of waking hours
**3**	Capable of only limited self-care; confined to bed or chair more than 50% of waking hours
**4**	Completely disabled; cannot carry on any self-care; totally confined to bed or chair
**5**	Dead

12.2 Appendix 2. RECIL 2017:Response categories based on assessment of target lesions

	% Change in sum of diameters of target lesions from nadir				
	CR	PR	MR [a]	SD	PD
% Change from baseline	• Complete disappearance of all target lesions and all nodes with long axis<br>• 30% decrease in the sum of longest diameters of target lesions (PR) with normalization of FDG-PET	30% decrease in the sum of longest diameters of target lesions but not a CR	10% decrease in the sum of longest diameters of target lesions but not a PR (<30%)	<10% decrease or < 20% increase in the sum of longest diameters of target lesions	• >20% increase in the sum of longest diameters of target lesions<br>• For small lymph nodes measuring <15 mm post therapy，a minimum absolute increase of 5 mm and the long diameter should exceed 15 mm<br>• Appearance of a new lesion
FDG-PET	Normalization of FDGPET (Deauvile score 1-3)	Positive (Deauville score 4-5)	Any	Any	Any
Bone marrow involvement	Not involved	Any	Any	Any	Any
New lesions	No	No	No	No	Yes or No

CR, complete response; CT, computerized tomography; FDG-PET, [18F]2-fluoro-2-deoxy-D-glucose; MR, minor response; PD, progression of disease; PR, partial response; SD, stable disease. [a] A provisional category.
